# Integrative Analyses of Genes Associated with Subcutaneous Insulin Resistance

**DOI:** 10.3390/biom9020037

**Published:** 2019-01-22

**Authors:** Manoj Kumar Pujar, Basavaraj Vastrad, Chanabasayya Vastrad

**Affiliations:** 1 Department of Medicine, Pooja Hospital, Davangere 577002, Karnataka, India; manoj.pujar@gmail.com; 2 Department of Pharmaceutics, SET`S College of Pharmacy, Dharwad 580002, Karnataka, India; basavarajmv@gmail.com; 3 Biostatistics and Bioinformatics, Chanabasava Nilaya, Bharthinagar, Dharwad 580001, Karanataka, India

**Keywords:** insulin resistance, protein–protein interaction, modules, non-insulin-dependent diabetes mellitus, obesity

## Abstract

Insulin resistance is present in the majority of patients with non-insulin-dependent diabetes mellitus (NIDDM) and obesity. In this study, we aimed to investigate the key genes and potential molecular mechanism in insulin resistance. Expression profiles of the genes were extracted from the Gene Expression Omnibus (GEO) database. Pathway and Gene Ontology (GO) enrichment analyses were conducted at Enrichr. The protein–protein interaction (PPI) network was settled and analyzed using the Search Tool for the Retrieval of Interacting Genes (STRING) database constructed by Cytoscape software. Modules were extracted and identified by the PEWCC1 plugin. The microRNAs (miRNAs) and transcription factors (TFs) which control the expression of differentially expressed genes (DEGs) were analyzed using the NetworkAnalyst algorithm. A database (GSE73108) was downloaded from the GEO databases. Our results identified 873 DEGs (435 up-regulated and 438 down-regulated) genetically associated with insulin resistance. The pathways which were enriched were pathways in complement and coagulation cascades and complement activation for up-regulated DEGs, while biosynthesis of amino acids and the Notch signaling pathway were among the down-regulated DEGs. Showing GO enrichment were cardiac muscle cell–cardiac muscle cell adhesion and microvillus membrane for up-regulated DEGs and negative regulation of osteoblast differentiation and dendrites for down-regulated DEGs. Subsequently, myosin VB (MYO5B), discs, large homolog 2(DLG2), axin 2 (AXIN2), protein tyrosine kinase 7 (PTK7), Notch homolog 1 (NOTCH1), androgen receptor (AR), cyclin D1 (CCND1) and Rho family GTPase 3 (RND3) were diagnosed as the top hub genes in the up- and down-regulated PPI network and modules. In addition, GATA binding protein 6 (GATA6), ectonucleotide pyrophosphatase/phosphodiesterase 5 (ENPP5), cyclin D1 (CCND1) and tubulin, beta 2A (TUBB2A) were diagnosed as the top hub genes in the up- and down-regulated target gene–miRNA network, while tubulin, beta 2A (TUBB2A), olfactomedin-like 1 (OLFML1), prostate adrogen-regulated mucin-like protein 1 (PARM1) and aldehyde dehydrogenase 4 family, member A1 (ALDH4A1)were diagnosed as the top hub genes in the up- and down-regulated target gene–TF network. The current study based on the GEO database provides a novel understanding regarding the mechanism of insulin resistance and may provide novel therapeutic targets.

## 1. Introduction

Obesity is a major risk factor for non-insulin-dependent diabetes mellitus (NIDDM), which has a prevalence that is positively correlated with body mass index [1]. The NIDDM/Type 2 diabetes is a complicated metabolic disorder which arises from a complex interaction of a vast number of genetic and environmental factors [2]. Insulin resistance is the condition in which the cells of the muscles, fat, and liver do not respond well to insulin and do not easily take up glucose from blood [3]. Obesity, NIDDM, hypertension, atherosclerotic cardiovascular disease, ayslipidemia, and hyperinsulinemia are the most common and severe disorders related to insulin resistance [4]. High plasma free fatty acid concentrations are typically linked with many insulin-resistant states, including obesity and NIDDM [5]. Modified fatty acid metabolism contributes to insulin resistance in patients with non-insulin-dependent diabetes mellitus [6]. High secretion of tumor necrosis factor-alpha (TNF-α) [7], interleukin-6 (IL-6) [8], monocyte chemoattractant protein-1 (MCP-1) [9], and additional products of macrophages [10] by adipose tissue with low sensitivity to insulin has also been detected in obesity.

Accumulating evidence has demonstrated that signaling pathways and genes are associated with insulin resistance [11,12]. High expression of Retinol Binding Protein 4 (RBP4) is responsible for the development of insulin resistance in obesity and NIDDM [13]. Polymorphism in Pacific Crossing 1 (PC-1) strongly linked with insulin resistance [14]. Alteration in Adiponectin receptor 1 (ADIPOR1) and Adiponectin receptor 2 (ADIPOR1) ADIPOR2 is important for development of insulin resistance in NIDDM [15]. Low expression of FOXC2 is identified with insulin resistance [16]. The activation of the peptidase inhibitor 3 (PI3)-kinase- and mitogen activated protein (MAP) -kinase-mediated signaling pathways can cause insulin resistance in muscle [17]. Activation of inhibitor of nuclear factor kappa-B kinase subunit beta (IKK-β) is important for the improvement of insulin resistance in obesity [18]. Improper activation of the tuberous sclerosis complex (TSC)/ Ras homolog enriched in brain (Rheb)/mechanistic target of rapamycin kinase (MTOR) signaling pathway leads to insulin resistance [19]. Stimulation of the JNK pathway has an important role in the advancement of insulin resistance in obesity [20].

However, due to the lack of effective early diagnostic methods and limited therapeutic options for preventing insulin resistance development in obesity and non-insulin-dependent diabetes mellitus, the real outcome for patients with insulin resistance still needs to be improved. Therefore, it is essential to understand the precise molecular mechanisms associated with the development of insulin resistance and thus develop effective early diagnostic and therapeutic strategies.

During the last few decades, microarray technology and bioinformatics analysis have been widely used to screen genetic modifications at the genome level, which has helped us diagnose the differentially expressed genes (DEGs) and functional pathways associated with the development of insulin resistance. In the current study, a messenger RNA (mRNA) microarray dataset (GSE73108) from the gene expression omnibus (GEO) was downloaded and analyzed in order to obtain DEGs between omental adipose tissue with insulin resistance and subcutaneous adipose tissue with insulin resistance. Subsequently, gene ontology (GO), pathway enrichment analysis, protein–protein interaction (PPI) network analysis, module analysis, target gene–miRNA networks and target gene–transcription factors (TF) networks were investigated to help us understand the molecular mechanisms underlying the progression of insulin resistance.

## 2. Materials and Methods

### 2.1. Microarray Data

Gene Expression Omnibus (http://www.ncbi.nlm.nih.gov/geo) [21] is a public functional genomics data repository of high-throughput gene expression data, chips, and microarrays. A gene expression dataset (GSE73108) was downloaded from GEO (Illumina HumanHT-12 V4.0 expression bead chip GPL10558 Platform, San Diego, CA, USA); this dataset was submitted by Wentworth et al. [22]. The probes were transformed into the corresponding gene symbol according to the annotation information in the platform. The GSE73108 dataset contained six omental adipose tissue samples with insulin resistance and six subcutaneous adipose tissue samples with insulin resistance.

### 2.2. Identification of Differentially Expressed Geness

The DEGs between omental adipose tissue samples with insulin resistance and subcutaneous adipose tissue samples with insulin resistance were screened using the limma (linear models for microarray data) package of R software (version 3.5.1) [23]. The Benjamini and Hochberg false discovery rate (FDR) was applied to provide a balance between the discovery of statistically significant genes and the limitations of false positives. An absolute value of log FC (fold change) > 1.24 was the cutoff for up-regulated genes, log FC (fold change) >−0.725 was the cutoff for down-regulated genes, and *p* < 0.05 was considered statistically significant.

### 2.3. Pathway Enrichment Analyses of Differentially Expressed Genes

Enrichr (http://amp.pharm.mssm.edu/Enrichr/) [24] is an online biological information database that integrates biological databases (Kyoto Encyclopedia of Genes and Genomes (KEGG) (http://www.genome.jp/kegg/pathway.html) [25], WikiPathways (https://www.wikipathways.org) [26], BioCarta (https://cgap.nci.nih.gov/Pathways/BioCarta_Pathways) [27] HumanCyc (https://humancyc.org/) [28], Panther (http://www.pantherdb.org/pathway/) [29] and NCI-Nature (http://pid.nci.nih.gov/) [30]) and analysis tools and provides a comprehensive set of functional annotation information on genes and proteins for users to extract biological information. These databases are resources for understanding high-level functions and biological systems from large-scale molecular datasets generated by high-throughput experimental technologies [31]. A value of *p* < 0.05 was considered statistically significant.

### 2.4. Gene OntologyEnrichment Analyses of Differentially Expressed Genes

In order to investigate the underlying function of DEGs, we employed the Enrichr [24] online tool for gene ontology (GO) (http://www.geneontology.org/) [32] enrichment analysis; it includes biological processes (BPs), cellular components (CCs), and molecular functions (MFs). A value of *p* < 0.05 was considered statistically significant.

### 2.5. Construction of a Protein–Protein Interaction Network and Topological Analysis

The Search Tool for the Retrieval of Interacting Genes (STRING) (http://www.string-db.org/) [33] is an online database implementing experimental and predicted PPI information. In this investigation, the STRING database was used to evaluate the PPIs among the proteins encoded by the DEGs with a combined score of >0.4; then, the PPI networks for the up-regulated and down-regulated genes were independently envisioned using Cytoscape software (http://www.cytoscape.org/) [34]. Network topological properties were used to analyze and compare the network. The network topological properties which were analyzed include node degree [35], betweenness centrality [36], stress centrality [37], closeness centrality [38], and cluster coefficient [39].

### 2.6. Module Analysis

The PEWCC1 is an automated algorithm which can be used as an integrated plugin in Cytoscape and which provides a way to establish highly connected dense modules in a PPI network [40]. The interconnected genes in the modules were diagnosed and selected for further analysis based on the number of genes. We used *n* > 10 as a parameter for selecting highly interconnected modules.

### 2.7. Construction of the Target Gene–MicroRNA Network

MicroRNAs control the expression of genes in a disease condition by interacting with their target genes at the post-transcription phase [41]. In the current study, the miRNAs associated with DEGs were searched using the NetworkAnalyst (https://www.networkanalyst.ca/) [42] online tool which integrates microRNA databases such as TarBase (http://diana.imis.athena-innovation.gr/DianaTools/index.php?r=tarbase/index) [43] and miRTarBase (http://mirtarbase.mbc.nctu.edu.tw/php/download.php) [44], and the target gene–miRNA network was visualized using Cytoscape software [34].

### 2.8. Construction of the Target Gene–Tanscription FactorNetwork

Transcription factors control the expression of genes in a disease condition by interacting with their target genes at the transcription phase [45]. In the current study, the TFs associated with DEGs were searched using the NetworkAnalyst [42] tool which integrates TF database JASPAR (http://jaspar.genereg.net/) [46], and the target gene–TF network was visualized using Cytoscape software [34].

## 3. Results

### 3.1. Identification of Differentially Expressed Genes in Insulin Resistance

**Figure 1:**
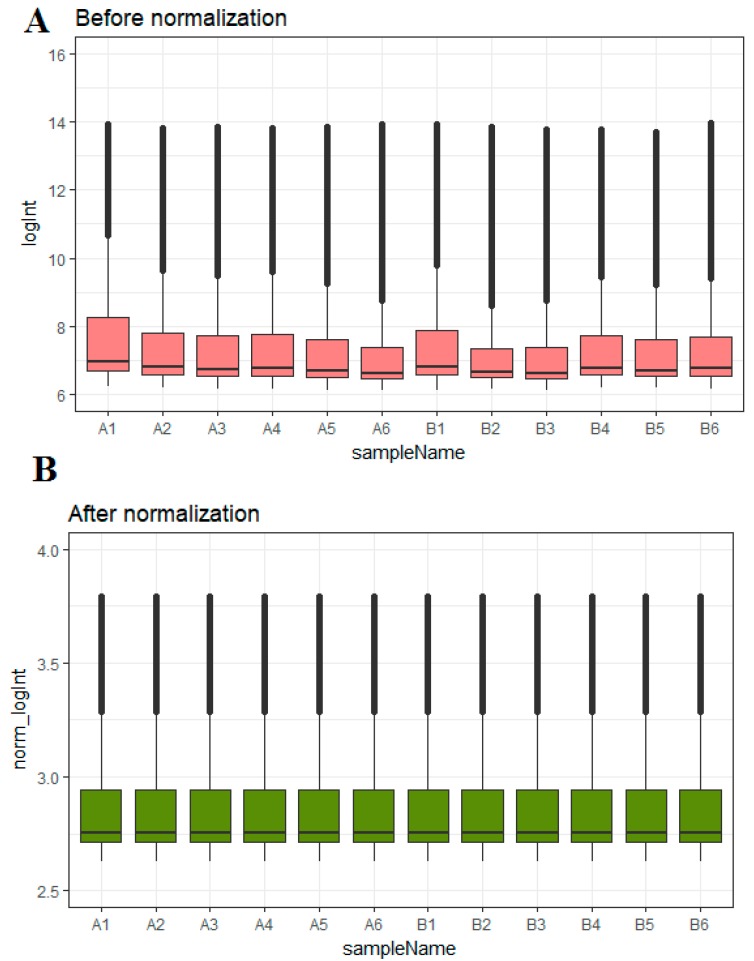
Box plots of the gene expression data before and after normalization. The horizontal axis represents the sample symbol and the vertical axis represents the gene expression values. The black line in the box plot represents the median value of gene expression. (A1, A2, A3, A4, A5, A6 = omental adipose tissue samples; B1, B2, B3, B4, B5, B6 = subcutaneous adipose tissue samples.)

A box plot of the data before and after normalization is shown in Figure 1A,B.

**Figure 2:**
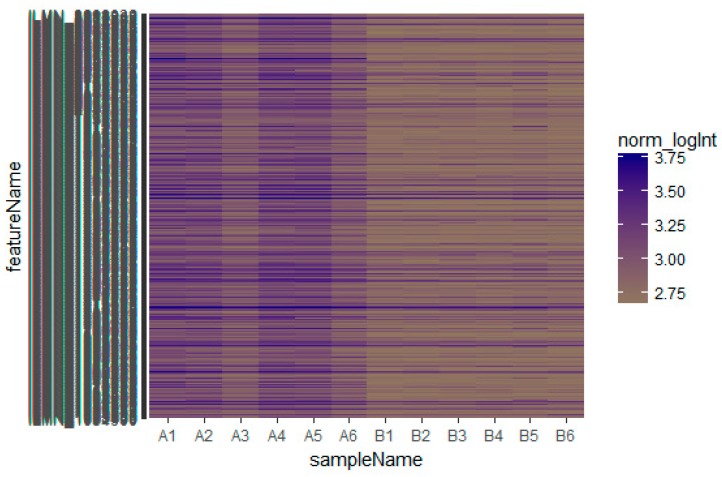
Heat map of up-regulated differentially expressed genes. The legend on the top left indicates the log fold change of genes. (A1, A2, A3, A4, A5, A6 = omental adipose tissue samples; B1, B2, B3, B4, B5, B6 = subcutaneous adipose tissue samples.)

**Figure 3:**
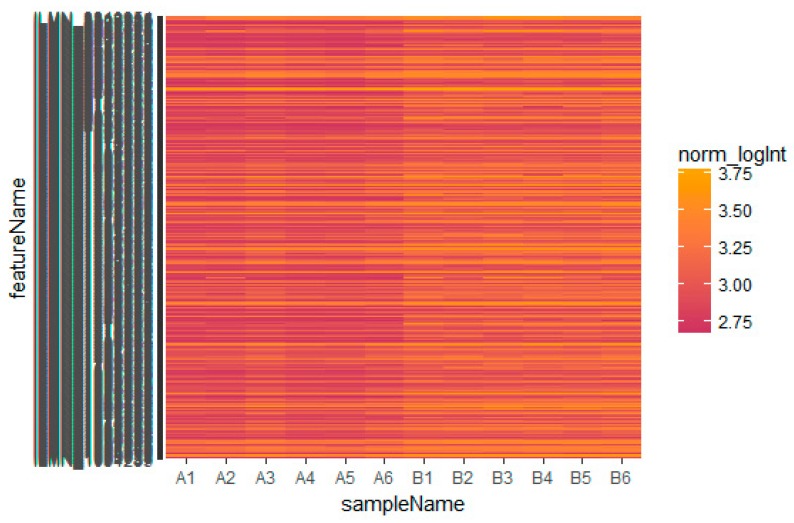
Heat map of down-regulated differentially expressed genes. The legend on the top left indicates the log fold change of genes. (A1, A2, A3, A4, A5, A6 = omental adipose tissue samples; B1, B2, B3, B4, B5, B6 = subcutaneous adipose tissue samples.)

Based on the defined criteria (log FC (fold change) > 1.24 for up-regulated genes and log FC (fold change) −0.725 for down-regulated genes) we compared the results for the two types of samples in the dataset in terms of the total differentially expressed genes using R Bioconductor. Among the total of 873 DEGs diagnosed in the GEO data sets were 435 up-regulated and 438 down-regulated DEGs (Table S1). A heatmap showed that the identified up-regulated and down-regulated DEGs could differentiate the omental adipose tissue samples with insulin resistance and the subcutaneous adipose tissue samples with insulin resistance (Figure 2 and Figure 3).

**Figure 4:**
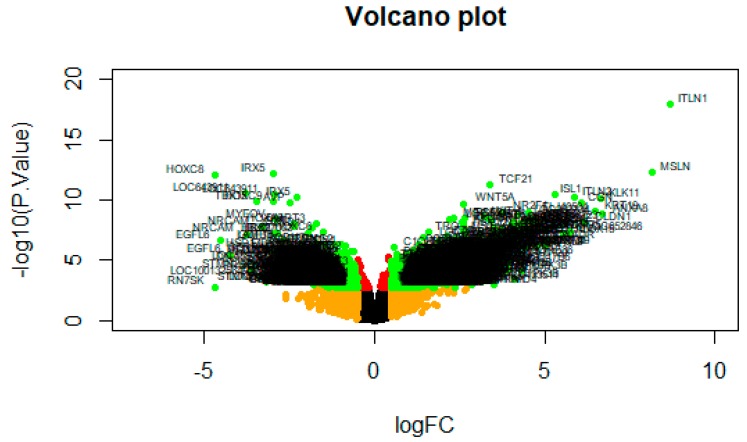
A volcano plot of differentially expressed genes. Genes with a significant change of more than 2-fold were selected.

A volcano plot of the DEG screening is shown in Figure 4.

### 3.2. Pathway Enrichment Analysis of Differentially Expressed Genes

To analyze the biological classification of the DEGs, a pathway enrichment analysis was performed using Enrichr. The pathway analysis results showed that up-regulated DEGs were significantly enriched in complement and coagulation cascades and cell adhesion molecules (CAMs) for KEGG; complement activation and complement and coagulation cascades for WikiPathways; the classical complement pathway and the lectin-induced complement pathway for BioCarta; regulation of the complement cascade and activation of C3 and C5 for Reactome; retinoate biosynthesis I and nicotine degradation IV for HumanCyc; the Wnt signaling network and the non-canonical Wnt signaling pathway for NCI-Nature; and the cadherin signaling pathway and the Alzheimer disease–presenilin pathway for Panther (Table S2). Down-regulated DEGs were significantly enriched in the biosynthesis of amino acids and the Notch signaling pathway for KEGG; the Notch signaling pathway and the Notch signaling pathway for WikiPathways; the segmentation clock and the p53 signaling pathway for BioCarta; metabolism of vitamins and cofactors and diseases of glycosylation for Reactome; glycine betaine degradation and gluconeogenesis for HumanCyc; the Notch signaling pathway and validated targets of C-MYC transcriptional repression for NCI-Nature; and the Notch signaling pathway and the nicotine pharmacodynamics pathway for Panther (Table S3).

### 3.3. Gene OntologyEnrichment Analyses of Differentially Expressed Genes

The enriched GO terms for DEGs were identified using the online tool Enrichr. The up-regulated genes were significantly enriched in the functions of cardiac muscle cell–cardiac muscle cell adhesion and regulation of transforming growth factor β2 production for BP, microvillus membrane and axonal growth cone for CC, and protein binding involved in heterotypic cell–cell adhesion and coreceptor activity involved in the Wnt signaling pathway and the planar cell polarity pathway for MF (Table S4). Down-regulated genes were significantly enriched in the functions of negative regulation of osteoblast differentiation and negative regulation of cell differentiation for BP, dendrite and intercalated disc for CC, and estradiol 17-β-dehydrogenase activity and steroid dehydrogenase activity acting on the CH–OH group of donors, Nicotinamide adenine dinucleotide (NAD) and Nicotinamide adenine dinucleotide phosphate (NADP)as acceptor for MF (Table S5).

### 3.4. Construction of the Protein–Protein InteractionNetwork and Topological Analysis

**Figure 5:**
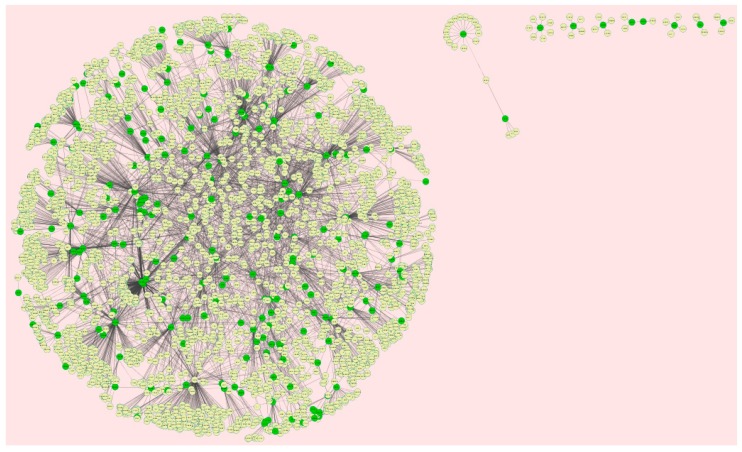
Protein–protein interaction network of differentially expressed genes (DEGs). Green nodes denote up-regulated genes.

Protein–protein interaction networks were constructed on the basis of STRING database. We analyzed the network properties such as node degree, betweenness centrality, stress centrality, closeness centrality, and cluster coefficient. The PPI network for up-regulated DEGs is shown in Figure 5; it has 3280 nodes and 5633 interactions.

**Figure 6:**
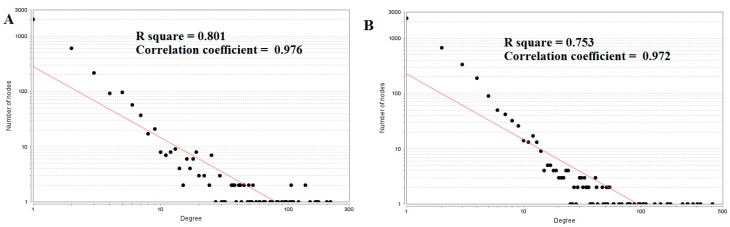
Node degree distribution: (**A**) Up-regulated genes; (**B**) Down-regulated genes.

The top five nodes with the greatest degrees are listed in Table S6, including MYO5B (degree = 214), DLG2 (degree = 200), ERBB3 (degree = 167), RHOU (degree = 163), and SNCA (degree = 148). The R-squared value and correlation coefficient were 0.801and 0.976, respectively (Figure 6).

**Figure 7:**
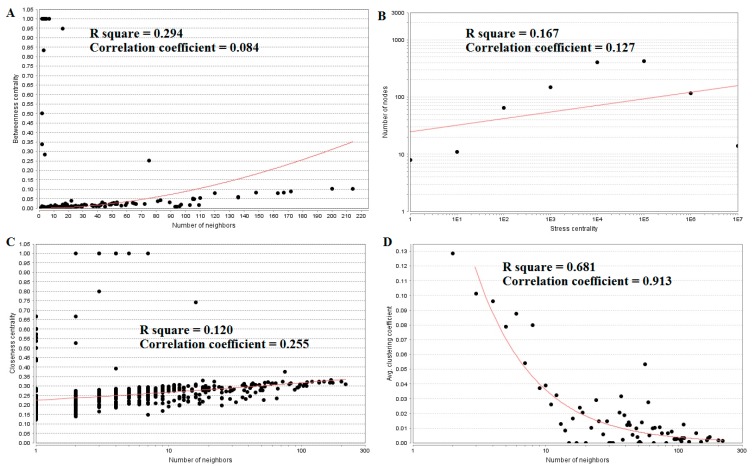
Regression diagrams for up-regulated genes: (**A**) Betweenness centrality; (**B**) Stress centrality; (**C**) Closeness centrality; (**D**) Clustering coefficient.

The top five up-regulated genes with high between ness centrality were SGPP2 (betweenness = 0.94736842), IGSF3 (betweenness = 0.83333333), VSIG2 (betweenness = 0.5), ITLN1 (betweenness = 0.28421053), and DLG2 (betweenness = 0.10398507), as shown in Table S6. The R-squared value and correlation coefficient were 0.294 and 0.084, respectively (Figure 7A).

The top five up-regulated high-stress genes were MYO5B (stress = 27380848), RHOU (stress = 20229932), ERBB3 (stress = 19629044), PRKCZ (stress = 18024744), and SNCA (stress = 13967348), as shown in Table S6. The R-squared value and correlation coefficient were 0.167 and 0.127, respectively (Figure 7B). The top five up-regulated genes with high closeness centrality were IGSF3 (closeness = 0.8), SGPP2 (closeness = 0.74074074), VSIG2 (closeness = 0.66666667), ITLN1 (closeness = 0.39215686), and ERBB3 (closeness = 0.33147919), as shown in Table S6. The R-squared value and correlation coefficient were 0.120 and 0.255, respectively (Figure 7C). The top five up-regulated genes with low clustering coefficient were CCL21 (clustering coefficient = 0), CYP27A1 (clustering coefficient = 0), RPRM (clustering coefficient = 0), BCHE (clustering coefficient = 0), and CPB1 (clustering coefficient = 0), as shown in Table S6. The R-squared value and correlation coefficient were 0.681 and 0.913, respectively (Figure 7D).

**Figure 8:**
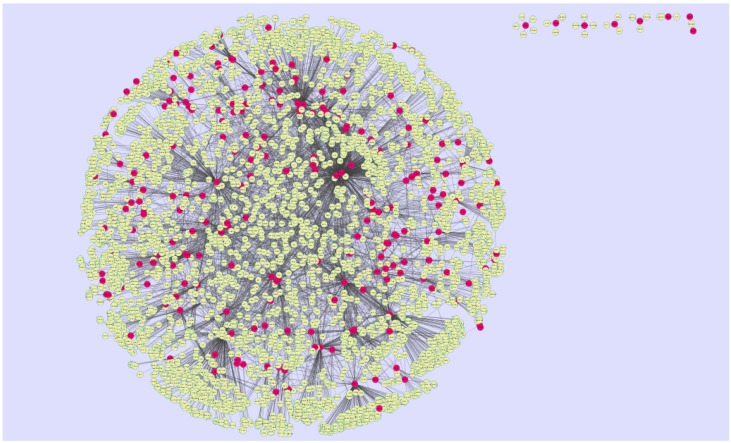
Protein–protein interaction network of DEGs. Red nodes denote down-regulated genes.

The PPI network for the down-regulated DEGs is shown in Figure 8; it has 3897 nodes and 7114 interactions.

**Figure 9:**
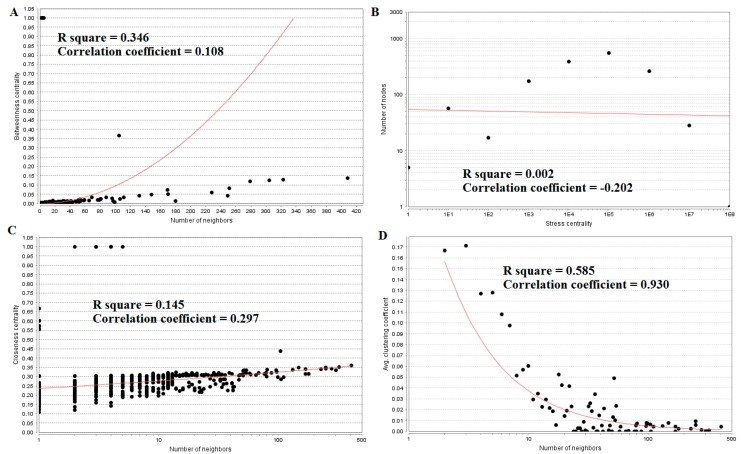
Regression diagrams for down-regulated genes: (**A**) Betweenness centrality; (**B**) Stress centrality; (**C**) Closeness centrality; (**D**) Clustering coefficient.

The top five nodes with the greatest degrees are listed in Table S6; they were NOTCH1 (degree = 408), AR (degree = 323), CPS1 (degree = 304), TUBB2A (degree = 279), and MRAS (degree = 251). The R-squared value and correlation coefficient were 0.753 and 0.972, respectively (Figure 6B). The top five down-regulated genes with high betweenness centrality were NOTCH1 (betweenness = 0.13655196), AR (betweenness = 0.1288082), CPS1 (betweenness = 0.125228), TUBB2A (betweenness = 0.12013939), and MRAS (betweenness = 0.08364569), as shown in Table S6. The R-squared value and correlation coefficient were 0.346 and 0.108, respectively (Figure 9A).

The top five down-regulated genes with high-stress genes were NOTCH1 (stress = 173740018), CPS1 (stress = 95361896), AR (stress = 79021038), NOTCH3 (stress = 65886422), and SOS1 (stress = 54135566), as shown in Table S6. The R-squared value and correlation coefficient were 0.002 and −0.202, respectively (Figure 9B). The top five down-regulated genes with high closeness centrality were NOTCH1 (closeness = 0.36067563), AR (closeness = 0.3522282), NOTCH3 (closeness = 0.34961556), CCND1 (closeness = 0.34866937), and MRAS (closeness = 0.34321996), as shown in Table S6. The R-squared value and correlation coefficient were 0.145 and 0.297, respectively (Figure 9C). The top five down-regulated genes with low clustering coefficient were SLC38A10 (clustering coefficient = 0), SYNC (clustering coefficient = 0), NMB (clustering coefficient = 0), POSTN (clustering coefficient = 0), and DMRT3 (clustering coefficient = 0), as shown in Table S6. The R-squared value and correlation coefficient were 0.585 and 0.930, respectively (Figure 9D).

### 3.5. Module Analysis

**Figure 10:**
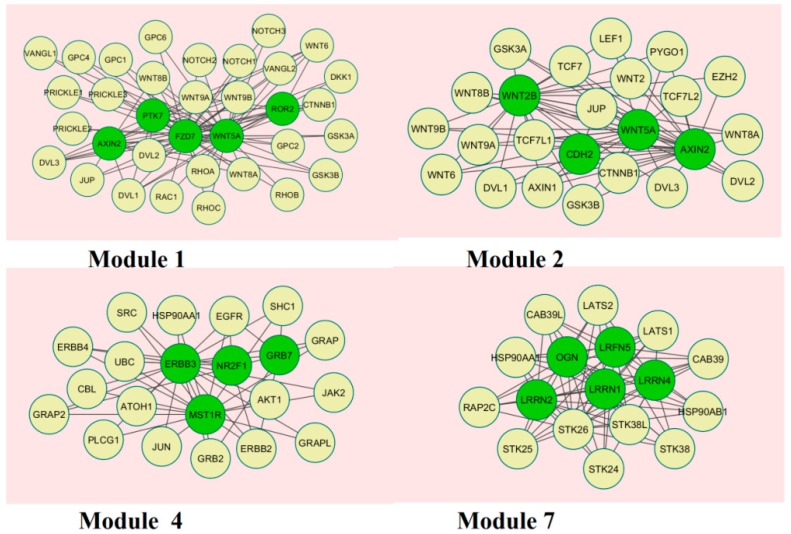
Modules in theprotein–protein interaction (PPI)network. The green nodes denote the up-regulated genes.

A total of 237 modules were identified in the up-regulated PPI network, among which the best were Module 1, Module 2, Module 4, and Module 7 (Figure 10).

Module 1 was composed of 34 nodes and 89 edges; the hub proteins in this module wereAXIN2 (degree = 59), PTK7 (degree = 72), FZD7 (degree = 56), WNT5A (degree = 136), and ROR2 (degree = 37). Module 2 was composed of 24 nodes and 65 edges; the hub proteins in this module wereWNT2B (degree = 109), CDH2 (degree = 64), WNT5A (degree = 136), and AXIN2 (degree = 59). Module 4 was composed of 21 nodes and 47 edges; the hub proteins in this module wereERBB3 (degree = 167), MST1R (degree = 53), NR2F1 (degree = 43), and GRB7 (degree = 25). Module 7 was composed of 17 nodes and 71 edges; the hub proteins in this module were LRRN2 (degree = 94), OGN (degree = 103), LRFN5 (degree = 93), LRRN1 (degree = 97), and LRRN4 (degree = 96).

**Figure 11:**
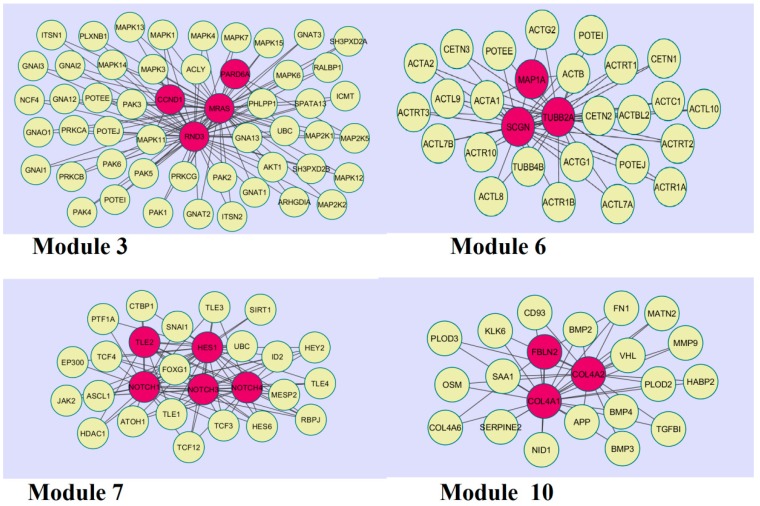
Modules in the PPI network. The red nodes denote the down-regulated genes.

A total of 466 modules are identified in the down-regulated PPI network, among which the best were Module 3, Module 6, Module 7, and Module 10 (Figure 11).

Module 3 was composed of 52 nodes and 106 edges; the hub proteins in this module were CCND1 (degree = 148), RND3 (degree = 170), MRAS (degree = 251), and PARD6A (degree = 81). Module 6 was composed of 28 nodes and 54 edges; the hub proteins in this module wereMAP1A (degree = 29), SCGN (degree = 111), and TUBB2A (degree = 279). Module 7 was composed of 27 nodes and 87 edges; the hub proteins in this module were TLE (degree = 54), NOTCH1 (degree = 408), NOTCH3 (degree = 249), NOTCH4 (degree = 180), and HES1 (degree = 52).Finally, Module 10 was composed of 22 nodes and 42 edges; the hub proteins in this module wereFBLN2 (degree = 20), COL4A1 (degree = 34), and COL4A2 (degree = 22).

### 3.6. Construction of the Target Gene–MicroRNA Network

**Figure 12:**
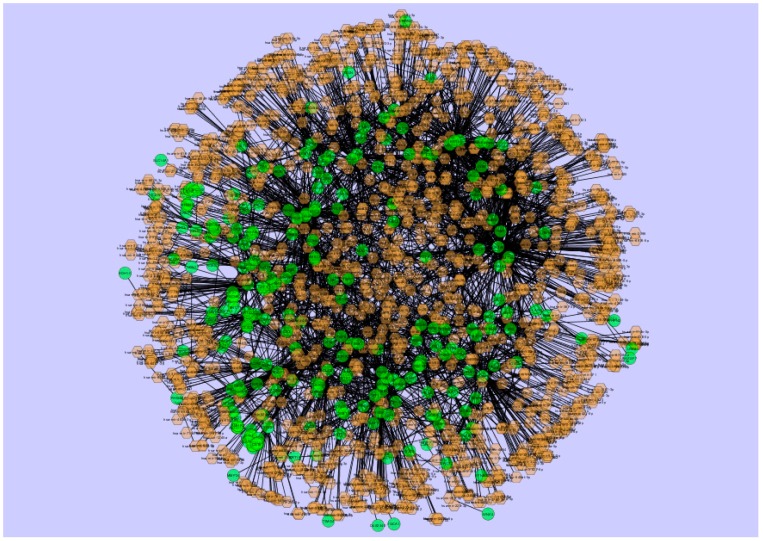
The network of up-regulated DEGs and their related miRNAs. The green circle nodes are the up-regulated DEGs and brown diamond nodes are the miRNAs.

**Figure 13:**
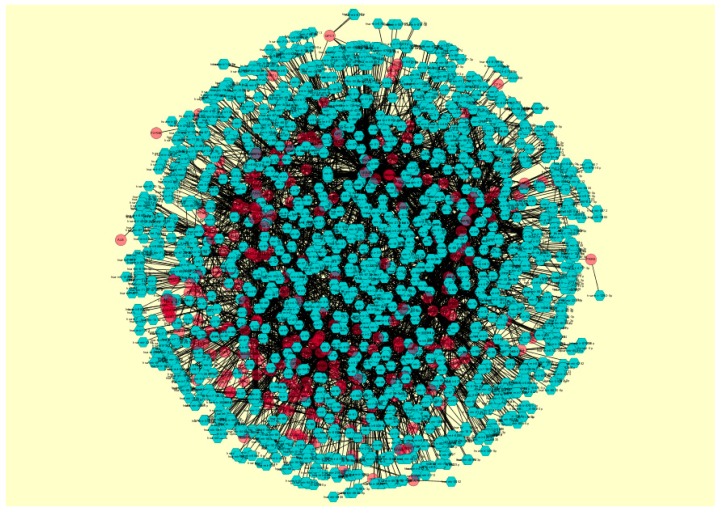
The network of down-regulated DEGs and their related miRNAs. The red circle nodes are the down-regulated DEGs and blue diamond nodes are the miRNAs.

The miRNAs that may control the DEGs were diagnosed based on the up- and down-regulation expressions (Figure 12 and Figure 13).

The top five up-regulated targeted genes wereGATA6 regulated by 202 miRNAs, ENPP5 regulated by 114 miRNAs, C3 regulated by 93 miRNAs, SLC38A1 regulated by 73 miRNAs, and TM4SF1 regulated by 68 miRNAs; these are given in Table S7. The top five down-regulated targeted genes were CCND1 regulated by 197 miRNAs, TUBB2A regulated by 183 miRNAs, CDKN1A regulated by 131 miRNAs, MYPN regulated by 120 miRNAs, and AR regulated by 96 miRNAs; these are given in Table S7.

### 3.7. Construction of the Target Gene–Transcription FactorNetwork

**Figure 14:**
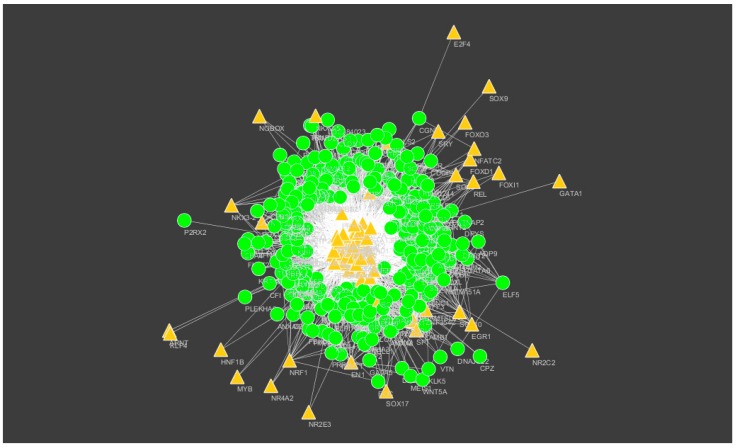
Transcription factors (TF)–target gene network of predicted target up-regulated genes. (Yellow triangles signify TFs and green circles signify target up-regulated genes).

**Figure 15:**
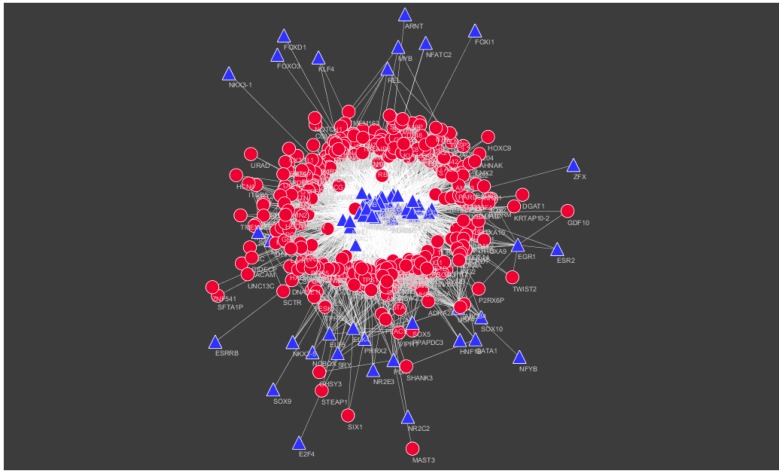
TF–target gene network of predicted target down-regulated genes. (Blue triangles signify TFs and red circles signify target down-regulated genes).

The TFs for target up- and down-regulated genes are shown in Figure 14 and Figure 15, respectively.

The top five up-regulated targeted genes wereTPD52L1 regulated by 182 TFs, OLFML1 regulated by 150 TFs, HPSE regulated by 90 TFs, CYP27A1 regulated by 79 TFs, and MUC1 regulated by 79 TFs; these are given in Table S8. The top five down-regulated targeted genes werePARM1 regulated by 181 TFs, ALDH4A1 regulated by 89 TFs, VENTX regulated by 79 TFs, KIAA0040 regulated by 75 TFs, and ADRA2A regulated by 74 TFs, and are given in Table S8.

## 4. Discussion

Obesity and NIDDM are among the highest-occurring universal health complications in the industrialized world. Obesity and NIDDM are metabolic disorders which arise due to an imbalance between caloric intake and energy expenditure and which are responsible for the development of insulin resistance [7,47]. An understanding of the molecular mechanism of insulin resistance is important. In the current study, a dataset (GSE73108) linked with insulin resistance was downloaded from the GEO database and analyzed. A total of 873 DEGs were diagnosed, including 435 up-regulated and 438 down-regulated DEGs. Tabassum et al. [48] stated that adipokine ITLN1 increases insulin-stimulated glucose uptake in adipocytes, and mutation in this gene is responsible for development of insulin resistance in NIDDM. Transcription factor protein ISL1 binds to the enhancer region of the insulin gene and activates the synthesis of insulin [49]. A single-nucleotide polymorphism (SNP) in ISL1 elevates body weight in NIDDM patents with insulin resistance [50]. Stimulation of non-canonical Wnt signaling through the overexpression of WNT5A, which is important for inflammation in adipose tissue, is associated with obesity [51] and NIDDM [52]. This gene may be responsible for the development of insulin resistance. Protease KLK7 degrades vaspinand is important for inflammation in adipose tissue, which is responsible for the development of obesity and insulin resistance [53]. Tong et al. [54] proved that SYT4 is responsible for the pathogenesis of obesity via the control of oxytocin release. This gene may be associated with insulin resistance. IRX5 is important for the advancement of obesity [55], but this gene may also link with insulin resistance. Decreased expression of TBX15 is responsible for increased fat deposition, leading to obesity [56], but this gene may also be involved in insulin resistance. HOXC9 expression is linked with loss of adipose tissue function and visceral fat distribution, resulting in obesity [57], but this gene may be important for the improvement of insulin resistance. arginine vasopressin (AVP) is associated with the development of obesity [58], but may be responsible for the improvement of insulin resistance. SNP in CCND1 is linked with obesity [59], but this polymorphic gene may diagnose insulin resistance.

In the present study, complement and coagulation cascades are the most significant KEGG pathway for up-regulated genes. A high level of C3 is associated with obesity and NIDDM, which are responsible for the development of insulin resistance [60]. A high level of vitronectin (VTN) is responsible for the advancement of NIDDM [61], but this gene may be associated with insulin resistance. Elevated expression of C7 is important for obesity progression [62], but this may also be responsible for the development of insulin resistance. protein C receptor, endothelial (EPCR) (PROCR), C1R, CFI, PLAT, C4BPB, and CFB are novel biomarkers for the development of insulin resistance. Complement activation is the most significant WikiPathways pathway for up-regulated genes. C2 is a novel biomarker for the development of insulin resistance. The classical complement pathway is the most significant BioCarta pathway for up-regulated genes, while regulation of the complement cascade is the most significant Reactome pathway for up-regulated genes. Retinoate biosynthesis I is the most significant HumanCyc pathway for up-regulated genes. A high level of ALDH1A3 is linked to β-cell dysfunction in NIDDM [63], but this gene may be responsible for the advancement of insulin resistance. ALDH1A2 and RBP1 are novel biomarkers for the development of insulin resistance. The Wnt signaling network is the most significant NCI-Nature pathway for up-regulated genes. A high level of RSPO1 is responsible for the development of obesity and insulin resistance [64]. WNT inhibitory factor 1 (WIF1), FZD7, and ROR2 are novel biomarkers for the development of insulin resistance. The cadherin signaling pathway is the most significant Panther pathway for up-regulated genes. WNT2B is a component of the Wnt signaling pathway, and this gene which activates the Wnt signaling pathway is responsible for the development of NIDDM [65] but may be identified with insulin resistance. Stimulation of p38 mitogen-activated protein kinase is important for transactivation of the ERBB3 receptor leading to IRS-1 Ser phosphorylation and insulin resistance [66]. Cadherin 3, type 1, P-cadherin (CDH3), CDH2, PCDH20, and CDH23 are novel biomarkers for the development of insulin resistance. Biosynthesis of amino acids is the most significant KEGG pathway for down-regulated genes. A low level of PC synthesis is responsible for the progression of hepatic insulin resistance and NIDDM [67].An SNP in CPS1 is linked with the development of NIDDM [68], but this polymorphic gene may be responsible for the advancement of insulin resistance. Low expression of PSAT1 contributes to the progression of insulin resistance through the inactivation of insulin signaling [69]. Serine hydroxymethyltransferase 1 (SHMT1), ALDOC, SDSL, ASS1, and IDH3A are novel biomarkers for the development of insulin resistance. The Notch signaling pathway is the most significant WikiPathways, NCI-Nature, and Panther pathway for down-regulated genes. Notch homolog 1 (NOTCH1) and NOTCH4 are components of the Notch signaling pathway, and activation of the Notch signaling pathway is important for the improvement of insulin resistance [70,71]. J 2 (JAG2), NOTCH3, CDKN1A, HES1, and MAPT are novel biomarkers for the development of insulin resistance. The Segmentation Clock is the most significant BioCarta pathway for down-regulated genes. Low expression of the WNT inhibitor DKK2 is responsible for fat accumulation in obesity [72], but this gene may be responsible for the improvement of insulin resistance. LFNG is a novel biomarker for the development of insulin resistance. Metabolism of vitamins and cofactors is the most significant Reactome pathway for down-regulated genes. Modification in CYB5A is associated with weight-regulating pathways in obesity and NIDDM [73], but this gene may diagnose insulin resistance. Aberrant expression of RBP4 is linked with development of obesity [74], NIDDM [75], and insulin resistance [76]. Abnormal expression of AKR1C3 is important for the development of obesity [77], NIDDM [78], and insulin resistance [79]. Vitamin K epoxide reductase complex, subunit 1-like 1 (VKORC1L1), BTD, GPC1, MOCOS, GPC5, AKR1C4, and NMNAT2 are novel biomarkers for the development of insulin resistance. Glycine betaine degradation is the most significant HumanCyc pathway for down-regulated genes, and BHMT is a novel biomarker for the development of insulin resistance.

In the current study, cardiac muscle cell–cardiac muscle cell adhesion is the most significant GO BP term for up-regulated genes. Desmoplakin (DSP), CXADR, and PKP2 are novel biomarkers for the development of insulin resistance. Microvillus membrane is the most significant GO CC term for up-regulated genes. Solute carrier family 9, member 3 regulator 1 (SLC9A3R1), PODXL, and PDPN are novel biomarkers for the development of insulin resistance. Protein binding involved in heterotypic cell–cell adhesion is the most significant GO MF term for up-regulated genes. Negative regulation of osteoblast differentiation is the most significant GO BP term for down-regulated genes. Loss of LRP5 together with Wnt proteins is responsible for the progression of obesity [80], NIDDM [81], and insulin resistance [82]. Low expression of TWIST2 is involved in obesity [83], but this gene may link with insulin resistance. Decreased expression of TWIST1 results in elevated secretion of TNFα, which is responsible for the progression of obesity and insulin resistance [84]. The GDF10 and TNN are novel biomarkers for the development of insulin resistance. Dendrite is the most significant GO CC term for down-regulated genes. Low expression of SCGN is responsible for the progression of insulin resistance. Loss of co-expression of both SCGN and TAU is linked with insulin resistance [85]. Loss of clusterin (CLU) is associated with NIDDM [86] and insulin resistance [87]. Corticotropin releasing hormone binding protein (CRHBP), ARHGEF15, MME, MAP1A, FGF13, and SHANK3 are novel biomarkers for the development of insulin resistance. Estradiol 17-beta-dehydrogenase activity is the most significant GO MF term for down-regulated genes. Dehydrogenase/reductase member 11 (DHRS11) and AKR1B15 are novel biomarkers for the development of insulin resistance.

In the present study, MYO5B, DLG2, ERBB3, RHOU, and SNCA were identified as hub proteins (up-regulated DEGs) in the PPI network. MYO5B, DLG2, RHOU, and SNCA are novel biomarkers for the development of insulin resistance. SGPP2, IGSF3, VSIG2, ITLN1, and DLG2 are the hub proteins (up-regulated DEGs) with the highest betweenness centrality in the PPI network. SGPP2, IGSF3, and VSIG2 are novel biomarkers for the development of insulin resistance. myosin VB (MYO5B), RHOU, ERBB3, PRKCZ, and SNCA are the hub proteins (up-regulated DEGs) with the highest stress centrality in the PPI network. Protein kinase C/zeta (PRKCZ) is a novel biomarker for the development of insulin resistance. IGSF3, SGPP2, VSIG2, ITLN1, and ERBB3 are the hub proteins (up-regulated DEGs) with the highest closeness centrality in the PPI network. Chemokine (C-C motif) ligand 21 (CCL21), CYP27A1, RPRM, BCHE, and CPB1 are the hub proteins (up-regulated DEGs) with the lowest clustering coefficient in the PPI network. A high level of BCHE is responsible for the development of insulin resistance [88]. Chemokine (C-C motif) ligand 21 (CCL21), CYP27A1, RPRM, and CPB1 are novel biomarkers for the development of insulin resistance. Notch homolog 1 (NOTCH1), AR, CPS1, TUBB2A, and MRAS were identified as hub proteins (down-regulated DEGs) in the PPI network. Low expression of AR is linked with elevated insulin resistance on a high-fat diet with decreased β-oxidation of fatty acids [89]. The TUBB2A and MRAS are novel biomarkers for the development of insulin resistance. Notch homolog 1 (NOTCH1), AR, CPS1, TUBB2A, and MRAS are the hub proteins (down-regulated DEGs) with the highest betweenness centrality in the PPI network. The NOTCH1, CPS1, AR, NOTCH3, and SOS1 are the hub proteins (down-regulated DEGs) with the highest stress centrality in the PPI network. Polymorphism in SOS1 is responsible for the progression of gestational diabetes mellitus [90], but this polymorphic gene may link with insulin resistance and NIDDM. The NOTCH1, AR, NOTCH3, CCND1, and MRAS are the hub proteins (down-regulated DEGs) withthe highest closeness centrality in the PPI network. Solute carrier family 38, member 10 (SLC38A10), SYNC, NMB, POSTN, and DMRT3 are the hub proteins (down-regulated DEGs) with the lowest clustering coefficient in the PPI network. Polymorphism in NMB is associated with obesity [91], but this gene may link with insulin resistance. An elevated level of POSTN is important for the improvement of obesity and NIDDM [92], but this gene may be involved in insulin resistance. Solute carrier family 38, member 10 (SLC38A10), SYNC, and DMRT3 are novel biomarkers for the development of insulin resistance.

Modules were extracted from the PPI network for up- and down-regulated DEGs. Axin 2 (AXIN2), PTK7, FZD7, WNT5A, ROR2, WNT2B, CDH2, ERBB3, MST1R, NR2F1, GRB7, LRRN2, OGN, LRFN5, LRRN1, and LRRN4 are the hub proteins (up-regulated DEGs with high degree) in all four modules in the PPI network. Axin 2 (AXIN2), PTK7, MST1R, NR2F1, GRB7, LRRN2, OGN, LRFN5, LRRN1, and LRRN4 are novel biomarkers for the development of insulin resistance. Cyclin D1 (CCND1), RND3, MRAS, PARD6A, MAP1A, SCGN, TUBB2A, TLE2, NOTCH1, NOTCH3, NOTCH4, HES1, FBLN2, COL4A1, and COL4A2 are the hub proteins (down-regulated DEGs with high degree) in all four modules in the PPI network. Rho family GTPase 3 (RND3), PARD6A, TLE2, FBLN2, COL4A1, and COL4A2 are novel biomarkers for the development of insulin resistance.

GATA binding protein 6 (GATA6), ENPP5, C3, SLC38A1, and TM4SF1 were identified as up-regulated target genes (high degree of connectivity with miRNAs) in the miRNA–target gene regulatory network. Alteration in GATA6 is responsible for the development of NIDDM [93], but this gene may be identified with insulin resistance. Ectonucleotide pyrophosphatase/phosphodiesterase 5 (ENPP5), SLC38A1, and TM4SF1 are novel biomarkers for the development of insulin resistance. Cyclin D1 (CCND1), TUBB2A, CDKN1A, MYPN, and AR were identified as down-regulated target genes (high degree of connectivity with miRNAs) in the miRNA–target gene regulatory network. MYPN is novel biomarker for the development of insulin resistance.

Tumor protein D52-like 1 (TPD52L1), OLFML1, HPSE, CYP27A1, and MUC1 were identified as up-regulated target genes (high degree of connectivity with TFs) in the TF–target gene regulatory network. Increased expression of HPSE is responsible for islet β cell autoimmunity in diabetes [94], but this gene may be associated with insulin resistance. Tumor protein D52-like 1 (TPD52L1), OLFML1, and MUC1 are novel biomarkers for the development of insulin resistance. Prostate androgen-regulated mucin-like protein 1 (PARM1), ALDH4A1, VENTX, KIAA0040, and ADRA2A were identified as down-regulated target genes (high degree of connectivity with TFs) in the TF–target gene regulatory network. Polymorphism in ADRA2A is responsible for the development of NIDDM [95] and obesity [96], but this gene may be identified with insulin resistance. Prostate androgen-regulated mucin-like protein 1 (PARM1), ALDH4A1, VENTX, and KIAA0040 are novel biomarkers for the development of insulin resistance.

## 5. Conclusions

We aimed to diagnose DEGs using bioinformatics analysis to find molecular mechanisms associated with the development of insulin resistance. The study provided four useful DEGs for future research into the molecular mechanisms of insulin resistance development. However, further functional examinations are required to analyze the actions of these DEGs in the development of insulin resistance.

## Supplementary Materials

The following are available online at http://www.mdpi.com/2218-273X/9/2/37/s1. Tables file provide in supplementary materials.
