# Supplementary material for: Integrative Analyses of Genes Associated with Subcutaneous Insulin Resistance
Source: Biomolecules. 2019 Jan 22;9(2):37. doi: 10.3390/biom9020037 (PMC6406848; doi:10.3390/biom9020037)
Supplement: Supplementary file 1 [file biomolecules-09-00037-s001.pdf]

Table S1 The statistical metrics for key differentially expressed genes (DEGs)

Illumina Id	Gene Symbol	logFC	pValue	FDR	tvalue	Regulation	Gene Name
ILMN_2165993	ITLN1	8.684117	1.07E-18	5.04E-14	95.96989	Up	"Homo sapiens intelectin 1 (galactofuranose binding) (ITLN1), mRNA."
ILMN_2353161	MSLN	8.140032	4.91E-13	9.75E-09	32.24662	Up	"Homo sapiens mesothelin (MSLN), transcript variant 2, mRNA."
ILMN_1728570	TCF21	3.385002	5.64E-12	5.33E-08	26.24676	Up	"Homo sapiens transcription factor 21 (TCF21), transcript variant 1, mRNA."
ILMN_1763390	ISL1	5.296494	3.41E-11	2.01E-07	22.53207	Up	"Homo sapiens ISL1 transcription factor, LIM/homeodomain, (islet-1) (ISL1), mRNA."
ILMN_2118129	ITLN2	5.871462	6.25E-11	2.96E-07	21.39665	Up	"Homo sapiens intelectin 2 (ITLN2), mRNA."
ILMN_1695924	KLK11	6.628375	8.21E-11	3.53E-07	20.90392	Up	"Homo sapiens kallikrein-related peptidase 11 (KLK11), transcript variant 2, mRNA."
ILMN_1746801	CGN	6.052765	1.72E-10	5.44E-07	19.61811	Up	"Homo sapiens cingulin (CGN), mRNA."
ILMN_1800317	WNT5A	2.625075	2.01E-10	5.95E-07	19.36083	Up	"Homo sapiens wingless-type MMTV integration site family, member 5A (WNT5A), mRNA."
ILMN_1730777	KRT19	6.461745	8.39E-10	2.34E-06	17.1213	Up	"Homo sapiens keratin 19 (KRT19), mRNA."
ILMN_1786197	NR2F1	3.751858	1E-09	2.64E-06	16.86022	Up	"Homo sapiens nuclear receptor subfamily 2, group F, member 1 (NR2F1), mRNA."
ILMN_2041222	KRT18P55	4.531194	1.15E-09	2.87E-06	16.65888	Up	"Homo sapiens keratin 18 pseudogene 55,mRNA."
ILMN_1778087	ANXA8	6.689182	1.54E-09	3.63E-06	16.24877	Up	"Homo sapiens annexin A8 (ANXA8), mRNA."
ILMN_1798557	LOC202134	4.206992	2.11E-09	4.76E-06	15.80531	Up	"PREDICTED: Homo sapiens hypothetical protein LOC202134, transcript variant 1 (LOC202134), mRNA."
ILMN_1723910	WIT-1	3.207408	2.3E-09	4.94E-06	15.68871	Up	"Homo sapiens Wilms tumor associated protein (WIT-1), mRNA."
ILMN_1705685	MEIS1	2.346418	3.61E-09	7.18E-06	15.08337	Up	"Homo sapiens Meis homeobox 1 (MEIS1), mRNA."
ILMN_1766712	TCF21	2.654083	3.64E-09	7.18E-06	15.07183	Up	"Homo sapiens transcription factor 21 (TCF21), transcript variant 2, mRNA."
ILMN_3237981	ATPGD1	4.593669	4.04E-09	7.34E-06	14.93425	Up	"Homo sapiens ATP-grasp domain containing 1 (ATPGD1), transcript variant 2, mRNA."
ILMN_1805665	FLRT3	4.658149	4.06E-09	7.34E-06	14.92991	Up	"Homo sapiens fibronectin leucine rich transmembrane protein 3 (FLRT3), transcript variant 2, mRNA."
ILMN_1697733	CST6	3.402206	4.47E-09	7.55E-06	14.80516	Up	"PREDICTED: Homo sapiens cystatin E/M (CST6), mRNA."
ILMN_3280842	KRT8P45	2.197599	4.63E-09	7.55E-06	14.75973	Up	"Homo sapiens ATP-grasp domain containing 1 (ATPGD1), transcript variant 2, mRNA."
ILMN_3237912	WIT1	3.43109	5.17E-09	8.15E-06	14.61739	Up	"Homo sapiens Wilms tumor upstream neighbor 1 (WIT1), non-coding RNA."
ILMN_1665425	RPRM	4.458764	6.15E-09	9.26E-06	14.39638	Up	"Homo sapiens reprimo, TP53 dependent G2 arrest mediator candidate (RPRM), mRNA."
ILMN_1694778	LOC646723	4.448603	6.26E-09	9.26E-06	14.37382	Up	"PREDICTED: Homo sapiens similar to Keratin, type I cytoskeletal 18 (Cytokeratin-18) (CK-18) (Keratin-18) (K18) (LOC646723), mRNA."
ILMN_1724686	CLDN1	6.172477	6.85E-09	9.79E-06	14.26007	Up	"Homo sapiens claudin 1 (CLDN1), mRNA."
ILMN_1671600	EPS8L1	2.588304	7.03E-09	9.79E-06	14.2273	Up	"Homo sapiens EPS8-like 1 (EPS8L1), transcript variant 1, mRNA."
ILMN_1743620	RARRES1	4.091466	8.01E-09	1.08E-05	14.0659	Up	"Homo sapiens retinoic acid receptor responder (tazarotene induced) 1 (RARRES1), transcript variant 2, mRNA."
ILMN_1725746	ATPGD1	4.048623	8.65E-09	1.14E-05	13.97123	Up	"Homo sapiens ATP-grasp domain containing 1 (ATPGD1), transcript variant 2, mRNA."
ILMN_1772627	D4S234E	4.7017	9.63E-09	1.16E-05	13.83922	Up	"Homo sapiens DNA segment on chromosome 4 (unique) 234 expressed sequence (D4S234E), transcript variant 2, mRNA."
ILMN_1745570	KLK7	4.262127	9.86E-09	1.16E-05	13.81075	Up	"Homo sapiens kallikrein-related peptidase 7 (KLK7), transcript variant 1, mRNA."
ILMN_1811437	C11ORF9	2.59692	1.01E-08	1.16E-05	13.78681	Up	"Homo sapiens chromosome 11 open reading frame 9 (C11orf9), transcript variant 1, mRNA."
ILMN_2189371	NTNG1	4.08723	1.19E-08	1.34E-05	13.58432	Up	"Homo sapiens netrin G1 (NTNG1), transcript variant 3, mRNA."
ILMN_1800091	RARRES1	4.210778	1.32E-08	1.46E-05	13.4576	Up	"Homo sapiens retinoic acid receptor responder (tazarotene induced) 1 (RARRES1), transcript variant 1, mRNA."

ILMN_1768705	SYT4	4.099696	1.54E-08	1.62E-05	13.27659	Up	"Homo sapiens synaptotagmin IV (SYT4), mRNA."
ILMN_1803211	FBXO2	2.608996	1.96E-08	1.98E-05	12.99529	Up	"Homo sapiens F-box protein 2 (FBXO2), mRNA."
ILMN_1727778	NTNG1	3.498184	2.09E-08	2.02E-05	12.92034	Up	"Homo sapiens netrin G1 (NTNG1), transcript variant 3, mRNA."
ILMN_1671971	LOC644743	4.164172	2.21E-08	2.09E-05	12.85847	Up	"PREDICTED: Homo sapiens hypothetical LOC644743 (LOC644743), mRNA."
ILMN_2125346	MUC16	4.40295	2.29E-08	2.12E-05	12.82082	Up	"Homo sapiens mucin 16, cell surface associated (MUC16), mRNA."
ILMN_1653687	GALNT9	4.067043	2.47E-08	2.25E-05	12.73292	Up	"Homo sapiens UDP-N-acetyl-alpha-D-galactosamine:polypeptide N-acetylgalactosaminyltransferase 9 (GalNAc-T9) (GALNT9), mRNA."
ILMN_3263974	KRT18P13	3.75781	2.75E-08	2.45E-05	12.61337	Up	"PREDICTED: Homo sapiens keratin 18 pseudogene 13 (KRT18P13), mRNA."
ILMN_1764885	C20ORF75	4.580189	3.18E-08	2.75E-05	12.45071	Up	"Homo sapiens chromosome 20 open reading frame 75 (C20orf75), mRNA."
ILMN_1751465	BNC1	5.02406	3.19E-08	2.75E-05	12.44511	Up	"Homo sapiens basonuclin 1 (BNC1), mRNA."
ILMN_1802174	WT1	4.438531	3.25E-08	2.75E-05	12.42452	Up	"Homo sapiens Wilms tumor 1 (WT1), transcript variant D, mRNA."
ILMN_3236160	C4ORF49	4.589	3.51E-08	2.92E-05	12.3389	Up	"Homo sapiens chromosome 4 open reading frame 49 (C4orf49), mRNA."
ILMN_1799098	LOC652846	5.740792	4.58E-08	3.67E-05	12.04923	Up	"PREDICTED: Homo sapiens similar to Annexin A8 (Annexin VIII) (Vascular anticoagulant-beta) (VAC-beta) (LOC652846), mRNA."
ILMN_2409642	TRO	1.582639	4.75E-08	3.74E-05	12.01108	Up	"Homo sapiens trophinin (TRO), transcript variant 6, mRNA."
ILMN_1707464	MST1	2.606618	5.25E-08	4.07E-05	11.90379	Up	"Homo sapiens macrophage stimulating 1 (hepatocyte growth factor-like) (MST1), mRNA."
ILMN_1683939	VIPR2	3.511691	5.72E-08	4.3E-05	11.81124	Up	"Homo sapiens vasoactive intestinal peptide receptor 2 (VIPR2), mRNA."
ILMN_1806667	FRAS1	4.386564	5.92E-08	4.38E-05	11.77493	Up	"Homo sapiens Fraser syndrome 1 (FRAS1), mRNA."
ILMN_2353054	KLK5	4.148017	6.8E-08	4.8E-05	11.62914	Up	"Homo sapiens kallikrein-related peptidase 5 (KLK5), transcript variant 1, mRNA."
ILMN_2298301	BNC1	4.474755	7.28E-08	4.97E-05	11.55859	Up	"Homo sapiens basonuclin 1 (BNC1), mRNA."
ILMN_1676042	ALOX15	5.54937	7.35E-08	4.97E-05	11.5474	Up	"PREDICTED: Homo sapiens arachidonate 15-lipoxygenase (ALOX15), mRNA."
ILMN_2176592	BCHE	4.329322	7.59E-08	5.06E-05	11.5147	Up	"Homo sapiens butyrylcholinesterase (BCHE), mRNA."
ILMN_1661628	LOC653110	4.073945	7.81E-08	5.13E-05	11.48473	Up	"PREDICTED: Homo sapiens similar to annexin A8, transcript variant 1 (LOC653110), mRNA."
ILMN_1694426	ROR2	3.107025	8.29E-08	5.38E-05	11.42266	Up	"Homo sapiens receptor tyrosine kinase-like orphan receptor 2 (ROR2), mRNA."
ILMN_2181892	BEX2	3.438705	9.35E-08	5.97E-05	11.29942	Up	"Homo sapiens brain expressed X-linked 2 (BEX2), mRNA."
ILMN_1685641	BCHE	4.212703	9.46E-08	5.97E-05	11.28776	Up	"Homo sapiens butyrylcholinesterase (BCHE), mRNA."
ILMN_3272378	EZR	2.140432	9.75E-08	6.07E-05	11.25682	Up	"Homo sapiens ezrin (EZR), transcript variant 1, mRNA."
ILMN_1682937	RSPO1	4.013119	1.01E-07	6.18E-05	11.21985	Up	"Homo sapiens R-spondin homolog (Xenopus laevis) (RSPO1), mRNA."
ILMN_1811364	SGPP2	3.741849	1.02E-07	6.18E-05	11.21146	Up	"PREDICTED: Homo sapiens sphingosine-1-phosphate phosphotase 2 (SGPP2), mRNA."
ILMN_1793025	OSAP	4.181253	1.03E-07	6.19E-05	11.19683	Up	"Homo sapiens ovary-specific acidic protein (OSAP), mRNA."
ILMN_1753584	KRT8	3.555651	1.15E-07	6.82E-05	11.08744	Up	"Homo sapiens keratin 8 (KRT8), mRNA."
ILMN_2285404	DMKN	3.075001	1.29E-07	7.53E-05	10.97468	Up	"Homo sapiens dermokine (DMKN), transcript variant 2, mRNA."
ILMN_3277209	LOC285943	1.426717	1.44E-07	8.28E-05	10.86649	Up	"PREDICTED: Homo sapiens hypothetical protein LOC285943 (LOC285943), miscRNA."
ILMN_1693941	IGSF9	2.414908	1.46E-07	8.28E-05	10.84897	Up	"Homo sapiens immunoglobulin superfamily, member 9 (IGSF9), mRNA."
ILMN_2052373	RAB17	2.70869	1.47E-07	8.28E-05	10.84536	Up	"Homo sapiens RAB17, member RAS oncogene family (RAB17), mRNA."
ILMN_1796423	CLIC3	3.628715	1.49E-07	8.31E-05	10.83001	Up	"Homo sapiens chloride intracellular channel 3 (CLIC3), mRNA."
ILMN_1687848	C7	3.407321	1.59E-07	8.76E-05	10.76669	Up	"Homo sapiens complement component 7 (C7), mRNA."

ILMN_2366967	WT1	4.232364	1.63E-07	8.86E-05	10.74367	Up	"Homo sapiens Wilms tumor 1 (WT1), transcript variant A, mRNA."
ILMN_2065773	SCG5	3.318088	2E-07	0.000107	10.54235	Up	"Homo sapiens secretogranin V (7B2 protein) (SCG5), mRNA."
ILMN_1765363	DSC3	3.494685	2.01E-07	0.000107	10.53932	Up	"Homo sapiens desmocollin 3 (DSC3), transcript variant Dsc3b, mRNA."
ILMN_1698666	CST6	3.7017	2.38E-07	0.000121	10.37708	Up	"Homo sapiens cystatin E/M (CST6), mRNA."
ILMN_1765310	TCEAL2	4.132552	2.38E-07	0.000121	10.3763	Up	"Homo sapiens transcription elongation factor A (SII)-like 2 (TCEAL2), mRNA."
ILMN_1796925	CXADR	4.998577	2.5E-07	0.000124	10.33152	Up	"Homo sapiens coxsackie virus and adenovirus receptor (CXADR), mRNA."
ILMN_1723678	PRPH	2.599298	2.51E-07	0.000124	10.32648	Up	"Homo sapiens peripherin (PRPH), mRNA."
ILMN_1725417	NELL2	3.522566	2.63E-07	0.000128	10.28203	Up	"Homo sapiens NEL-like 2 (chicken) (NELL2), mRNA."
ILMN_1795937	VIL2	2.388371	2.67E-07	0.000129	10.26731	Up	"Homo sapiens villin 2 (ezrin) (VIL2), mRNA."
ILMN_1715476	LOC649970	2.985285	2.71E-07	0.00013	10.25338	Up	"PREDICTED: Homo sapiens similar to creatine kinase, mitochondrial 1B precursor (LOC649970), mRNA."
ILMN_1721770	PAPPA	1.86056	2.83E-07	0.000134	10.21329	Up	"Homo sapiens pregnancy-associated plasma protein A, pappalysin 1 (PAPPA), mRNA."
ILMN_1814369	C21ORF62	3.500926	3.44E-07	0.000161	10.0315	Up	"Homo sapiens chromosome 21 open reading frame 62 (C21orf62), mRNA."
ILMN_1782004	BAPX1	3.604661	3.49E-07	0.000162	10.01738	Up	"Homo sapiens bagpipe homeobox homolog 1 (Drosophila) (BAPX1), mRNA."
ILMN_1754570	KCTD8	2.541146	3.85E-07	0.000175	9.928107	Up	"Homo sapiens potassium channel tetramerisation domain containing 8 (KCTD8), mRNA."
ILMN_1748538	ALDH1A2	2.888869	4E-07	0.00018	9.891117	Up	"Homo sapiens aldehyde dehydrogenase 1 family, member A2 (ALDH1A2), transcript variant 3, mRNA."
ILMN_1803956	BOC	1.295234	4.53E-07	0.000201	9.778172	Up	"Homo sapiens Boc homolog (mouse) (BOC), mRNA."
ILMN_1887267	HS.177532	2.256587	4.55E-07	0.000201	9.774308	Up	"BX355045 Homo sapiens NEUROBLASTOMA COT 25-NORMALIZED Homo sapiens cDNA clone CS0DC029YI23 5-PRIME, mRNA sequence"
ILMN_2072101	C4ORF49	4.480501	5.99E-07	0.000253	9.527038	Up	"Homo sapiens chromosome 4 open reading frame 49 (C4orf49), mRNA."
ILMN_2114720	SLPI	5.020655	6.1E-07	0.000254	9.511623	Up	"Homo sapiens secretory leukocyte peptidase inhibitor (SLPI), mRNA."
ILMN_1658709	LAMB1	1.517162	6.13E-07	0.000254	9.507156	Up	"Homo sapiens laminin, beta 1 (LAMB1), mRNA."
ILMN_2095610	ANXA8	4.77946	6.3E-07	0.000259	9.481614	Up	"Homo sapiens annexin A8 (ANXA8), mRNA. XM_931361 XM_931369 XM_931374 XM_931375 XM_931378 XM_931383 XM_931388 XM_931391 XM_931399 XM_931404 XM_931411"
ILMN_1670490	PDPN	3.810324	6.47E-07	0.00026	9.458303	Up	"Homo sapiens podoplanin (PDPN), transcript variant 4, mRNA."
ILMN_2413779	SEZ6L2	2.564288	6.69E-07	0.000264	9.428613	Up	"Homo sapiens seizure related 6 homolog (mouse)-like 2 (SEZ6L2), transcript variant 2, mRNA."
ILMN_1717793	C19ORF33	4.115413	6.72E-07	0.000264	9.424962	Up	"Homo sapiens chromosome 19 open reading frame 33 (C19orf33), mRNA."
ILMN_1784294	CPA4	4.097719	6.78E-07	0.000264	9.417577	Up	"Homo sapiens carboxypeptidase A4 (CPA4), mRNA."
ILMN_1758731	CYP2J2	3.024802	6.82E-07	0.000264	9.412432	Up	"Homo sapiens cytochrome P450, family 2, subfamily J, polypeptide 2 (CYP2J2), mRNA."
ILMN_1743219	CA11	3.035765	6.91E-07	0.000266	9.400387	Up	"Homo sapiens carbonic anhydrase XI (CA11), mRNA."
ILMN_2295518	TRO	1.407565	7.9E-07	0.0003	9.282908	Up	"Homo sapiens trophinin (TRO), transcript variant 6, mRNA."
ILMN_1910085	HS.536734	3.367488	7.92E-07	0.0003	9.280907	Up	"ab76b04.s1 Stratagene fetal retina 937202 Homo sapiens cDNA clone IMAGE:852847 3, mRNA sequence"
ILMN_1805765	CMYA5	2.289902	8.01E-07	0.000301	9.271455	Up	"Homo sapiens cardiomyopathy associated 5 (CMYA5), mRNA."
ILMN_1670145	DFNA5	1.988839	8.29E-07	0.000309	9.241389	Up	"Homo sapiens deafness, autosomal dominant 5 (DFNA5), transcript variant 1, mRNA."
ILMN_1781149	INMT	2.049627	8.96E-07	0.000327	9.174389	Up	"Homo sapiens indolethylamine N-methyltransferase (INMT), mRNA."
ILMN_1813753	PTN	1.729449	9.02E-07	0.000327	9.168334	Up	"Homo sapiens pleiotrophin (PTN), mRNA."
ILMN_1783443	ALOX15	3.929072	9.05E-07	0.000327	9.165716	Up	"PREDICTED: Homo sapiens arachidonate 15-lipoxygenase (ALOX15), mRNA."

ILMN_1733983	LOC653316	3.03306	9.51E-07	0.00034	9.122609	Up	"PREDICTED: Homo sapiens similar to NY-REN-7 antigen, transcript variant 4 (LOC653316), mRNA."
ILMN_2186983	ANXA8L2	4.372934	9.56E-07	0.00034	9.118264	Up	"Homo sapiens annexin A8-like 2 (ANXA8L2), mRNA."
ILMN_1739325	LOC284023	1.833939	9.71E-07	0.000343	9.104609	Up	"PREDICTED: Homo sapiens hypothetical protein LOC284023, transcript variant 3 (LOC284023), mRNA."
ILMN_1756992	MUC1	2.020251	9.88E-07	0.000346	9.090056	Up	"Homo sapiens mucin 1, cell surface associated (MUC1), transcript variant 6, mRNA."
ILMN_1727815	CFI	2.360177	1.06E-06	0.000369	9.028633	Up	"Homo sapiens complement factor I (CFI), mRNA."
ILMN_1676159	MST4	2.058419	1.07E-06	0.00037	9.02088	Up	"Homo sapiens serine/threonine protein kinase MST4 (MST4), transcript variant 1, mRNA."
ILMN_2161577	CXCL6	2.878104	1.08E-06	0.00037	9.013643	Up	"Homo sapiens chemokine (C-X-C motif) ligand 6 (granulocyte chemotactic protein 2) (CXCL6), mRNA."
ILMN_1813361	ANGPTL7	2.886197	1.09E-06	0.00037	9.008491	Up	"Homo sapiens angiopoietin-like 7 (ANGPTL7), mRNA."
ILMN_1732066	CKMT1A	2.961308	1.13E-06	0.000382	8.974911	Up	"Homo sapiens creatine kinase, mitochondrial 1A (CKMT1A), nuclear gene encoding mitochondrial protein, mRNA."
ILMN_1790778	PNMA2	2.05003	1.14E-06	0.000383	8.96596	Up	"Homo sapiens paraneoplastic antigen MA2 (PNMA2), mRNA."
ILMN_1677851	RARRES1	1.716003	1.18E-06	0.000389	8.938952	Up	"Homo sapiens retinoic acid receptor responder (tazarotene induced) 1 (RARRES1), transcript variant 2, mRNA."
ILMN_1669114	WNK4	1.599267	1.18E-06	0.000389	8.935872	Up	"Homo sapiens WNK lysine deficient protein kinase 4 (WNK4), mRNA."
ILMN_2383707	ALDH1A2	2.927523	1.24E-06	0.000405	8.895787	Up	"Homo sapiens aldehyde dehydrogenase 1 family, member A2 (ALDH1A2), transcript variant 2, mRNA."
ILMN_1808824	NEBL	1.935725	1.35E-06	0.000436	8.82272	Up	"Homo sapiens nebulette (NEBL), transcript variant 1, mRNA."
ILMN_2367010	GPR126	1.970507	1.37E-06	0.000437	8.815295	Up	"Homo sapiens G protein-coupled receptor 126 (GPR126), transcript variant a2, mRNA."
ILMN_1718731	KLK5	3.135594	1.4E-06	0.000439	8.794491	Up	"Homo sapiens kallikrein-related peptidase 5 (KLK5), transcript variant 2, mRNA."
ILMN_1702363	SULF1	3.49223	1.41E-06	0.00044	8.787201	Up	"Homo sapiens sulfatase 1 (SULF1), mRNA."
ILMN_3242004	ANXA8L1	3.758194	1.73E-06	0.000528	8.619605	Up	"Homo sapiens annexin A8-like 1 (ANXA8L1), mRNA."
ILMN_1816925	LOC728473	2.697548	1.73E-06	0.000528	8.618838	Up	"PREDICTED: Homo sapiens hypothetical LOC728473 (LOC728473), mRNA."
ILMN_3240912	LRRN4	2.804071	1.75E-06	0.000531	8.608597	Up	"Homo sapiens leucine rich repeat neuronal 4 (LRRN4), mRNA."
ILMN_1695745	DISP1	1.932126	1.77E-06	0.000534	8.598658	Up	"Homo sapiens dispatched homolog 1 (Drosophila) (DISP1), mRNA."
ILMN_1682226	CLDN15	2.713352	1.86E-06	0.000556	8.5604	Up	"Homo sapiens claudin 15 (CLDN15), mRNA."
ILMN_1722869	PDZD3	2.668778	1.95E-06	0.000577	8.519717	Up	"Homo sapiens PDZ domain containing 3 (PDZD3), mRNA."
ILMN_1717886	PKHD1L1	3.217471	1.98E-06	0.00058	8.507096	Up	"Homo sapiens polycystic kidney and hepatic disease 1 (autosomal recessive)-like 1 (PKHD1L1), mRNA."
ILMN_1746699	SGOL2	2.260661	1.99E-06	0.00058	8.50294	Up	"Homo sapiens shugoshin-like 2 (S. pombe) (SGOL2), mRNA."
ILMN_1696731	LOC652683	2.051993	2.02E-06	0.00058	8.492367	Up	"PREDICTED: Homo sapiens similar to sperm protein associated with the nucleus, X chromosome, family member B1 (LOC652683), mRNA."
ILMN_1691127	VTN	2.607338	2.02E-06	0.00058	8.490112	Up	"Homo sapiens vitronectin (VTN), mRNA."
ILMN_1796737	APM-1	1.702838	2.07E-06	0.00059	8.470807	Up	"PREDICTED: Homo sapiens BTB/POZ-zinc finger protein-like (APM-1), mRNA."
ILMN_1691616	LOC727935	1.804348	2.09E-06	0.00059	8.461898	Up	"PREDICTED: Homo sapiens similar to CHRNA7 (cholinergic receptor, nicotinic, alpha 7, exons 5-10) and FAM7A (family with sequence similarity 7A, exons A-E) fusion (LOC727935), mRNA."
ILMN_1778319	DMKN	2.72107	2.14E-06	0.000598	8.442916	Up	"Homo sapiens dermokine (DMKN), transcript variant 1, mRNA."
ILMN_3288032	LOC646576	2.678613	2.25E-06	0.00062	8.402909	Up	"PREDICTED: Homo sapiens hypothetical LOC646576 (LOC646576), miscRNA."
ILMN_1718046	ARNT2	2.196993	2.29E-06	0.000626	8.39058	Up	"Homo sapiens aryl-hydrocarbon receptor nuclear translocator 2 (ARNT2), mRNA."
ILMN_1716608	NGF	1.861727	2.41E-06	0.000648	8.347986	Up	"Homo sapiens nerve growth factor (beta polypeptide) (NGF), mRNA."

ILMN_1704294	CDH3	2.626307	2.46E-06	0.000658	8.331119	Up	"Homo sapiens cadherin 3, type 1, P-cadherin (placental) (CDH3), mRNA."
ILMN_1733110	RASSF7	2.586511	2.48E-06	0.000658	8.325354	Up	"Homo sapiens Ras association (RalGDS/AF-6) domain family (N-terminal) member 7 (RASSF7), mRNA."
ILMN_1666545	GCNT1	1.684177	2.49E-06	0.000658	8.322885	Up	"Homo sapiens glucosaminyl (N-acetyl) transferase 1, core 2 (beta-1,6-N-acetylglucosaminyltransferase) (GCNT1), transcript variant 4, mRNA."
ILMN_1718770	FLJ36070	2.370868	2.7E-06	0.000709	8.258821	Up	"Homo sapiens likely ortholog of MEF2-activating SAP transcriptional regulator (FLJ36070), mRNA."
ILMN_1753143	RHPN2	2.104419	2.86E-06	0.000743	8.212256	Up	"Homo sapiens rhophilin, Rho GTPase binding protein 2 (RHPN2), mRNA."
ILMN_2405667	CLDN15	2.813261	2.98E-06	0.00077	8.179407	Up	"Homo sapiens claudin 15 (CLDN15), transcript variant 2, mRNA."
ILMN_2339835	PTGS1	2.493566	3.08E-06	0.000788	8.152012	Up	"Homo sapiens prostaglandin-endoperoxide synthase 1 (prostaglandin G/H synthase and cyclooxygenase) (PTGS1), transcript variant 2, mRNA."
ILMN_1762531	FGF9	3.185156	3.17E-06	0.000802	8.129833	Up	"Homo sapiens fibroblast growth factor 9 (glia-activating factor) (FGF9), mRNA."
ILMN_2082865	PLLP	2.566417	3.3E-06	0.000825	8.099165	Up	"Homo sapiens plasma membrane proteolipid (plasmolipin) (PLLP), mRNA."
ILMN_1804351	FZD7	2.033119	3.43E-06	0.000844	8.068868	Up	"Homo sapiens frizzled homolog 7 (Drosophila) (FZD7), mRNA."
ILMN_1723962	LXN	1.754772	3.58E-06	0.000872	8.033215	Up	"Homo sapiens latexin (LXN), mRNA."
ILMN_1657760	SYT17	2.153077	3.59E-06	0.000872	8.031057	Up	"Homo sapiens synaptotagmin XVII (SYT17), mRNA."
ILMN_1758067	RGS4	2.761846	3.63E-06	0.000876	8.02381	Up	"Homo sapiens regulator of G-protein signalling 4 (RGS4), mRNA."
ILMN_1681983	RSPO3	1.750447	3.79E-06	0.000889	7.988951	Up	"Homo sapiens R-spondin 3 homolog (Xenopus laevis) (RSPO3), mRNA."
ILMN_1667162	NKX3-1	1.857274	3.86E-06	0.0009	7.975407	Up	"Homo sapiens NK3 homeobox 1 (NKX3-1), mRNA."
ILMN_1695945	MEIS2	1.739552	3.89E-06	0.000902	7.969435	Up	"Homo sapiens Meis homeobox 2 (MEIS2), transcript variant g, mRNA."
ILMN_3246728	C13ORF36	3.765265	3.96E-06	0.000914	7.955722	Up	"Homo sapiens chromosome 13 open reading frame 36 (C13orf36), mRNA."
ILMN_1660727	ENPP5	1.77666	3.99E-06	0.000917	7.949111	Up	"Homo sapiens ectonucleotide pyrophosphatase/phosphodiesterase 5 (putative function) (ENPP5), mRNA."
ILMN_1752884	AADACL2	2.595614	4.25E-06	0.000967	7.900815	Up	"Homo sapiens arylacetamide deacetylase-like 2 (AADACL2), mRNA."
ILMN_1696657	LRRN2	1.906481	4.29E-06	0.00097	7.893695	Up	"Homo sapiens leucine rich repeat neuronal 2 (LRRN2), transcript variant 2, mRNA."
ILMN_1770338	TM4SF1	1.364928	4.32E-06	0.00097	7.888162	Up	"Homo sapiens transmembrane 4 L six family member 1 (TM4SF1), mRNA."
ILMN_1763491	CKMT1B	1.740596	4.36E-06	0.00097	7.880575	Up	"Homo sapiens creatine kinase, mitochondrial 1B (CKMT1B), nuclear gene encoding mitochondrial protein, mRNA."
ILMN_2341548	MYO5B	2.097867	4.36E-06	0.00097	7.88006	Up	"Homo sapiens myosin VB (MYO5B), mRNA."
ILMN_1659631	LOC730024	1.621957	4.37E-06	0.00097	7.879882	Up	"PREDICTED: Homo sapiens similar to male sterility domain containing 1 (LOC730024), mRNA."
ILMN_2391264	DMKN	2.628842	4.93E-06	0.001064	7.787093	Up	"Homo sapiens dermokine (DMKN), transcript variant 2, mRNA."
ILMN_2311020	DNAJC12	1.411457	5.05E-06	0.00108	7.768602	Up	"Homo sapiens DnaJ (Hsp40) homolog, subfamily C, member 12 (DNAJC12), transcript variant 2, mRNA."
ILMN_1713182	LOC653879	1.889046	5.06E-06	0.00108	7.765824	Up	"PREDICTED: Homo sapiens similar to Complement C3 precursor (LOC653879), mRNA."
ILMN_2413780	SEZ6L2	1.41428	5.22E-06	0.001108	7.742654	Up	"Homo sapiens seizure related 6 homolog (mouse)-like 2 (SEZ6L2), transcript variant 2, mRNA."
ILMN_2405254	GRB7	1.544549	5.62E-06	0.001172	7.686265	Up	"Homo sapiens growth factor receptor-bound protein 7 (GRB7), transcript variant 2, mRNA."
ILMN_1716397	LAYN	2.095069	5.77E-06	0.001186	7.666123	Up	"Homo sapiens layilin (LAYN), mRNA."
ILMN_1779182	TMEM98	2.051822	5.79E-06	0.001186	7.663972	Up	"Homo sapiens transmembrane protein 98 (TMEM98), transcript variant 2, mRNA."
ILMN_1721134	TGM1	3.333386	6.09E-06	0.001231	7.625772	Up	"Homo sapiens transglutaminase 1 (K polypeptide epidermal type I, protein-glutamine-gamma-glutamyltransferase) (TGM1), mRNA."
ILMN_2317751	REC8	2.25329	6.14E-06	0.001231	7.619525	Up	"Homo sapiens REC8 homolog (yeast) (REC8), transcript variant 1, mRNA."

ILMN_1662166	PTK7	1.829408	6.2E-06	0.001236	7.612695	Up	"Homo sapiens PTK7 protein tyrosine kinase 7 (PTK7), transcript variant PTK7-4, mRNA."
ILMN_2065022	KIAA0672	2.640527	6.22E-06	0.001236	7.609776	Up	"Homo sapiens KIAA0672 gene product (KIAA0672), mRNA."
ILMN_1695404	LY6E	1.368312	6.28E-06	0.001239	7.602136	Up	"Homo sapiens lymphocyte antigen 6 complex, locus E (LY6E), mRNA."
ILMN_1816603	HS.31961	1.283292	6.36E-06	0.001249	7.592761	Up	"Homo sapiens cDNA FLJ37694 fis, clone BRHIP2015224"
ILMN_1797526	LOC400120	3.537561	6.53E-06	0.001278	7.572648	Up	"Homo sapiens hypothetical LOC400120 (LOC400120), mRNA."
ILMN_1759766	CTXN1	3.47018	6.82E-06	0.001314	7.54055	Up	"Homo sapiens cortexin 1 (CTXN1), mRNA."
ILMN_2068104	TFPI2	2.774673	6.86E-06	0.001314	7.53643	Up	"Homo sapiens tissue factor pathway inhibitor 2 (TFPI2), mRNA."
ILMN_1658071	ATP1B1	1.648157	6.92E-06	0.001319	7.529286	Up	"Homo sapiens ATPase, Na+/K+ transporting, beta 1 polypeptide (ATP1B1), transcript variant 1, mRNA."
ILMN_2407703	SYN1	1.281211	6.94E-06	0.001319	7.527473	Up	"Homo sapiens synapsin I (SYN1), transcript variant Ia, mRNA."
ILMN_1682326	PCP4	3.545234	7.2E-06	0.001347	7.499895	Up	"Homo sapiens Purkinje cell protein 4 (PCP4), mRNA."
ILMN_3245564	RICH2	2.530368	7.39E-06	0.001377	7.480547	Up	"Homo sapiens Rho-type GTPase-activating protein RICH2 (RICH2), mRNA."
ILMN_1760153	GATA5	2.98878	7.9E-06	0.001467	7.430379	Up	"Homo sapiens GATA binding protein 5 (GATA5), mRNA."
ILMN_1804673	SLC16A4	1.821959	8.05E-06	0.001479	7.417072	Up	"Homo sapiens solute carrier family 16, member 4 (monocarboxylic acid transporter 5) (SLC16A4), mRNA."
ILMN_1724480	AXIN2	1.456288	8.1E-06	0.001479	7.412293	Up	"Homo sapiens axin 2 (conductin, axil) (AXIN2), mRNA."
ILMN_1734366	RORC	2.541547	9.04E-06	0.001614	7.331292	Up	"Homo sapiens RAR-related orphan receptor C (RORC), transcript variant 2, mRNA."
ILMN_1806787	CSDC2	2.055096	9.29E-06	0.001641	7.311439	Up	"Homo sapiens cold shock domain containing C2, RNA binding (CSDC2), mRNA."
ILMN_1751886	REC8	2.529298	9.32E-06	0.001641	7.309212	Up	"Homo sapiens REC8 homolog (yeast) (REC8), transcript variant 1, mRNA."
ILMN_1731561	ROBO3	1.635066	9.35E-06	0.001641	7.306176	Up	"Homo sapiens roundabout, axon guidance receptor, homolog 3 (Drosophila) (ROBO3), mRNA."
ILMN_2217601	ANXA9	2.276037	9.39E-06	0.001641	7.303006	Up	"Homo sapiens annexin A9 (ANXA9), mRNA."
ILMN_1774287	CFB	2.940322	9.48E-06	0.001648	7.296754	Up	"Homo sapiens complement factor B (CFB), mRNA."
ILMN_2353202	PTK7	2.268206	9.68E-06	0.001679	7.280723	Up	"Homo sapiens PTK7 protein tyrosine kinase 7 (PTK7), transcript variant PTK7-2, mRNA."
ILMN_1751346	ERBB3	1.96611	1.09E-05	0.001855	7.192967	Up	"Homo sapiens v-erb-b2 erythroblastic leukemia viral oncogene homolog 3 (avian) (ERBB3), transcript variant 1, mRNA."
ILMN_1730229	CGNL1	2.190276	1.09E-05	0.001855	7.192222	Up	"Homo sapiens cingulin-like 1 (CGNL1), mRNA."
ILMN_1662390	ASPHD1	1.708512	1.11E-05	0.001865	7.182906	Up	"Homo sapiens aspartate beta-hydroxylase domain containing 1 (ASPHD1), mRNA."
ILMN_1717262	PROCR	2.385376	1.13E-05	0.001892	7.167481	Up	"Homo sapiens protein C receptor, endothelial (EPCR) (PROCR), mRNA."
ILMN_1809639	TMEM26	1.324194	1.14E-05	0.001894	7.164273	Up	"Homo sapiens transmembrane protein 26 (TMEM26), mRNA."
ILMN_1733746	REEP1	3.153963	1.14E-05	0.001894	7.161482	Up	"Homo sapiens receptor accessory protein 1 (REEP1), mRNA."
ILMN_2328776	MST4	2.798005	1.16E-05	0.001903	7.151526	Up	"Homo sapiens serine/threonine protein kinase MST4 (MST4), transcript variant 2, mRNA."
ILMN_1752965	GREM1	2.359404	1.16E-05	0.001903	7.14801	Up	"Homo sapiens gremlin 1, cysteine knot superfamily, homolog (Xenopus laevis) (GREM1), mRNA."
ILMN_1804662	NRG4	1.571959	1.18E-05	0.00193	7.135205	Up	"Homo sapiens neuregulin 4 (NRG4), mRNA."
ILMN_1691790	DACT2	2.328308	1.2E-05	0.001953	7.124431	Up	"Homo sapiens dapper, antagonist of beta-catenin, homolog 2 (Xenopus laevis) (DACT2), mRNA."
ILMN_1688154	MST1R	1.563182	1.26E-05	0.002034	7.092512	Up	"Homo sapiens macrophage stimulating 1 receptor (c-met-related tyrosine kinase) (MST1R), mRNA."
ILMN_1749474	FAM7A1	1.55779	1.28E-05	0.002055	7.08038	Up	"PREDICTED: Homo sapiens family with sequence similarity 7, member A1, transcript variant 4 (FAM7A1), mRNA."
ILMN_1740728	SMTNL2	2.144266	1.3E-05	0.002074	7.068826	Up	"Homo sapiens smoothelin-like 2 (SMTNL2), mRNA."
ILMN_1695041	GATA6	1.258501	1.3E-05	0.002074	7.068658	Up	"Homo sapiens GATA binding protein 6 (GATA6), mRNA."

ILMN_1676215	DLG2	1.96164	1.31E-05	0.002085	7.062482	Up	"Homo sapiens discs, large homolog 2, chapsyn-110 (Drosophila) (DLG2), mRNA."
ILMN_2111229	BZRAP1	1.354025	1.34E-05	0.002123	7.044921	Up	"Homo sapiens benzodiazapine receptor (peripheral) associated protein 1 (BZRAP1), mRNA."
ILMN_1708580	PDZK1IP1	1.753909	1.36E-05	0.002126	7.036788	Up	"Homo sapiens PDZK1 interacting protein 1 (PDZK1IP1), mRNA."
ILMN_1722056	ATP7B	1.676645	1.4E-05	0.002168	7.012248	Up	"Homo sapiens ATPase, Cu++ transporting, beta polypeptide (ATP7B), transcript variant 1, mRNA."
ILMN_1873278	LOC731895	2.348424	1.47E-05	0.002245	6.979063	Up	"PREDICTED: Homo sapiens similar to transmembrane protein 28 (LOC731895), mRNA."
ILMN_1779855	HSD17B6	3.946054	1.56E-05	0.002342	6.935512	Up	"Homo sapiens hydroxysteroid (17-beta) dehydrogenase 6 homolog (mouse) (HSD17B6), mRNA."
ILMN_1798690	ADAMTSL3	1.70997	1.62E-05	0.002421	6.908997	Up	"Homo sapiens ADAMTS-like 3 (ADAMTSL3), mRNA."
ILMN_2043809	PFKM	1.373457	1.7E-05	0.002521	6.874418	Up	"Homo sapiens phosphofructokinase, muscle (PFKM), mRNA."
ILMN_1833858	HS.66187	1.386655	1.79E-05	0.002623	6.839835	Up	Homo sapiens clone 23700 mRNA sequence
ILMN_1736412	AMHR2	2.260726	1.82E-05	0.002648	6.826794	Up	"Homo sapiens anti-Mullerian hormone receptor, type II (AMHR2), mRNA."
ILMN_1695316	SLC39A8	2.552958	1.87E-05	0.002701	6.810624	Up	"Homo sapiens solute carrier family 39 (zinc transporter), member 8 (SLC39A8), transcript variant 1, mRNA."
ILMN_1776724	LYPD6	2.098231	1.88E-05	0.002712	6.804506	Up	"Homo sapiens LY6/PLAUR domain containing 6 (LYPD6), mRNA."
ILMN_1713995	SCNN1A	1.758591	1.89E-05	0.002712	6.803637	Up	"Homo sapiens sodium channel, nonvoltage-gated 1 alpha (SCNN1A), mRNA."
ILMN_1745817	NELL1	2.505614	1.92E-05	0.002752	6.789061	Up	"Homo sapiens NEL-like 1 (chicken) (NELL1), mRNA."
ILMN_1699978	FAM70A	1.963188	2.06E-05	0.002895	6.743155	Up	"Homo sapiens family with sequence similarity 70, member A (FAM70A), mRNA."
ILMN_1699768	CBLN4	2.1116	2.08E-05	0.002913	6.734822	Up	"Homo sapiens cerebellin 4 precursor (CBLN4), mRNA."
ILMN_1693836	LOC653344	2.504504	2.12E-05	0.002937	6.722834	Up	"PREDICTED: Homo sapiens similar to cis-Golgi matrix protein GM130, transcript variant 2 (LOC653344), mRNA."
ILMN_1787673	PLLP	2.139423	2.15E-05	0.002963	6.71074	Up	"Homo sapiens plasma membrane proteolipid (plasmolipin) (PLLP), mRNA."
ILMN_1687867	LOC647954	1.998641	2.2E-05	0.002988	6.696089	Up	"PREDICTED: Homo sapiens misc_RNA (LOC647954), miscRNA."
ILMN_1715068	AQP9	2.671201	2.23E-05	0.002988	6.686865	Up	"Homo sapiens aquaporin 9 (AQP9), mRNA."
ILMN_1738684	NRXN2	1.66062	2.38E-05	0.003167	6.642662	Up	"Homo sapiens neurexin 2 (NRXN2), transcript variant beta, mRNA."
ILMN_1727087	GJA1	1.493614	2.44E-05	0.00322	6.623608	Up	"Homo sapiens gap junction protein, alpha 1, 43kDa (GJA1), mRNA."
ILMN_1656373	BNC2	1.457398	2.46E-05	0.003227	6.618235	Up	"Homo sapiens basonuclin 2 (BNC2), mRNA."
ILMN_1772312	BARX1	2.076594	2.53E-05	0.003291	6.598051	Up	"Homo sapiens BARX homeobox 1 (BARX1), mRNA."
ILMN_1746784	SLAIN1	1.585206	2.55E-05	0.003301	6.593055	Up	"Homo sapiens SLAIN motif family, member 1 (SLAIN1), transcript variant 1, mRNA."
ILMN_1711566	TIMP1	1.506015	2.62E-05	0.003367	6.575826	Up	"Homo sapiens TIMP metallopeptidase inhibitor 1 (TIMP1), mRNA."
ILMN_1813100	KIAA1244	1.380956	2.72E-05	0.003471	6.549343	Up	"Homo sapiens KIAA1244 (KIAA1244), mRNA."
ILMN_1778333	MMP24	1.87809	2.76E-05	0.00349	6.540064	Up	"Homo sapiens matrix metallopeptidase 24 (membrane-inserted) (MMP24), mRNA."
ILMN_1652456	TMEM98	2.183557	2.79E-05	0.003526	6.531227	Up	"Homo sapiens transmembrane protein 98 (TMEM98), transcript variant 1, mRNA."
ILMN_1677505	CCL21	3.631937	2.83E-05	0.003561	6.522231	Up	"Homo sapiens chemokine (C-C motif) ligand 21 (CCL21), mRNA."
ILMN_1695606	EFNB3	2.595261	2.85E-05	0.003571	6.517153	Up	"Homo sapiens ephrin-B3 (EFNB3), mRNA."
ILMN_1811387	TFF3	3.407857	2.92E-05	0.003633	6.501418	Up	"Homo sapiens trefoil factor 3 (intestinal) (TFF3), mRNA."
ILMN_1812679	UPK1B	2.592163	2.99E-05	0.003679	6.486055	Up	"Homo sapiens uroplakin 1B (UPK1B), mRNA."
ILMN_1680925	SLC9A3R1	1.578439	3.02E-05	0.00371	6.478653	Up	"Homo sapiens solute carrier family 9 (sodium/hydrogen exchanger), member 3 regulator 1 (SLC9A3R1), mRNA."
ILMN_2386982	PRKCZ	1.803615	3.05E-05	0.003722	6.471953	Up	"Homo sapiens protein kinase C, zeta (PRKCZ), transcript variant 1, mRNA."

ILMN_1754103	CLDN11	1.833073	3.09E-05	0.003746	6.463242	Up	"Homo sapiens claudin 11 (oligodendrocyte transmembrane protein) (CLDN11), mRNA."
ILMN_1811133	LOC645464	2.502862	3.14E-05	0.003788	6.452321	Up	"PREDICTED: Homo sapiens similar to vasoactive intestinal peptide receptor 2 (LOC645464), mRNA."
ILMN_1803855	FAIM2	1.801051	3.21E-05	0.00383	6.437822	Up	"Homo sapiens Fas apoptotic inhibitory molecule 2 (FAIM2), mRNA."
ILMN_3254481	LOC100128893	2.088876	3.29E-05	0.003909	6.42068	Up	"PREDICTED: Homo sapiens hypothetical protein LOC100128893 (LOC100128893), mRNA."
ILMN_1769388	GJB2	2.07804	3.3E-05	0.003915	6.417999	Up	"Homo sapiens gap junction protein, beta 2, 26kDa (GJB2), mRNA."
ILMN_1741406	HOOK1	1.508036	3.33E-05	0.003926	6.412872	Up	"Homo sapiens hook homolog 1 (Drosophila) (HOOK1), mRNA."
ILMN_1742534	COL4A5	1.912801	3.35E-05	0.003936	6.407654	Up	"Homo sapiens collagen, type IV, alpha 5 (COL4A5), transcript variant 1, mRNA."
ILMN_1738742	PLAT	2.099942	3.46E-05	0.004027	6.387078	Up	"Homo sapiens plasminogen activator, tissue (PLAT), transcript variant 1, mRNA."
ILMN_3283316	LOC255167	2.454383	3.49E-05	0.004038	6.380258	Up	"Homo sapiens hypothetical LOC255167 (LOC255167), non-coding RNA."
ILMN_2064902	HAND2	1.719838	3.61E-05	0.004128	6.357356	Up	"Homo sapiens heart and neural crest derivatives expressed 2 (HAND2), mRNA."
ILMN_1658356	PAMR1	2.41916	3.79E-05	0.004273	6.324923	Up	"Homo sapiens peptidase domain containing associated with muscle regeneration 1 (PAMR1), transcript variant 1, mRNA."
ILMN_1656837	RBP1	2.188879	3.79E-05	0.004273	6.324369	Up	"Homo sapiens retinol binding protein 1, cellular (RBP1), mRNA."
ILMN_1779228	CDH2	1.762561	3.95E-05	0.004396	6.297512	Up	"Homo sapiens cadherin 2, type 1, N-cadherin (neuronal) (CDH2), mRNA."
ILMN_1655614	DSP	2.292337	4.03E-05	0.00445	6.28303	Up	"Homo sapiens desmoplakin (DSP), transcript variant 2, mRNA."
ILMN_1676413	VSNL1	1.960397	4.13E-05	0.004507	6.267106	Up	"Homo sapiens visinin-like 1 (VSNL1), mRNA."
ILMN_2086612	CMAH	1.791827	4.17E-05	0.00452	6.2603	Up	Homo sapiens cytidine monophosphate-N-acetylneuraminic acid hydroxylase (CMP-N-acetylneuraminate monooxygenase) (CMAH) on chromosome 6.
ILMN_1814629	ZC3H12B	1.298513	4.26E-05	0.004567	6.247272	Up	"Homo sapiens zinc finger CCCH-type containing 12B (ZC3H12B), mRNA."
ILMN_1716869	GPM6A	1.932099	4.32E-05	0.00463	6.236655	Up	"Homo sapiens glycoprotein M6A (GPM6A), transcript variant 3, mRNA."
ILMN_1653415	SPANXA2	1.599955	4.5E-05	0.004796	6.210309	Up	"Homo sapiens SPANX family, member A2 (SPANXA2), mRNA."
ILMN_2080684	IQCA1	1.453943	4.56E-05	0.004835	6.20192	Up	"Homo sapiens IQ motif containing with AAA domain 1 (IQCA1), mRNA."
ILMN_2407824	ATP1B1	1.457675	4.69E-05	0.004942	6.183235	Up	"Homo sapiens ATPase, Na+/K+ transporting, beta 1 polypeptide (ATP1B1), transcript variant 2, mRNA."
ILMN_1811779	MGC24103	1.517181	4.72E-05	0.004963	6.178743	Up	"PREDICTED: Homo sapiens hypothetical protein MGC24103 (MGC24103), misc RNA."
ILMN_1684959	ASTN1	1.853175	4.74E-05	0.004977	6.17539	Up	"Homo sapiens astrotactin 1 (ASTN1), transcript variant 1, mRNA."
ILMN_3248511	FAM167A	1.938317	4.87E-05	0.005081	6.157258	Up	"Homo sapiens family with sequence similarity 167, member A (FAM167A), mRNA."
ILMN_2371911	MUC1	1.848117	4.89E-05	0.005084	6.155488	Up	"Homo sapiens mucin 1, cell surface associated (MUC1), transcript variant 5, mRNA."
ILMN_1695946	TRNP1	1.669292	4.91E-05	0.005088	6.152085	Up	"Homo sapiens TMF1-regulated nuclear protein 1 (TRNP1), mRNA."
ILMN_1755383	LRRC1	2.11411	5.15E-05	0.005279	6.121001	Up	"Homo sapiens leucine rich repeat containing 1 (LRRC1), mRNA."
ILMN_1803024	UPK3B	3.90218	5.26E-05	0.005329	6.106859	Up	"Homo sapiens uroplakin 3B (UPK3B), transcript variant 1, mRNA."
ILMN_1791679	DNER	2.495098	5.28E-05	0.005329	6.104762	Up	"Homo sapiens delta/notch-like EGF repeat containing (DNER), mRNA."
ILMN_1666536	VSIG2	1.551794	5.37E-05	0.005358	6.09394	Up	"Homo sapiens V-set and immunoglobulin domain containing 2 (VSIG2), mRNA."
ILMN_1762260	C3	1.950347	5.48E-05	0.005448	6.080246	Up	"Homo sapiens complement component 3 (C3), mRNA."
ILMN_1802316	SMPD3	2.113503	5.59E-05	0.00552	6.067458	Up	"Homo sapiens sphingomyelin phosphodiesterase 3, neutral membrane (neutral sphingomyelinase II) (SMPD3), mRNA."
ILMN_1779374	AMMECR1	1.565546	5.99E-05	0.005848	6.021463	Up	"Homo sapiens Alport syndrome, mental retardation, midface hypoplasia and elliptocytosis chromosomal

							region gene 1 (AMMECR1), transcript variant 1, mRNA."
ILMN_1838863	HS.497591	1.858529	6.07E-05	0.005874	6.013432	Up	"Homo sapiens cDNA FLJ41846 fis, clone NT2RI3003162"
ILMN_1708340	DAPK1	1.807793	6.09E-05	0.005886	6.010754	Up	"Homo sapiens death-associated protein kinase 1 (DAPK1), mRNA."
ILMN_1886092	HS.383564	1.355482	6.26E-05	0.006006	5.992933	Up	"Homo sapiens mRNA for KIAA0574 protein, partial cds"
ILMN_2413158	PODXL	1.43928	6.27E-05	0.006006	5.991869	Up	"Homo sapiens podocalyxin-like (PODXL), transcript variant 1, mRNA."
ILMN_1772910	GAS1	1.530076	6.5E-05	0.006187	5.969057	Up	"Homo sapiens growth arrest-specific 1 (GAS1), mRNA."
ILMN_1789040	SLITRK5	1.689541	6.57E-05	0.006239	5.962341	Up	"Homo sapiens SLIT and NTRK-like family, member 5 (SLITRK5), mRNA."
ILMN_1708267	CLDN15	1.727801	6.64E-05	0.006288	5.954634	Up	"Homo sapiens claudin 15 (CLDN15), transcript variant 2, mRNA."
ILMN_3184067	FLJ30428	1.565748	6.7E-05	0.006304	5.94907	Up	"PREDICTED: Homo sapiens similar to hypothetical protein A230046P18; cDNA sequence BC055759, transcript variant 1 (FLJ30428), mRNA."
ILMN_1697267	PRKCZ	1.309813	6.87E-05	0.006386	5.933027	Up	"Homo sapiens protein kinase C, zeta (PRKCZ), transcript variant 1, mRNA."
ILMN_1692748	BNC1	2.182669	6.91E-05	0.006415	5.928875	Up	"Homo sapiens basonuclin 1 (BNC1), mRNA."
ILMN_1779234	CXCL6	2.88685	7.12E-05	0.006514	5.910114	Up	"Homo sapiens chemokine (C-X-C motif) ligand 6 (granulocyte chemotactic protein 2) (CXCL6), mRNA."
ILMN_3241944	NBLA00301	1.591827	7.43E-05	0.006777	5.882059	Up	"Homo sapiens Nbla00301 (NBLA00301), non-coding RNA."
ILMN_2353276	UPK3B	3.848915	7.47E-05	0.006799	5.878786	Up	"Homo sapiens uroplakin 3B (UPK3B), transcript variant 2, mRNA."
ILMN_1880012	SEMA5A	1.278328	7.65E-05	0.006926	5.863406	Up	"Homo sapiens sema domain, seven thrombospondin repeats (type 1 and type 1-like), transmembrane domain (TM) and short cytoplasmic domain, (semaphorin) 5A (SEMA5A), mRNA."
ILMN_1778964	CLIC5	2.321624	7.65E-05	0.006926	5.863158	Up	"Homo sapiens chloride intracellular channel 5 (CLIC5), mRNA."
ILMN_1748970	PRR15L	1.850894	7.87E-05	0.007057	5.845183	Up	"Homo sapiens proline rich 15-like (PRR15L), mRNA."
ILMN_1723443	LRP2	2.62107	7.94E-05	0.007105	5.83937	Up	"Homo sapiens low density lipoprotein-related protein 2 (LRP2), mRNA."
ILMN_1783149	CDH23	1.50399	8.37E-05	0.007339	5.805459	Up	"Homo sapiens cadherin-like 23 (CDH23), transcript variant 1, mRNA."
ILMN_1687306	LGALS2	1.725481	8.59E-05	0.007482	5.789528	Up	"Homo sapiens lectin, galactoside-binding, soluble, 2 (LGALS2), mRNA."
ILMN_2403534	ALOX15	1.360589	8.64E-05	0.007499	5.785772	Up	"Homo sapiens arachidonate 15-lipoxygenase (ALOX15), mRNA."
ILMN_2264177	UPK3B	4.333265	8.77E-05	0.007567	5.775635	Up	"Homo sapiens uroplakin 3B (UPK3B), transcript variant 2, mRNA."
ILMN_1769615	FLRT2	1.841878	9.18E-05	0.007815	5.746568	Up	"Homo sapiens fibronectin leucine rich transmembrane protein 2 (FLRT2), mRNA."
ILMN_1797557	PLEKHA6	1.986718	9.23E-05	0.007815	5.743237	Up	"Homo sapiens pleckstrin homology domain containing, family A member 6 (PLEKHA6), mRNA."
ILMN_1802646	EPHB6	1.421191	9.38E-05	0.007923	5.733369	Up	"Homo sapiens EPH receptor B6 (EPHB6), mRNA."
ILMN_1705258	CPZ	1.77059	0.000101	0.00836	5.689182	Up	"Homo sapiens carboxypeptidase Z (CPZ), transcript variant 1, mRNA."
ILMN_1749466	VAT1L	2.121328	0.000101	0.00841	5.68322	Up	"Homo sapiens vesicle amine transport protein 1 homolog (T. californica)-like (VAT1L), mRNA."
ILMN_1694767	LOC652688	2.474429	0.000103	0.008494	5.67273	Up	"PREDICTED: Homo sapiens similar to Zinc finger protein basonuclin-1 (LOC652688), mRNA."
ILMN_2108735	EEF1A2	2.042384	0.000103	0.008494	5.671747	Up	"Homo sapiens eukaryotic translation elongation factor 1 alpha 2 (EEF1A2), mRNA."
ILMN_1785900	LOC653108	1.636326	0.000104	0.008494	5.670695	Up	"PREDICTED: Homo sapiens similar to coxsackie virus and adenovirus receptor precursor (LOC653108), mRNA."
ILMN_2337923	TPD52L1	1.275569	0.000108	0.008712	5.645633	Up	"Homo sapiens tumor protein D52-like 1 (TPD52L1), transcript variant 4, mRNA."
ILMN_1701933	SNCA	1.669612	0.00011	0.00886	5.631796	Up	"Homo sapiens synuclein, alpha (non A4 component of amyloid precursor) (SNCA), transcript variant NACP112, mRNA."
ILMN_1801090	KRT222	1.473982	0.000111	0.008898	5.628033	Up	"Homo sapiens keratin 222 (KRT222), mRNA."

ILMN_2139970	ALDH1A3	1.378743	0.000111	0.008914	5.624748	Up	"Homo sapiens aldehyde dehydrogenase 1 family, member A3 (ALDH1A3), mRNA."
ILMN_1812070	ABCB1	1.995204	0.000114	0.009045	5.609184	Up	"Homo sapiens ATP-binding cassette, sub-family B (MDR/TAP), member 1 (ABCB1), mRNA."
ILMN_2150802	FLJ22795	1.546255	0.000119	0.009319	5.585122	Up	"Homo sapiens hypothetical protein FLJ22795 (FLJ22795), mRNA."
ILMN_1658619	WWC*1*	1.600551	0.000124	0.009587	5.558157	Up	"Homo sapiens WW and C2 domain containing 1 (WWC1), mRNA."
ILMN_2407879	SORBS2	1.687453	0.000131	0.009981	5.520787	Up	"Homo sapiens sorbin and SH3 domain containing 2 (SORBS2), transcript variant 1, mRNA."
ILMN_1805561	SLC14A1	1.961273	0.000141	0.01046	5.477157	Up	"Homo sapiens solute carrier family 14 (urea transporter), member 1 (Kidd blood group) (SLC14A1), mRNA."
ILMN_3272441	LOC100129165	1.703302	0.000141	0.01046	5.476915	Up	"PREDICTED: Homo sapiens similar to Phosphotidylinositol phosphatase PTPRQ precursor (Receptor-type tyrosine-protein phosphatase Q) (PTP-RQ) (LOC100129165), mRNA."
ILMN_1734897	SLC4A4	1.644504	0.000143	0.010547	5.466809	Up	"Homo sapiens solute carrier family 4, sodium bicarbonate cotransporter, member 4 (SLC4A4), transcript variant 2, mRNA."
ILMN_3179038	LOC100127909	1.725405	0.000145	0.010561	5.461037	Up	"PREDICTED: Homo sapiens hypothetical protein LOC100127909 (LOC100127909), mRNA."
ILMN_3244876	LOC100133171	1.683527	0.000146	0.010583	5.456039	Up	"PREDICTED: Homo sapiens hypothetical protein LOC100133171 (LOC100133171), mRNA."
ILMN_1736911	TMOD1	1.304559	0.000151	0.010859	5.436326	Up	"Homo sapiens tropomodulin 1 (TMOD1), mRNA."
ILMN_1651900	KCNA4	1.354147	0.000152	0.010901	5.432192	Up	"Homo sapiens potassium voltage-gated channel, shaker-related subfamily, member 4 (KCNA4), mRNA."
ILMN_1748827	LOC388564	1.412375	0.000152	0.010901	5.432	Up	"PREDICTED: Homo sapiens hypothetical gene supported by BC052596 (LOC388564), mRNA."
ILMN_1679464	BNC1	2.133631	0.000152	0.010926	5.429688	Up	"Homo sapiens basonuclin 1 (BNC1), mRNA."
ILMN_1707925	ABHD12B	1.473699	0.000153	0.010937	5.426921	Up	"Homo sapiens abhydrolase domain containing 12B (ABHD12B), transcript variant 2, mRNA."
ILMN_1779547	HPSE	1.312435	0.000156	0.01102	5.415965	Up	"Homo sapiens heparanase (HPSE), mRNA."
ILMN_1790098	OGN	2.562591	0.00016	0.011221	5.400226	Up	"Homo sapiens osteoglycin (OGN), transcript variant 3, mRNA."
ILMN_1799028	TSPAN5	1.830892	0.000161	0.011302	5.393016	Up	"Homo sapiens tetraspanin 5 (TSPAN5), mRNA."
ILMN_2205470	FAM153B	1.748845	0.000162	0.011302	5.391171	Up	"Homo sapiens family with sequence similarity 153, member B (FAM153B), mRNA."
ILMN_1804604	C1QTNF3	1.27695	0.000162	0.011304	5.389574	Up	"Homo sapiens C1q and tumor necrosis factor related protein 3 (C1QTNF3), transcript variant 2, mRNA."
ILMN_1866954	HS.71947	1.358086	0.00017	0.01167	5.360326	Up	Homo sapiens mRNA full length insert cDNA clone EUROIMAGE 994183
ILMN_1736692	SRL	1.24493	0.00017	0.01167	5.359791	Up	"Homo sapiens sarcalumenin (SRL), mRNA."
ILMN_1654598	IQCA1	1.36477	0.000174	0.011813	5.346916	Up	"Homo sapiens IQ motif containing with AAA domain 1 (IQCA1), mRNA."
ILMN_2315789	PTPRD	1.507394	0.000176	0.011856	5.339446	Up	"Homo sapiens protein tyrosine phosphatase, receptor type, D (PTPRD), transcript variant 2, mRNA."
ILMN_1716407	SORBS2	1.703559	0.000176	0.011856	5.338553	Up	"Homo sapiens sorbin and SH3 domain containing 2 (SORBS2), transcript variant 1, mRNA."
ILMN_1807439	ALDH1A3	1.445656	0.000181	0.012099	5.321776	Up	"Homo sapiens aldehyde dehydrogenase 1 family, member A3 (ALDH1A3), mRNA."
ILMN_1655949	LOC653107	1.785914	0.000183	0.012176	5.316155	Up	"PREDICTED: Homo sapiens similar to Annexin A8 (Annexin VIII) (Vascular anticoagulant-beta) (VAC-beta), transcript variant 5 (LOC653107), mRNA."
ILMN_1912619	HS.568058	1.661667	0.000185	0.012294	5.30799	Up	full-length cDNA clone CS0DF032YD19 of Fetal brain of Homo sapiens (human)
ILMN_3240901	ZBTB7C	1.34328	0.000192	0.012616	5.287558	Up	"Homo sapiens zinc finger and BTB domain containing 7C (ZBTB7C), mRNA."
ILMN_1723969	PLCB1	1.305088	0.000194	0.012683	5.280362	Up	"Homo sapiens phospholipase C, beta 1 (phosphoinositide-specific) (PLCB1), transcript variant 1, mRNA."
ILMN_1776151	OLFML1	1.349829	0.000196	0.012774	5.273223	Up	"Homo sapiens olfactomedin-like 1 (OLFML1), mRNA."
ILMN_1667169	LOC440349	1.69436	0.000197	0.012796	5.270496	Up	"PREDICTED: Homo sapiens similar to nuclear pore complex interacting protein, transcript variant 2 (LOC440349), mRNA."

ILMN_2406299	SEMA3B	1.498463	0.000198	0.012829	5.266384	Up	"Homo sapiens sema domain, immunoglobulin domain (Ig), short basic domain, secreted, (semaphorin) 3B (SEMA3B), transcript variant 2, mRNA."
ILMN_1678095	SMPDL3B	1.308765	0.000206	0.013139	5.24353	Up	"Homo sapiens sphingomyelin phosphodiesterase, acid-like 3B (SMPDL3B), transcript variant 1, mRNA."
ILMN_1661491	SH3GL2	1.664493	0.000207	0.013158	5.240231	Up	"Homo sapiens SH3-domain GRB2-like 2 (SH3GL2), mRNA."
ILMN_1653501	SEMA3B	1.403151	0.00021	0.013275	5.232393	Up	"Homo sapiens sema domain, immunoglobulin domain (Ig), short basic domain, secreted, (semaphorin) 3B (SEMA3B), transcript variant 2, mRNA."
ILMN_1701700	KLHL4	1.530223	0.000212	0.013344	5.226844	Up	"Homo sapiens kelch-like 4 (Drosophila) (KLHL4), transcript variant 1, mRNA."
ILMN_1725193	IGFBP2	1.543893	0.000217	0.013552	5.213349	Up	"Homo sapiens insulin-like growth factor binding protein 2, 36kDa (IGFBP2), mRNA."
ILMN_1674367	SPRR2F	1.813995	0.000223	0.013862	5.194829	Up	"Homo sapiens small proline-rich protein 2F (SPRR2F), mRNA."
ILMN_1714383	TPD52L1	1.261852	0.000226	0.013939	5.188272	Up	"Homo sapiens tumor protein D52-like 1 (TPD52L1), transcript variant 3, mRNA."
ILMN_1692535	DPP4	1.38184	0.000233	0.014254	5.167699	Up	"Homo sapiens dipeptidyl-peptidase 4 (DPP4), mRNA."
ILMN_1755721	FAM63A	1.558775	0.000237	0.014392	5.158205	Up	"Homo sapiens family with sequence similarity 63, member A (FAM63A), transcript variant 1, mRNA."
ILMN_1725139	CA9	1.732906	0.000244	0.014728	5.140906	Up	"Homo sapiens carbonic anhydrase IX (CA9), mRNA."
ILMN_1687213	C8ORF13	1.991951	0.000254	0.015119	5.116705	Up	"Homo sapiens chromosome 8 open reading frame 13 (C8orf13), mRNA."
ILMN_1741014	SLC28A3	1.491067	0.000264	0.015441	5.092757	Up	"Homo sapiens solute carrier family 28 (sodium-coupled nucleoside transporter), member 3 (SLC28A3), mRNA."
ILMN_1704353	IGSF3	1.29506	0.000265	0.015447	5.091062	Up	"Homo sapiens immunoglobulin superfamily, member 3 (IGSF3), transcript variant 1, mRNA."
ILMN_1700248	WDR86	1.419424	0.000267	0.015471	5.087159	Up	"Homo sapiens WD repeat domain 86 (WDR86), mRNA."
ILMN_1720511	LRRN1	1.264958	0.000277	0.015973	5.063562	Up	"Homo sapiens leucine rich repeat neuronal 1 (LRRN1), mRNA."
ILMN_1709674	GFPT2	1.621471	0.000284	0.016157	5.050281	Up	"Homo sapiens glutamine-fructose-6-phosphate transaminase 2 (GFPT2), mRNA."
ILMN_2197128	OSR1	2.308353	0.000284	0.016157	5.048689	Up	"Homo sapiens odd-skipped related 1 (Drosophila) (OSR1), mRNA."
ILMN_1721541	WIF1	3.073334	0.000285	0.016157	5.047279	Up	"Homo sapiens WNT inhibitory factor 1 (WIF1), mRNA."
ILMN_1671318	FRAS1	1.980915	0.000286	0.016194	5.044504	Up	"Homo sapiens Fraser syndrome 1 (FRAS1), transcript variant 2, mRNA."
ILMN_1654966	SCARA3	1.599718	0.000292	0.016384	5.032542	Up	"Homo sapiens scavenger receptor class A, member 3 (SCARA3), transcript variant 2, mRNA."
ILMN_1710553	TMEM151A	1.805513	0.000297	0.016584	5.022446	Up	"Homo sapiens transmembrane protein 151A (TMEM151A), mRNA."
ILMN_1771126	RORC	1.807382	0.000303	0.016796	5.009953	Up	"Homo sapiens RAR-related orphan receptor C (RORC), transcript variant 2, mRNA."
ILMN_2402600	GLIS3	1.347697	0.000305	0.016881	5.006232	Up	"Homo sapiens GLIS family zinc finger 3 (GLIS3), transcript variant 2, mRNA."
ILMN_2387105	OGN	2.73285	0.000327	0.017556	4.965867	Up	"Homo sapiens osteoglycin (OGN), transcript variant 3, mRNA."
ILMN_2234697	BEX1	1.431468	0.000327	0.017556	4.9657	Up	"Homo sapiens brain expressed, X-linked 1 (BEX1), mRNA."
ILMN_1663042	SDC4	1.271488	0.000327	0.017562	4.964802	Up	"Homo sapiens syndecan 4 (SDC4), mRNA."
ILMN_1735762	NPNT	1.339389	0.000329	0.017654	4.961023	Up	"Homo sapiens nephronectin (NPNT), mRNA."
ILMN_1740842	SALL2	1.420263	0.000331	0.017703	4.958093	Up	"Homo sapiens sal-like 2 (Drosophila) (SALL2), mRNA."
ILMN_1758392	ANKS1B	1.251145	0.000332	0.017711	4.956402	Up	"Homo sapiens ankyrin repeat and sterile alpha motif domain containing 1B (ANKS1B), transcript variant 2, mRNA."
ILMN_2151114	VSNL1	1.274454	0.000343	0.017931	4.937147	Up	"Homo sapiens visinin-like 1 (VSNL1), mRNA."
ILMN_1749379	GALNT9	1.428648	0.00035	0.018155	4.925185	Up	"Homo sapiens UDP-N-acetyl-alpha-D-galactosamine:polypeptide N-acetylgalactosaminyltransferase 9 (GalNAc-T9) (GALNT9), mRNA."
ILMN_1749636	DNAJC22	2.01399	0.00037	0.01889	4.892675	Up	"Homo sapiens DnaJ (Hsp40) homolog, subfamily C, member 22 (DNAJC22), mRNA."

ILMN_1730291	ATP1B1	1.534974	0.000374	0.018968	4.886476	Up	"Homo sapiens ATPase, Na+/K+ transporting, beta 1 polypeptide (ATP1B1), transcript variant 1, mRNA."
ILMN_1766165	SNCA	1.420587	0.000376	0.019072	4.882529	Up	"Homo sapiens synuclein, alpha (non A4 component of amyloid precursor) (SNCA), transcript variant NACP140, mRNA."
ILMN_1706441	UPK3B	4.110486	0.000377	0.019089	4.880553	Up	"Homo sapiens uroplakin 3B (UPK3B), transcript variant 1, mRNA."
ILMN_1795778	P4HA2	1.26851	0.000385	0.019243	4.868946	Up	"Homo sapiens prolyl 4-hydroxylase, alpha polypeptide II (P4HA2), transcript variant 2, mRNA."
ILMN_1802654	GLT8D2	1.594107	0.000385	0.019243	4.868941	Up	"Homo sapiens glycosyltransferase 8 domain containing 2 (GLT8D2), mRNA."
ILMN_1656287	SPOCK2	1.681296	0.000411	0.020037	4.829864	Up	"Homo sapiens sparc/osteonectin, cwcv and kazal-like domains proteoglycan (testican) 2 (SPOCK2), mRNA."
ILMN_1705750	TGM2	1.257944	0.000413	0.020097	4.827494	Up	"Homo sapiens transglutaminase 2 (C polypeptide, protein-glutamine-gamma-glutamyltransferase) (TGM2), transcript variant 1, mRNA."
ILMN_1741768	TMPRSS3	1.352668	0.000435	0.020845	4.796282	Up	"Homo sapiens transmembrane protease, serine 3 (TMPRSS3), transcript variant D, mRNA."
ILMN_1775542	FAIM3	1.265841	0.000439	0.020956	4.790808	Up	"Homo sapiens Fas apoptotic inhibitory molecule 3 (FAIM3), mRNA."
ILMN_1790227	IGFL2	1.93123	0.00046	0.021635	4.764347	Up	"Homo sapiens IGF-like family member 2 (IGFL2), mRNA."
ILMN_1796069	CBLN2	1.684904	0.000481	0.022352	4.73801	Up	"Homo sapiens cerebellin 2 precursor (CBLN2), mRNA."
ILMN_2388190	SCEL	1.329085	0.000501	0.023039	4.714057	Up	"Homo sapiens sciellin (SCEL), transcript variant 1, mRNA."
ILMN_1731044	KCTD14	1.715081	0.000506	0.023153	4.708713	Up	"Homo sapiens potassium channel tetramerisation domain containing 14 (KCTD14), mRNA."
ILMN_2062701	GAS1	1.478719	0.000506	0.023153	4.707771	Up	"Homo sapiens growth arrest-specific 1 (GAS1), mRNA."
ILMN_1724941	CDCP1	1.338214	0.00052	0.023478	4.691821	Up	"Homo sapiens CUB domain containing protein 1 (CDCP1), transcript variant 1, mRNA."
ILMN_1696048	C13ORF33	1.307729	0.000531	0.023743	4.680319	Up	"Homo sapiens chromosome 13 open reading frame 33 (C13orf33), mRNA."
ILMN_1704084	CMAH	1.613324	0.000534	0.02384	4.677391	Up	"Homo sapiens cytidine monophosphate-N-acetylneuraminic acid hydroxylase (CMP-N-acetylneuraminate monooxygenase) pseudogene (CMAH), transcript variant 1, non-coding RNA."
ILMN_2077905	PTGFRN	1.549865	0.000544	0.02412	4.666526	Up	"Homo sapiens prostaglandin F2 receptor negative regulator (PTGFRN), mRNA."
ILMN_1690223	CNTNAP2	1.479376	0.000547	0.024168	4.662895	Up	"Homo sapiens contactin associated protein-like 2 (CNTNAP2), mRNA."
ILMN_3240308	LOC100133511	2.652644	0.00057	0.024903	4.638502	Up	"PREDICTED: Homo sapiens similar to complement component C3 (LOC100133511), partial mRNA."
ILMN_1679299	IGSF1	1.402305	0.000597	0.025669	4.612189	Up	"Homo sapiens immunoglobulin superfamily, member 1 (IGSF1), transcript variant 1, mRNA."
ILMN_1708925	GALNT9	1.408445	0.000597	0.025669	4.611931	Up	"Homo sapiens UDP-N-acetyl-alpha-D-galactosamine:polypeptide N-acetylgalactosaminyltransferase 9 (GalNAc-T9) (GALNT9), mRNA."
ILMN_1812433	HP	3.128971	0.000608	0.025913	4.601911	Up	"Homo sapiens haptoglobin (HP), mRNA."
ILMN_1685174	CPB1	1.45012	0.000611	0.025964	4.599044	Up	"Homo sapiens carboxypeptidase B1 (tissue) (CPB1), mRNA."
ILMN_3191541	LOC100129165	1.626132	0.000631	0.026429	4.580026	Up	"PREDICTED: Homo sapiens similar to Phosphotidylinositol phosphatase PTPRQ precursor (Receptor-type tyrosine-protein phosphatase Q) (PTP-RQ) (LOC100129165), mRNA."
ILMN_1734596	TC2N	1.24525	0.000638	0.026619	4.573857	Up	"Homo sapiens tandem C2 domains, nuclear (TC2N), mRNA."
ILMN_1709659	TMEM151A	1.566546	0.000655	0.027145	4.558509	Up	"Homo sapiens transmembrane protein 151A (TMEM151A), mRNA."
ILMN_1723123	FGFR3	1.270128	0.000661	0.027315	4.553643	Up	"Homo sapiens fibroblast growth factor receptor 3 (achondroplasia, thanatophoric dwarfism) (FGFR3), transcript variant 2, mRNA."
ILMN_3242586	RHOU	1.318308	0.000685	0.027883	4.53311	Up	"Homo sapiens ras homolog gene family, member U (RHOU), mRNA."
ILMN_1722948	LOC652495	1.635189	0.000721	0.028861	4.503656	Up	"PREDICTED: Homo sapiens similar to Ig gamma-3 chain C region, membrane-bound form (LOC652495), mRNA."

ILMN_1704985	CYP27A1	1.248954	0.000738	0.029246	4.48968	Up	"Homo sapiens cytochrome P450, family 27, subfamily A, polypeptide 1 (CYP27A1), nuclear gene encoding mitochondrial protein, mRNA."
ILMN_1656927	SEMA5A	1.354999	0.000739	0.029246	4.489549	Up	"Homo sapiens sema domain, seven thrombospondin repeats (type 1 and type 1-like), transmembrane domain (TM) and short cytoplasmic domain, (semaphorin) 5A (SEMA5A), mRNA."
ILMN_2134974	RAB38	1.86362	0.00075	0.029547	4.480814	Up	"Homo sapiens RAB38, member RAS oncogene family (RAB38), mRNA."
ILMN_1776395	ARMC4	1.418816	0.000751	0.029547	4.480085	Up	"Homo sapiens armadillo repeat containing 4 (ARMC4), mRNA."
ILMN_1711888	COBL	2.168283	0.000765	0.029862	4.469499	Up	"Homo sapiens cordon-bleu homolog (mouse) (COBL), mRNA."
ILMN_1703572	PCDH20	2.137662	0.000793	0.030595	4.449063	Up	"Homo sapiens protocadherin 20 (PCDH20), mRNA."
ILMN_1874323	HS.562504	1.307697	0.00081	0.030997	4.436377	Up	"PM3-DT0037-231299-001-e06 DT0037 Homo sapiens cDNA, mRNA sequence"
ILMN_1837428	HS.25318	1.752801	0.000816	0.031129	4.432476	Up	Homo sapiens clone 25194 mRNA sequence
ILMN_1749324	DPYS	2.035353	0.00083	0.031557	4.423028	Up	"Homo sapiens dihydropyrimidinase (DPYS), mRNA."
ILMN_2299450	P2RX2	1.263062	0.000845	0.031853	4.412226	Up	"Homo sapiens purinergic receptor P2X, ligand-gated ion channel, 2 (P2RX2), transcript variant 1, mRNA."
ILMN_1694588	C4BPB	1.95841	0.000852	0.032017	4.407934	Up	"Homo sapiens complement component 4 binding protein, beta (C4BPB), transcript variant 3, mRNA."
ILMN_2094875	ABCB1	1.746486	0.000853	0.032025	4.407337	Up	"Homo sapiens ATP-binding cassette, sub-family B (MDR/TAP), member 1 (ABCB1), mRNA."
ILMN_2115991	TNNT1	1.47691	0.000872	0.032409	4.394768	Up	"Homo sapiens troponin T type 1 (skeletal, slow) (TNNT1), mRNA."
ILMN_1704868	LOC648570	1.292761	0.000877	0.032508	4.391172	Up	"PREDICTED: Homo sapiens hypothetical protein LOC648570 (LOC648570), mRNA."
ILMN_1767113	AOX1	1.551246	0.000893	0.032825	4.380757	Up	"Homo sapiens aldehyde oxidase 1 (AOX1), mRNA."
ILMN_1705231	SLCO2A1	1.741187	0.000918	0.033284	4.365411	Up	"Homo sapiens solute carrier organic anion transporter family, member 2A1 (SLCO2A1), mRNA."
ILMN_2139125	LRFN5	1.336329	0.000933	0.033591	4.356272	Up	"Homo sapiens leucine rich repeat and fibronectin type III domain containing 5 (LRFN5), mRNA."
ILMN_2053345	KCNT2	1.373097	0.000949	0.033847	4.346584	Up	"Homo sapiens potassium channel, subfamily T, member 2 (KCNT2), mRNA."
ILMN_2386973	PKP2	1.476597	0.000955	0.033902	4.342672	Up	"Homo sapiens plakophilin 2 (PKP2), transcript variant 2a, mRNA."
ILMN_1677198	C1R	1.322053	0.000956	0.033902	4.342044	Up	"Homo sapiens complement component 1, r subcomponent (C1R), mRNA."
ILMN_2096784	TFAP2C	1.391506	0.001	0.034782	4.316566	Up	"Homo sapiens transcription factor AP-2 gamma (activating enhancer binding protein 2 gamma) (TFAP2C), mRNA."
ILMN_2124241	MUM1L1	1.407985	0.001021	0.035212	4.305039	Up	"Homo sapiens melanoma associated antigen (mutated) 1-like 1 (MUM1L1), mRNA."
ILMN_1740269	WNT2B	2.117437	0.001027	0.035307	4.30187	Up	"Homo sapiens wingless-type MMTV integration site family, member 2B (WNT2B), transcript variant WNT-2B2, mRNA."
ILMN_1743130	PTGFRN	1.566121	0.001037	0.03547	4.296202	Up	"Homo sapiens prostaglandin F2 receptor negative regulator (PTGFRN), mRNA."
ILMN_1750678	TIMD4	3.497012	0.00104	0.035493	4.294802	Up	"Homo sapiens T-cell immunoglobulin and mucin domain containing 4 (TIMD4), mRNA."
ILMN_1696590	CHST4	1.773436	0.001049	0.035727	4.289443	Up	"Homo sapiens carbohydrate (N-acetylglucosamine 6-O) sulfotransferase 4 (CHST4), mRNA."
ILMN_1792682	MCTP2	1.298758	0.001059	0.035825	4.284257	Up	"Homo sapiens multiple C2 domains, transmembrane 2 (MCTP2), mRNA."
ILMN_1769911	SLC38A1	1.589394	0.00107	0.035985	4.278507	Up	"Homo sapiens solute carrier family 38, member 1 (SLC38A1), transcript variant 1, mRNA."
ILMN_1710740	C2	1.554703	0.001098	0.036451	4.263656	Up	"Homo sapiens complement component 2 (C2), mRNA."
ILMN_1740717	ADH1C	1.249423	0.001153	0.037422	4.236413	Up	"Homo sapiens alcohol dehydrogenase 1C (class I), gamma polypeptide (ADH1C), mRNA."
ILMN_1660114	MMRN1	2.848493	0.001187	0.038099	4.219679	Up	"Homo sapiens multimerin 1 (MMRN1), mRNA."
ILMN_1685540	SHRM	1.464773	0.001222	0.038746	4.203502	Up	"Homo sapiens shroom (SHRM), mRNA."
ILMN_1746376	SCARA3	1.447807	0.001287	0.039662	4.174294	Up	"Homo sapiens scavenger receptor class A, member 3 (SCARA3), transcript variant 2, mRNA."
ILMN_3242174	LOC652900	1.274115	0.001463	0.042718	4.102546	Up	"PREDICTED: Homo sapiens similar to SEZ6L2 protein (LOC652900), mRNA."

ILMN_1695299	PDLIM3	1.729421	0.001716	0.047074	4.013762	Up	"Homo sapiens PDZ and LIM domain 3 (PDLIM3), mRNA."
ILMN_2230025	PDLIM3	1.74178	0.001787	0.048243	3.991173	Up	"Homo sapiens PDZ and LIM domain 3 (PDLIM3), mRNA."
ILMN_1740996	CA3	2.347984	0.001808	0.048358	3.984823	Up	"Homo sapiens carbonic anhydrase III, muscle specific (CA3), mRNA."
ILMN_1785570	SUSD3	1.321221	0.001894	0.049692	3.958893	Up	"Homo sapiens sushi domain containing 3 (SUSD3), mRNA."
ILMN_1757736	IRX5	-2.98534	6.18E-13	9.75E-09	-31.6297	Down	"Homo sapiens iroquois homeobox 5 (IRX5), mRNA."
ILMN_1718285	HOXC8	-4.66097	8.9E-13	1.05E-08	-30.6717	Down	"Homo sapiens homeobox C8 (HOXC8), mRNA."
ILMN_1671800	LOC643911	-3.76911	2.59E-11	2.01E-07	-23.0604	Down	"PREDICTED: Homo sapiens hypothetical LOC643911 (LOC643911), mRNA."
ILMN_3250268	LOC643911	-2.94473	3.36E-11	2.01E-07	-22.5585	Down	"PREDICTED: Homo sapiens hCG1815491 (LOC643911), miscRNA."
ILMN_2140559	IRX5	-2.2691	5.94E-11	2.96E-07	-21.4878	Down	"Homo sapiens iroquois homeobox protein 5 (IRX5), mRNA."
ILMN_1721580	TBX15	-3.45121	1.27E-10	4.99E-07	-20.1452	Down	"Homo sapiens T-box 15 (TBX15), mRNA."
ILMN_1718898	HOXC9	-2.96904	1.47E-10	5.34E-07	-19.8853	Down	"Homo sapiens homeobox C9 (HOXC9), mRNA."
ILMN_2230117	AVP	-2.47202	1.58E-10	5.34E-07	-19.7649	Down	"Homo sapiens arginine vasopressin (neurophysin II, antidiuretic hormone, diabetes insipidus, neurohypophyseal) (AVP), mRNA."
ILMN_2198413	MYEOV	-2.88849	4.19E-09	7.34E-06	-14.8889	Down	"Homo sapiens myeloma overexpressed (in a subset of t(11;14) positive multiple myelomas) (MYEOV), mRNA."
ILMN_1687220	DMRT3	-1.70565	9.16E-09	1.16E-05	-13.9009	Down	"Homo sapiens doublesex and mab-3 related transcription factor 3 (DMRT3), mRNA."
ILMN_1739582	HOXA9	-2.37001	9.98E-09	1.16E-05	-13.7968	Down	"Homo sapiens homeobox A9 (HOXA9), mRNA."
ILMN_1722809	NRCAM	-3.29045	1.44E-08	1.55E-05	-13.3529	Down	"Homo sapiens neuronal cell adhesion molecule (NRCAM), transcript variant 2, mRNA."
ILMN_1735124	OXT	-2.3634	1.58E-08	1.63E-05	-13.246	Down	"Homo sapiens oxytocin, prepropeptide (OXT), mRNA."
ILMN_1688480	CCND1	-1.81127	2.01E-08	1.98E-05	-12.9707	Down	"Homo sapiens cyclin D1 (CCND1), mRNA."
ILMN_1794492	HOXC6	-1.50453	4.38E-08	3.57E-05	-12.0976	Down	"Homo sapiens homeobox C6 (HOXC6), transcript variant 1, mRNA."
ILMN_1811468	IRX3	-2.91204	5.66E-08	4.3E-05	-11.8229	Down	"Homo sapiens iroquois homeobox 3 (IRX3), mRNA."
ILMN_1904578	HS.537002	-1.95259	6.36E-08	4.63E-05	-11.6996	Down	Homo sapiens cDNA clone IMAGE:4811759
ILMN_1782412	IRX2	-2.83129	6.49E-08	4.66E-05	-11.6776	Down	"Homo sapiens iroquois homeobox 2 (IRX2), mRNA."
ILMN_2411236	NRCAM	-3.7395	7.02E-08	4.89E-05	-11.5957	Down	"Homo sapiens neuronal cell adhesion molecule (NRCAM), transcript variant 2, mRNA."
ILMN_1678191	GDF10	-2.66863	2.15E-07	0.000113	-10.4763	Down	"Homo sapiens growth differentiation factor 10 (GDF10), mRNA."
ILMN_1716309	EGFL6	-4.53026	2.3E-07	0.000119	-10.4108	Down	"Homo sapiens EGF-like-domain, multiple 6 (EGFL6), mRNA."
ILMN_1715684	LAMB3	-2.69733	2.49E-07	0.000124	-10.3323	Down	"Homo sapiens laminin, beta 3 (LAMB3), transcript variant 1, mRNA."
ILMN_1710544	PCDH7	-1.69014	3.84E-07	0.000175	-9.92906	Down	"Homo sapiens protocadherin 7 (PCDH7), transcript variant a, mRNA."
ILMN_1807925	GNG2	-0.96213	4.96E-07	0.000217	-9.69551	Down	"Homo sapiens guanine nucleotide binding protein (G protein), gamma 2 (GNG2), mRNA."
ILMN_1739496	PRRX1	-1.16402	5.26E-07	0.000228	-9.64389	Down	"Homo sapiens paired related homeobox 1 (PRRX1), transcript variant pmx-1a, mRNA."
ILMN_1812702	SIX1	-2.12845	5.29E-07	0.000228	-9.63794	Down	"Homo sapiens SIX homeobox 1 (SIX1), mRNA."
ILMN_1753789	TNN	-1.46013	5.37E-07	0.000229	-9.62418	Down	"Homo sapiens tenascin N (TNN), mRNA."
ILMN_1790810	HCN4	-0.86295	6.47E-07	0.00026	-9.45809	Down	"Homo sapiens hyperpolarization activated cyclic nucleotide-gated potassium channel 4 (HCN4), mRNA."
ILMN_1782558	XG	-2.46833	6.49E-07	0.00026	-9.45563	Down	"Homo sapiens Xg blood group (XG), mRNA."
ILMN_1806862	FLJ35282	-1.85292	1.18E-06	0.000389	-8.94157	Down	"PREDICTED: Homo sapiens similar to mariner transposase (FLJ35282), mRNA."
ILMN_2204576	HSD17B13	-2.32345	1.28E-06	0.000413	-8.87267	Down	"Homo sapiens hydroxysteroid (17-beta) dehydrogenase 13 (HSD17B13), mRNA."
ILMN_1731862	ITIH5	-1.73013	1.39E-06	0.000439	-8.80212	Down	"Homo sapiens inter-alpha (globulin) inhibitor H5 (ITIH5), transcript variant 1, mRNA."

ILMN_1768940	COL15A1	-1.07886	1.71E-06	0.000528	-8.62968	Down	"Homo sapiens collagen, type XV, alpha 1 (COL15A1), mRNA."
ILMN_1697460	REEP6	-1.80703	1.88E-06	0.000559	-8.55043	Down	"Homo sapiens receptor accessory protein 6 (REEP6), mRNA."
ILMN_3239031	SYNC	-0.89577	2E-06	0.00058	-8.49811	Down	"Homo sapiens syncoilin, intermediate filament protein (SYNC), transcript variant 1, mRNA."
ILMN_1709153	PRR16	-0.94468	2.17E-06	0.0006	-8.4347	Down	"Homo sapiens proline rich 16 (PRR16), mRNA."
ILMN_1776888	RHOXF1	-1.47514	2.32E-06	0.000631	-8.37862	Down	"Homo sapiens Rhox homeobox family, member 1 (RHOXF1), mRNA."
ILMN_1759910	SERPINA5	-2.25342	2.82E-06	0.000738	-8.2215	Down	"Homo sapiens serpin peptidase inhibitor, clade A (alpha-1 antiproteinase, antitrypsin), member 5 (SERPINA5), mRNA."
ILMN_1660871	NEK6	-0.81536	3.02E-06	0.000778	-8.16721	Down	"Homo sapiens NIMA (never in mitosis gene a)-related kinase 6 (NEK6), mRNA."
ILMN_1700081	FST	-1.34949	3.1E-06	0.000788	-8.14849	Down	"Homo sapiens follistatin (FST), transcript variant FST344, mRNA."
ILMN_1689336	HOXA10	-2.72701	3.36E-06	0.000832	-8.08515	Down	"Homo sapiens homeobox A10 (HOXA10), transcript variant 1, mRNA."
ILMN_1801226	DOCK6	-1.38028	3.68E-06	0.000876	-8.01329	Down	"Homo sapiens dedicator of cytokinesis 6 (DOCK6), mRNA."
ILMN_2057479	EGFL6	-4.20941	3.72E-06	0.000876	-8.00407	Down	"Homo sapiens EGF-like-domain, multiple 6 (EGFL6), mRNA."
ILMN_1680367	C10ORF90	-1.54806	4.08E-06	0.000933	-7.93248	Down	"Homo sapiens chromosome 10 open reading frame 90 (C10orf90), mRNA."
ILMN_1683844	HSD17B13	-2.31547	4.5E-06	0.000992	-7.85644	Down	"Homo sapiens hydroxysteroid (17-beta) dehydrogenase 13 (HSD17B13), mRNA."
ILMN_1714988	HOXD8	-1.19742	4.51E-06	0.000992	-7.85554	Down	"Homo sapiens homeobox D8 (HOXD8), mRNA."
ILMN_1713764	LOC440928	-1.73055	4.64E-06	0.001017	-7.83283	Down	"PREDICTED: Homo sapiens hypothetical LOC440928 (LOC440928), mRNA."
ILMN_3241729	EMX2OS	-1.14684	4.8E-06	0.001046	-7.80766	Down	"Homo sapiens EMX2 opposite strand (non-protein coding) (EMX2OS), non-coding RNA."
ILMN_1685714	INHBB	-1.70646	5.28E-06	0.001116	-7.73379	Down	"Homo sapiens inhibin, beta B (activin AB beta polypeptide) (INHBB), mRNA."
ILMN_2390919	FBLN2	-0.81798	5.47E-06	0.001151	-7.70691	Down	"Homo sapiens fibulin 2 (FBLN2), transcript variant 2, mRNA."
ILMN_2038775	TUBB2A	-1.66725	5.57E-06	0.001167	-7.69276	Down	"Homo sapiens tubulin, beta 2A (TUBB2A), mRNA."
ILMN_1724994	COL4A2	-1.32593	5.68E-06	0.001179	-7.67812	Down	"Homo sapiens collagen, type IV, alpha 2 (COL4A2), mRNA."
ILMN_1672908	TWIST1	-1.97748	5.75E-06	0.001186	-7.66873	Down	"Homo sapiens twist homolog 1 (Drosophila) (TWIST1), mRNA."
ILMN_1653028	COL4A1	-0.87182	5.91E-06	0.0012	-7.64853	Down	"Homo sapiens collagen, type IV, alpha 1 (COL4A1), mRNA."
ILMN_1726589	CD248	-1.52098	6.13E-06	0.001231	-7.62072	Down	"Homo sapiens CD248 molecule, endosialin (CD248), mRNA."
ILMN_2075892	LRP5	-1.43593	6.24E-06	0.001236	-7.60715	Down	"Homo sapiens low density lipoprotein receptor-related protein 5 (LRP5), mRNA."
ILMN_1663490	ZNF541	-1.42234	6.58E-06	0.001282	-7.56708	Down	"Homo sapiens zinc finger protein 541 (ZNF541), mRNA."
ILMN_2396039	HOXC6	-1.16631	6.63E-06	0.001286	-7.56162	Down	"Homo sapiens homeobox C6 (HOXC6), transcript variant 2, mRNA."
ILMN_1790350	TPRG1	-1.51709	6.85E-06	0.001314	-7.53712	Down	"Homo sapiens tumor protein p63 regulated 1 (TPRG1), mRNA."
ILMN_1812461	WISP2	-1.9995	7E-06	0.001321	-7.52043	Down	"Homo sapiens WNT1 inducible signaling pathway protein 2 (WISP2), mRNA."
ILMN_3199911	LOC100133058	-0.9852	7.03E-06	0.001321	-7.51811	Down	"PREDICTED: Homo sapiens misc_RNA (LOC100133058), miscRNA."
ILMN_1777397	MSX1	-1.66709	8.07E-06	0.001479	-7.4154	Down	"Homo sapiens msh homeobox 1 (MSX1), mRNA."
ILMN_2329679	TPST2	-0.97605	8.97E-06	0.001608	-7.33688	Down	"Homo sapiens tyrosylprotein sulfotransferase 2 (TPST2), transcript variant 1, mRNA."
ILMN_1678170	MME	-1.85544	9.09E-06	0.001616	-7.32759	Down	"Homo sapiens membrane metallo-endopeptidase (MME), transcript variant 1, mRNA."
ILMN_1764714	LOC55908	-2.85613	1.1E-05	0.001865	-7.18542	Down	"Homo sapiens hepatocellular carcinoma-associated gene TD26 (LOC55908), mRNA."
ILMN_1709593	EN1	-1.06609	1.15E-05	0.001903	-7.15574	Down	"Homo sapiens engrailed homeobox 1 (EN1), mRNA."
ILMN_2107991	HABP4	-1.19103	1.16E-05	0.001903	-7.14894	Down	"Homo sapiens hyaluronan binding protein 4 (HABP4), mRNA."

ILMN_2070521	BHMT2	-2.03678	1.32E-05	0.002096	-7.05636	Down	"Homo sapiens betaine-homocysteine methyltransferase 2 (BHMT2), mRNA."
ILMN_1741404	MSC	-1.7556	1.35E-05	0.002126	-7.03852	Down	"Homo sapiens musculin (activated B-cell factor-1) (MSC), mRNA."
ILMN_1652631	GLIPR2	-1.2844	1.36E-05	0.002126	-7.03694	Down	"Homo sapiens GLI pathogenesis-related 2 (GLIPR2), mRNA."
ILMN_1711699	LOC728014	-1.10225	1.36E-05	0.002129	-7.03326	Down	"PREDICTED: Homo sapiens similar to huntingtin interacting protein 1 related (LOC728014), mRNA."
ILMN_1714067	NTRK2	-1.4948	1.37E-05	0.002139	-7.02771	Down	"Homo sapiens neurotrophic tyrosine kinase, receptor, type 2 (NTRK2), transcript variant b, mRNA."
ILMN_1798372	ANXA2P3	-0.78301	1.4E-05	0.002168	-7.01176	Down	"Homo sapiens annexin A2 pseudogene 3 (ANXA2P3), non-coding RNA."
ILMN_1814397	EPB42	-1.13127	1.46E-05	0.002242	-6.98232	Down	"Homo sapiens erythrocyte membrane protein band 4.2 (EPB42), mRNA."
ILMN_1722945	C6ORF52	-0.84037	1.5E-05	0.002277	-6.96673	Down	"PREDICTED: Homo sapiens chromosome 6 open reading frame 52 (C6orf52), mRNA."
ILMN_1737394	LMNA	-0.90427	1.51E-05	0.002291	-6.95996	Down	"Homo sapiens lamin A/C (LMNA), transcript variant 2, mRNA."
ILMN_2306661	UNC13C	-1.23756	1.55E-05	0.00233	-6.94142	Down	"Homo sapiens unc-13 homolog C (C. elegans) (UNC13C), mRNA."
ILMN_2187746	EMX2	-1.94359	1.63E-05	0.002421	-6.90755	Down	"Homo sapiens empty spiracles homeobox 2 (EMX2), mRNA."
ILMN_2049184	DNASE1L3	-2.58347	1.71E-05	0.002525	-6.87098	Down	"Homo sapiens deoxyribonuclease I-like 3 (DNASE1L3), mRNA."
ILMN_1704091	DGAT1	-1.21204	1.81E-05	0.002641	-6.8328	Down	"Homo sapiens diacylglycerol O-acyltransferase homolog 1 (mouse) (DGAT1), mRNA."
ILMN_1729596	INF2	-1.05949	1.9E-05	0.002721	-6.79916	Down	"Homo sapiens inverted formin, FH2 and WH2 domain containing (INF2), transcript variant 3, mRNA."
ILMN_3241657	TMEM204	-0.72852	1.97E-05	0.002812	-6.77187	Down	"Homo sapiens transmembrane protein 204 (TMEM204), mRNA."
ILMN_2062620	NMT2	-1.15819	2.01E-05	0.002844	-6.75985	Down	"Homo sapiens N-myristoyltransferase 2 (NMT2), mRNA."
ILMN_1707232	EBF3	-0.86728	2.14E-05	0.002957	-6.71617	Down	"Homo sapiens early B-cell factor 3 (EBF3), mRNA."
ILMN_1669888	CSTA	-1.13823	2.22E-05	0.002988	-6.68924	Down	"Homo sapiens cystatin A (stefin A) (CSTA), mRNA."
ILMN_1702822	NTRK2	-1.07166	2.23E-05	0.002988	-6.68681	Down	"Homo sapiens neurotrophic tyrosine kinase, receptor, type 2 (NTRK2), transcript variant a, mRNA."
ILMN_2141482	SERPINF1	-0.84126	2.24E-05	0.002998	-6.68254	Down	"Homo sapiens serpin peptidase inhibitor, clade F (alpha-2 antiplasmin, pigment epithelium derived factor), member 1 (SERPINF1), mRNA."
ILMN_1666894	CSPG4	-1.70754	2.43E-05	0.003218	-6.62596	Down	"Homo sapiens chondroitin sulfate proteoglycan 4 (CSPG4), mRNA."
ILMN_3310065	SFTA1P	-1.26485	2.46E-05	0.003227	-6.61984	Down	"Homo sapiens surfactant associated 1 (pseudogene) (SFTA1P), non-coding RNA."
ILMN_1754795	FAT1	-0.76351	2.48E-05	0.003235	-6.6127	Down	"Homo sapiens FAT tumor suppressor homolog 1 (Drosophila) (FAT1), mRNA."
ILMN_1712461	CBLN1	-1.59769	2.54E-05	0.003291	-6.59722	Down	"Homo sapiens cerebellin 1 precursor (CBLN1), mRNA."
ILMN_1698313	CPM	-1.18671	2.57E-05	0.003312	-6.58907	Down	"Homo sapiens carboxypeptidase M (CPM), transcript variant 1, mRNA."
ILMN_1769129	CCL19	-2.3251	2.73E-05	0.003476	-6.54662	Down	"Homo sapiens chemokine (C-C motif) ligand 19 (CCL19), mRNA."
ILMN_1658926	NOTCH3	-1.1822	2.84E-05	0.003561	-6.52091	Down	"Homo sapiens Notch homolog 3 (Drosophila) (NOTCH3), mRNA."
ILMN_1751901	TMEM163	-1.0673	3.03E-05	0.003714	-6.47622	Down	"Homo sapiens transmembrane protein 163 (TMEM163), mRNA."
ILMN_1733841	TCF7L1	-1.00635	3.13E-05	0.003788	-6.45345	Down	"Homo sapiens transcription factor 7-like 1 (T-cell specific, HMG-box) (TCF7L1), mRNA."
ILMN_1767349	ABCB4	-0.80452	3.19E-05	0.00383	-6.44105	Down	"Homo sapiens ATP-binding cassette, sub-family B (MDR/TAP), member 4 (ABCB4), transcript variant C, mRNA."
ILMN_1684357	C14ORF139	-0.91169	3.2E-05	0.00383	-6.43878	Down	"PREDICTED: Homo sapiens chromosome 14 open reading frame 139 (C14orf139), misc RNA."
ILMN_1683470	TMEM139	-1.23539	3.25E-05	0.003868	-6.42946	Down	"Homo sapiens transmembrane protein 139 (TMEM139), mRNA."
ILMN_2129161	LRRC32	-0.81322	3.34E-05	0.003926	-6.41095	Down	"Homo sapiens leucine rich repeat containing 32 (LRRC32), mRNA."
ILMN_1756701	DHRS11	-1.22704	3.52E-05	0.004061	-6.37488	Down	"Homo sapiens dehydrogenase/reductase (SDR family) member 11 (DHRS11), mRNA."

ILMN_1742866	F2R	-0.9017	3.71E-05	0.004208	-6.33946	Down	"Homo sapiens coagulation factor II (thrombin) receptor (F2R), mRNA."
ILMN_1774602	FBLN2	-0.74013	3.88E-05	0.004356	-6.30886	Down	"Homo sapiens fibulin 2 (FBLN2), transcript variant 2, mRNA."
ILMN_1785732	TNFAIP6	-1.68406	3.91E-05	0.004368	-6.30342	Down	"Homo sapiens tumor necrosis factor, alpha-induced protein 6 (TNFAIP6), mRNA."
ILMN_3239775	ODZ4	-1.59437	4.01E-05	0.004447	-6.28664	Down	"Homo sapiens odz, odd Oz/ten-m homolog 4 (Drosophila) (ODZ4), mRNA."
ILMN_2117508	CTHRC1	-1.95139	4.04E-05	0.00445	-6.28149	Down	"Homo sapiens collagen triple helix repeat containing 1 (CTHRC1), mRNA."
ILMN_1793695	ITIH5	-1.97618	4.13E-05	0.004507	-6.26677	Down	"Homo sapiens inter-alpha (globulin) inhibitor H5 (ITIH5), transcript variant 3, mRNA."
ILMN_1715416	NUP188	-0.79836	4.17E-05	0.00452	-6.26138	Down	"Homo sapiens nucleoporin 188kDa (NUP188), mRNA."
ILMN_1738869	GALNT13	-1.13989	4.2E-05	0.004533	-6.25682	Down	"Homo sapiens UDP-N-acetyl-alpha-D-galactosamine:polypeptide N-acetylgalactosaminyltransferase 13 (GalNAc-T13) (GALNT13), mRNA."
ILMN_1731914	SCN4A	-1.51496	4.21E-05	0.004541	-6.25423	Down	"Homo sapiens sodium channel, voltage-gated, type IV, alpha subunit (SCN4A), mRNA."
ILMN_1732538	LBP	-2.69831	4.23E-05	0.004555	-6.25065	Down	"Homo sapiens lipopolysaccharide binding protein (LBP), mRNA."
ILMN_1812867	LOC126767	-0.81237	4.53E-05	0.004821	-6.20529	Down	"PREDICTED: Homo sapiens similar to arylacetamide deacetylase, transcript variant 2 (LOC126767), mRNA."
ILMN_1729287	NMUR1	-1.36466	4.69E-05	0.004942	-6.18296	Down	"Homo sapiens neuromedin U receptor 1 (NMUR1), mRNA."
ILMN_1801516	GPC1	-1.57157	4.81E-05	0.005037	-6.16602	Down	"Homo sapiens glypican 1 (GPC1), mRNA."
ILMN_1886487	HS.559234	-1.1233	4.83E-05	0.005048	-6.16309	Down	"UI-H-BI1-adn-f-06-0-UI.s1 NCI_CGAP_Sub3 Homo sapiens cDNA clone IMAGE:2717339 3, mRNA sequence"
ILMN_1874678	HS.26579	-0.94618	4.91E-05	0.005088	-6.15211	Down	"AGENCOURT_6808794 NIH_MGC_71 Homo sapiens cDNA clone IMAGE:5784515 5, mRNA sequence"
ILMN_1751439	EMX2	-1.31481	4.99E-05	0.005159	-6.14142	Down	"Homo sapiens empty spiracles homeobox 2 (EMX2), mRNA."
ILMN_1710903	MAPT	-0.92864	5.09E-05	0.005232	-6.12932	Down	"Homo sapiens microtubule-associated protein tau (MAPT), transcript variant 3, mRNA."
ILMN_1792384	HABP4	-1.02189	5.17E-05	0.005279	-6.11912	Down	"Homo sapiens hyaluronan binding protein 4 (HABP4), mRNA."
ILMN_1803818	NMNAT2	-1.42967	5.27E-05	0.005329	-6.10625	Down	"Homo sapiens nicotinamide nucleotide adenylyltransferase 2 (NMNAT2), transcript variant 1, mRNA."
ILMN_2357855	NTRK2	-1.08201	5.29E-05	0.005329	-6.10304	Down	"Homo sapiens neurotrophic tyrosine kinase, receptor, type 2 (NTRK2), transcript variant d, mRNA."
ILMN_1670383	PCDH7	-1.03034	5.31E-05	0.005334	-6.10104	Down	"Homo sapiens protocadherin 7 (PCDH7), transcript variant c, mRNA."
ILMN_1706960	KIAA1217	-0.80426	5.36E-05	0.005358	-6.09445	Down	"Homo sapiens KIAA1217 (KIAA1217), transcript variant 2, mRNA."
ILMN_1811765	ADAMTS2	-0.91855	5.46E-05	0.005441	-6.08248	Down	"Homo sapiens ADAM metallopeptidase with thrombospondin type 1 motif, 2 (ADAMTS2), transcript variant 2, mRNA."
ILMN_1729831	LOC388814	-1.79075	5.95E-05	0.005829	-6.0264	Down	"PREDICTED: Homo sapiens hypothetical LOC388814 (LOC388814), mRNA."
ILMN_1797974	AIG1	-0.87203	5.99E-05	0.005848	-6.02226	Down	"Homo sapiens androgen-induced 1 (AIG1), mRNA."
ILMN_3243924	P2RX6	-2.50443	6.04E-05	0.00587	-6.01647	Down	"Homo sapiens purinergic receptor P2X, ligand-gated ion channel, 6 (P2RX6), transcript variant 1, mRNA."
ILMN_1755974	ALDOC	-1.87486	6.13E-05	0.005904	-6.0068	Down	"Homo sapiens aldolase C, fructose-bisphosphate (ALDOC), mRNA."
ILMN_1795679	STMN2	-3.62531	6.14E-05	0.005904	-6.00603	Down	"Homo sapiens stathmin-like 2 (STMN2), mRNA."
ILMN_1702775	LRP5	-1.34727	6.32E-05	0.006028	-5.98724	Down	"Homo sapiens low density lipoprotein receptor-related protein 5 (LRP5), mRNA."
ILMN_1761312	CRHBP	-1.68538	6.69E-05	0.006304	-5.94983	Down	"Homo sapiens corticotropin releasing hormone binding protein (CRHBP), mRNA."
ILMN_3247578	FAT1	-0.97003	6.81E-05	0.006379	-5.93838	Down	"Homo sapiens FAT tumor suppressor homolog 1 (Drosophila) (FAT1), mRNA."
ILMN_1723414	HACL1	-0.81225	6.98E-05	0.006453	-5.92248	Down	"Homo sapiens 2-hydroxyacyl-CoA lyase 1 (HACL1), mRNA."
ILMN_1701603	ALPL	-1.53611	7.01E-05	0.006457	-5.91994	Down	"Homo sapiens alkaline phosphatase, liver/bone/kidney (ALPL), transcript variant 1, mRNA."

ILMN_3246294	LOC728819	-0.83884	7.07E-05	0.006482	-5.91456	Down	"Homo sapiens hCG1645220 (LOC728819), mRNA."
ILMN_2199389	VIPR1	-1.18858	8.03E-05	0.007137	-5.83252	Down	"Homo sapiens vasoactive intestinal peptide receptor 1 (VIPR1), mRNA."
ILMN_1767111	ANO10	-0.76441	8.15E-05	0.007196	-5.8228	Down	"Homo sapiens anoctamin 10 (ANO10), mRNA."
ILMN_1717599	CNTN3	-1.25609	8.33E-05	0.007329	-5.80849	Down	"Homo sapiens contactin 3 (plasmacytoma associated) (CNTN3), mRNA."
ILMN_1755354	YBX2	-1.02406	8.45E-05	0.007374	-5.80006	Down	"Homo sapiens Y box binding protein 2 (YBX2), mRNA."
ILMN_1791067	TESK1	-1.1465	8.69E-05	0.007521	-5.78158	Down	"Homo sapiens testis-specific kinase 1 (TESK1), mRNA."
ILMN_3248347	PRHOXNB	-2.36802	8.87E-05	0.007633	-5.76863	Down	"Homo sapiens parahox cluster neighbor (PRHOXNB), mRNA."
ILMN_1676067	SEMA3G	-2.25108	9.04E-05	0.007742	-5.75693	Down	"Homo sapiens sema domain, immunoglobulin domain (Ig), short basic domain, secreted, (semaphorin) 3G (SEMA3G), mRNA."
ILMN_1722073	MLXIPL	-1.0962	9.05E-05	0.007742	-5.75612	Down	"Homo sapiens MLX interacting protein-like (MLXIPL), transcript variant 2, mRNA."
ILMN_2317581	SHANK3	-0.88674	9.1E-05	0.007775	-5.75226	Down	"Homo sapiens SH3 and multiple ankyrin repeat domains 3 (SHANK3), mRNA."
ILMN_1710284	HES1	-1.33895	9.22E-05	0.007815	-5.74419	Down	"Homo sapiens hairy and enhancer of split 1, (Drosophila) (HES1), mRNA."
ILMN_1704253	C6ORF106	-0.96527	9.76E-05	0.008187	-5.70805	Down	"Homo sapiens chromosome 6 open reading frame 106 (C6orf106), transcript variant 1, mRNA."
ILMN_1699665	CLIC6	-2.53342	9.84E-05	0.008238	-5.703	Down	"Homo sapiens chloride intracellular channel 6 (CLIC6), mRNA."
ILMN_3190833	CCRL2	-1.10043	0.000101	0.008375	-5.68695	Down	"Homo sapiens chemokine (C-C motif) receptor-like 2 (CCRL2), transcript variant 2, mRNA."
ILMN_1707337	MSTO1	-1.09961	0.000104	0.008494	-5.67031	Down	"Homo sapiens misato homolog 1 (Drosophila) (MSTO1), mRNA."
ILMN_1813043	BHMT2	-1.83449	0.000104	0.008524	-5.667	Down	"Homo sapiens betaine-homocysteine methyltransferase 2 (BHMT2), mRNA."
ILMN_1764729	JAG2	-0.75627	0.000105	0.0086	-5.65886	Down	"Homo sapiens jagged 2 (JAG2), transcript variant 1, mRNA."
ILMN_1696749	LMNA	-0.82584	0.000106	0.0086	-5.65702	Down	"Homo sapiens lamin A/C (LMNA), transcript variant 2, mRNA."
ILMN_1738558	RGS20	-1.27237	0.000109	0.00878	-5.6396	Down	"Homo sapiens regulator of G-protein signaling 20 (RGS20), transcript variant 1, mRNA."
ILMN_1738657	SATB2	-0.99995	0.000113	0.009014	-5.61537	Down	"Homo sapiens SATB homeobox 2 (SATB2), mRNA."
ILMN_1792571	ARHGEF15	-1.21232	0.000114	0.009027	-5.6115	Down	"Homo sapiens Rho guanine nucleotide exchange factor (GEF) 15 (ARHGEF15), mRNA."
ILMN_1738749	MAST3	-0.76235	0.000115	0.009092	-5.60487	Down	"Homo sapiens microtubule associated serine/threonine kinase 3 (MAST3), mRNA."
ILMN_3249281	HOXA11AS	-0.74242	0.000116	0.009187	-5.59664	Down	"Homo sapiens HOXA11 antisense RNA (non-protein coding) (HOXA11AS), antisense RNA."
ILMN_1707310	MGLL	-1.34445	0.00012	0.009392	-5.57821	Down	"Homo sapiens monoglyceride lipase (MGLL), transcript variant 2, mRNA."
ILMN_1669788	NUDT14	-0.94395	0.000121	0.00949	-5.57071	Down	"Homo sapiens nudix (nucleoside diphosphate linked moiety X)-type motif 14 (NUDT14), mRNA."
ILMN_1703650	TNIP1	-1.00328	0.000123	0.009546	-5.5629	Down	"Homo sapiens TNFAIP3 interacting protein 1 (TNIP1), mRNA."
ILMN_1666022	TNFRSF10D	-0.83951	0.000123	0.009555	-5.56123	Down	"Homo sapiens tumor necrosis factor receptor superfamily, member 10d, decoy with truncated death domain (TNFRSF10D), mRNA."
ILMN_1740523	KTN1	-0.84805	0.000125	0.009628	-5.55444	Down	"Homo sapiens kinectin 1 (kinesin receptor) (KTN1), transcript variant 1, mRNA."
ILMN_2360401	LFNG	-1.33282	0.000125	0.009631	-5.55321	Down	"Homo sapiens LFNG O-fucosylpeptide 3-beta-N-acetylglucosaminyltransferase (LFNG), transcript variant 2, mRNA."
ILMN_2112744	P2RX6P	-1.30056	0.000126	0.009704	-5.54547	Down	"Homo sapiens purinergic receptor P2X, ligand-gated ion channel, 6 pseudogene (P2RX6P), non-coding RNA."
ILMN_2179083	LOXL4	-0.9091	0.000127	0.00977	-5.54022	Down	"Homo sapiens lysyl oxidase-like 4 (LOXL4), mRNA."
ILMN_1741727	QPCT	-1.1069	0.000134	0.010114	-5.50957	Down	"Homo sapiens glutaminyl-peptide cyclotransferase (QPCT), mRNA."
ILMN_2414568	MEOX1	-1.39981	0.000136	0.010271	-5.49899	Down	"Homo sapiens mesenchyme homeobox 1 (MEOX1), transcript variant 3, mRNA."
ILMN_1789639	FMOD	-1.00438	0.000139	0.010427	-5.48474	Down	"Homo sapiens fibromodulin (FMOD), mRNA."

ILMN_1695506	DKK2	-1.38552	0.000139	0.010427	-5.48464	Down	"Homo sapiens dickkopf homolog 2 (Xenopus laevis) (DKK2), mRNA."
ILMN_1692664	PRR5	-1.04271	0.00014	0.01046	-5.48047	Down	"Homo sapiens proline rich 5 (renal) (PRR5), transcript variant 5, mRNA."
ILMN_1741003	ANXA5	-0.8082	0.000141	0.01046	-5.47679	Down	"Homo sapiens annexin A5 (ANXA5), mRNA."
ILMN_1657708	MGLL	-1.08707	0.000143	0.010547	-5.46811	Down	"Homo sapiens monoglyceride lipase (MGLL), transcript variant 1, mRNA."
ILMN_3244114	DHRS11	-1.4554	0.000143	0.010547	-5.46707	Down	"Homo sapiens dehydrogenase/reductase (SDR family) member 11 (DHRS11), mRNA."
ILMN_2373791	ENPP2	-0.87172	0.000144	0.010555	-5.46395	Down	"Homo sapiens ectonucleotide pyrophosphatase/phosphodiesterase 2 (ENPP2), transcript variant 2, mRNA."
ILMN_1723004	CD72	-0.90272	0.000145	0.010561	-5.4602	Down	"Homo sapiens CD72 molecule (CD72), mRNA."
ILMN_1778956	STS	-1.53224	0.000145	0.010562	-5.45919	Down	"Homo sapiens steroid sulfatase (microsomal), isozyme S (STS), mRNA."
ILMN_1677429	TWIST2	-1.12175	0.000153	0.010937	-5.42621	Down	"Homo sapiens twist homolog 2 (Drosophila) (TWIST2), mRNA."
ILMN_1692938	PSAT1	-1.18242	0.000154	0.010971	-5.4232	Down	"Homo sapiens phosphoserine aminotransferase 1 (PSAT1), transcript variant 2, mRNA."
ILMN_1751785	DMRT2	-2.38096	0.000155	0.010973	-5.41957	Down	"Homo sapiens doublesex and mab-3 related transcription factor 2 (DMRT2), transcript variant 1, mRNA."
ILMN_2335557	ITIH5	-1.43764	0.000157	0.011087	-5.41051	Down	"Homo sapiens inter-alpha (globulin) inhibitor H5 (ITIH5), transcript variant 1, mRNA."
ILMN_1811363	NOVA1	-1.45716	0.000158	0.011132	-5.40696	Down	"Homo sapiens neuro-oncological ventral antigen 1 (NOVA1), transcript variant 3, mRNA."
ILMN_1716507	EPB41L1	-0.92845	0.00016	0.011266	-5.39679	Down	"Homo sapiens erythrocyte membrane protein band 4.1-like 1 (EPB41L1), transcript variant 1, mRNA."
ILMN_1725090	CTHRC1	-1.33182	0.000162	0.011304	-5.38925	Down	"Homo sapiens collagen triple helix repeat containing 1 (CTHRC1), mRNA."
ILMN_2266962	GYPE	-0.79174	0.000165	0.011444	-5.37805	Down	"Homo sapiens glycophorin E (GYPE), transcript variant 2, mRNA."
ILMN_1785218	MTCH2	-0.74228	0.000167	0.011526	-5.37277	Down	"Homo sapiens mitochondrial carrier homolog 2 (C. elegans) (MTCH2), nuclear gene encoding mitochondrial protein, mRNA."
ILMN_1740917	SCNN1B	-0.98499	0.00017	0.01167	-5.35975	Down	"Homo sapiens sodium channel, nonvoltage-gated 1, beta (SCNN1B), mRNA."
ILMN_1751559	C16ORF30	-0.73696	0.000171	0.011693	-5.35727	Down	"Homo sapiens chromosome 16 open reading frame 30 (C16orf30), mRNA."
ILMN_1744381	SERPINE1	-1.22726	0.000172	0.011707	-5.35493	Down	"Homo sapiens serpin peptidase inhibitor, clade E (nexin, plasminogen activator inhibitor type 1), member 1 (SERPINE1), mRNA."
ILMN_2384122	GPR56	-0.94072	0.000172	0.011707	-5.35426	Down	"Homo sapiens G protein-coupled receptor 56 (GPR56), transcript variant 3, mRNA."
ILMN_1720484	CRTAP	-0.83417	0.000175	0.011826	-5.34453	Down	"Homo sapiens cartilage associated protein (CRTAP), mRNA."
ILMN_1797191	KIAA0040	-1.28646	0.000175	0.011837	-5.34163	Down	"Homo sapiens KIAA0040 (KIAA0040), mRNA."
ILMN_2235354	PWWP2	-1.04551	0.000176	0.011837	-5.3413	Down	"Homo sapiens PWWP domain containing 2 (PWWP2), mRNA."
ILMN_1660552	BMP5	-0.77082	0.00018	0.012033	-5.32685	Down	"Homo sapiens bone morphogenetic protein 5 (BMP5), mRNA."
ILMN_1709630	CCDC107	-1.44529	0.000186	0.012325	-5.30526	Down	"Homo sapiens coiled-coil domain containing 107 (CCDC107), mRNA."
ILMN_1690034	SOD3	-1.08141	0.000193	0.012663	-5.28362	Down	"Homo sapiens superoxide dismutase 3, extracellular (SOD3), mRNA."
ILMN_1657145	MEOX1	-1.44907	0.000194	0.012683	-5.2795	Down	"Homo sapiens mesenchyme homeobox 1 (MEOX1), transcript variant 3, mRNA."
ILMN_1677768	POR	-0.77148	0.000195	0.012683	-5.27842	Down	"Homo sapiens P450 (cytochrome) oxidoreductase (POR), mRNA."
ILMN_1758272	MYPN	-0.99276	0.000206	0.013139	-5.24273	Down	"Homo sapiens myopalladin (MYPN), mRNA."
ILMN_1660554	VWA1	-0.92767	0.000215	0.013481	-5.21897	Down	"Homo sapiens von Willebrand factor A domain containing 1 (VWA1), transcript variant 2, mRNA."
ILMN_1699728	BTD	-0.73065	0.000216	0.013552	-5.21393	Down	"Homo sapiens biotinidase (BTD), mRNA."
ILMN_3243644	LOC100132564	-3.22333	0.000219	0.013651	-5.20574	Down	"PREDICTED: Homo sapiens hypothetical protein LOC100132564 (LOC100132564), mRNA."
ILMN_1681520	DGAT2	-1.01235	0.000226	0.013939	-5.18838	Down	"Homo sapiens diacylglycerol O-acyltransferase homolog 2 (mouse) (DGAT2), mRNA."

ILMN_1654072	CX3CL1	-0.83784	0.000228	0.014041	-5.18229	Down	"Homo sapiens chemokine (C-X3-C motif) ligand 1 (CX3CL1), mRNA."
ILMN_2150112	NRN1	-1.29418	0.00023	0.01414	-5.17568	Down	"Homo sapiens neuritin 1 (NRN1), mRNA."
ILMN_1890614	HS.193406	-0.78894	0.000231	0.014152	-5.17438	Down	"Homo sapiens cDNA FLJ34755 fis, clone NHNPC1000034"
ILMN_3177532	CECR4	-0.85792	0.000235	0.0143	-5.16417	Down	"Homo sapiens cat eye syndrome chromosome region, candidate 4 (non-protein coding) (CECR4), transcript variant 2, non-coding RNA."
ILMN_1716465	RBP7	-1.24206	0.000242	0.014633	-5.14562	Down	"Homo sapiens retinol binding protein 7, cellular (RBP7), mRNA."
ILMN_3307729	CXXC5	-0.84078	0.000248	0.014887	-5.13211	Down	"Homo sapiens CXXC finger 5 (CXXC5), mRNA."
ILMN_1678757	BCYRN1	-1.23584	0.000248	0.01491	-5.13043	Down	"Homo sapiens brain cytoplasmic RNA 1 (non-protein coding) (BCYRN1), non-coding RNA."
ILMN_1782939	ALB	-1.33198	0.000251	0.014958	-5.12472	Down	"Homo sapiens albumin (ALB), mRNA."
ILMN_1663454	PKP1	-1.55835	0.000251	0.014958	-5.12469	Down	"Homo sapiens plakophilin 1 (ectodermal dysplasia/skin fragility syndrome) (PKP1), transcript variant 1a, mRNA."
ILMN_1701643	GDPD5	-1.08077	0.000255	0.015144	-5.11419	Down	"Homo sapiens glycerophosphodiester phosphodiesterase domain containing 5 (GDPD5), mRNA."
ILMN_3289779	LOC100131471	-1.14413	0.00026	0.01528	-5.10355	Down	"PREDICTED: Homo sapiens misc_RNA (LOC100131471), miscRNA."
ILMN_2409290	CCPG1	-0.92079	0.00026	0.015289	-5.10243	Down	"Homo sapiens cell cycle progression 1 (CCPG1), transcript variant 2, mRNA."
ILMN_1765274	CAPN11	-1.38463	0.000262	0.015366	-5.09818	Down	"Homo sapiens calpain 11 (CAPN11), mRNA."
ILMN_1719236	CDH5	-0.7663	0.000272	0.015767	-5.07502	Down	"Homo sapiens cadherin 5, type 2, VE-cadherin (vascular epithelium) (CDH5), mRNA."
ILMN_3240765	ANO3	-1.65987	0.000274	0.01584	-5.07008	Down	"Homo sapiens anoctamin 3 (ANO3), mRNA."
ILMN_1813148	TOM1	-0.73066	0.000276	0.015893	-5.06732	Down	"Homo sapiens target of myb1 (chicken) (TOM1), mRNA."
ILMN_1755588	ANKRD47	-0.84968	0.000285	0.016157	-5.04774	Down	"Homo sapiens ankyrin repeat domain 47 (ANKRD47), mRNA."
ILMN_2194688	DKK2	-1.5735	0.000289	0.016276	-5.04004	Down	"Homo sapiens dickkopf homolog 2 (Xenopus laevis) (DKK2), mRNA."
ILMN_1733094	STEAP1	-0.81983	0.000289	0.016305	-5.03818	Down	"Homo sapiens six transmembrane epithelial antigen of the prostate 1 (STEAP1), mRNA."
ILMN_1659371	ADRA2A	-1.59595	0.00029	0.016305	-5.03755	Down	"Homo sapiens adrenergic, alpha-2A-, receptor (ADRA2A), mRNA."
ILMN_2207504	LEP	-0.79311	0.0003	0.016692	-5.01717	Down	"Homo sapiens leptin (obesity homolog, mouse) (LEP), mRNA."
ILMN_2278335	AKR1B15	-1.53666	0.000303	0.016796	-5.01003	Down	"Homo sapiens aldo-keto reductase family 1, member B15 (AKR1B15), mRNA."
ILMN_1657347	PODXL2	-0.87342	0.000309	0.017005	-4.99978	Down	"Homo sapiens podocalyxin-like 2 (PODXL2), mRNA."
ILMN_2207505	LEP	-0.89899	0.000315	0.01722	-4.98731	Down	"Homo sapiens leptin (obesity homolog, mouse) (LEP), mRNA."
ILMN_3199483	LOC100131064	-1.00215	0.000317	0.017257	-4.98407	Down	"PREDICTED: Homo sapiens similar to HSF1 protein (LOC100131064), mRNA."
ILMN_1790859	PLAC9	-0.8784	0.000319	0.017316	-4.97962	Down	"Homo sapiens placenta-specific 9 (PLAC9), mRNA."
ILMN_1709434	VIT	-1.0807	0.000319	0.017316	-4.97932	Down	"Homo sapiens vitrin (VIT), mRNA."
ILMN_1856480	HS.19339	-1.21479	0.000321	0.017362	-4.97705	Down	Homo sapiens cDNA clone IMAGE:5263177
ILMN_2409754	PRR5	-1.0801	0.000325	0.01752	-4.96824	Down	"Homo sapiens proline rich 5 (renal) (PRR5), transcript variant 4, mRNA."
ILMN_1759513	RND3	-0.97105	0.000332	0.017711	-4.95642	Down	"Homo sapiens Rho family GTPase 3 (RND3), mRNA."
ILMN_2174805	CD300LG	-0.87167	0.000334	0.017746	-4.95234	Down	"Homo sapiens CD300 molecule-like family member g (CD300LG), mRNA."
ILMN_1687216	PCBP3	-1.13592	0.000335	0.017746	-4.95147	Down	"Homo sapiens poly(rC) binding protein 3 (PCBP3), mRNA."
ILMN_2174804	CD300LG	-1.06126	0.000335	0.017746	-4.95077	Down	"Homo sapiens CD300 molecule-like family member g (CD300LG), mRNA."
ILMN_1782352	VENTX	-0.97725	0.000336	0.017763	-4.94935	Down	"Homo sapiens VENT homeobox homolog (Xenopus laevis) (VENTX), mRNA."
ILMN_2347592	NMB	-1.35543	0.000338	0.017829	-4.94646	Down	"Homo sapiens neuromedin B (NMB), transcript variant 1, mRNA."
ILMN_1744937	PTPRM	-0.8079	0.00034	0.017878	-4.94214	Down	"Homo sapiens protein tyrosine phosphatase, receptor type, M (PTPRM), mRNA."

ILMN_1742730	TWIST2	-1.36917	0.00034	0.017878	-4.94151	Down	"PREDICTED: Homo sapiens twist homolog 2 (Drosophila), transcript variant 1 (TWIST2), mRNA."
ILMN_1782618	C9ORF16	-1.1215	0.000349	0.018155	-4.92597	Down	"Homo sapiens chromosome 9 open reading frame 16 (C9orf16), mRNA."
ILMN_1715674	ITPK1	-1.49862	0.000356	0.01838	-4.9153	Down	"Homo sapiens inositol 1,3,4-triphosphate 5/6 kinase (ITPK1), mRNA."
ILMN_3308335	RNU6-1	-1.37009	0.00037	0.018891	-4.89135	Down	"Homo sapiens RNA, U6 small nuclear 1 (RNU6-1), small nuclear RNA."
ILMN_1752159	AHNAK	-1.40702	0.00037	0.018891	-4.89135	Down	"Homo sapiens AHNAK nucleoprotein (AHNAK), transcript variant 2, mRNA."
ILMN_1653836	C11ORF41	-0.83733	0.000379	0.019109	-4.87822	Down	"Homo sapiens chromosome 11 open reading frame 41 (C11orf41), mRNA."
ILMN_1743755	LOC441150	-0.79701	0.000383	0.019243	-4.87203	Down	"PREDICTED: Homo sapiens similar to RIKEN cDNA 2310039H08 (LOC441150), mRNA."
ILMN_1815032	SOX7	-0.93932	0.000385	0.019243	-4.86878	Down	"Homo sapiens SRY (sex determining region Y)-box 7 (SOX7), mRNA."
ILMN_1713124	AKR1C3	-1.02381	0.000387	0.019303	-4.866	Down	"Homo sapiens aldo-keto reductase family 1, member C3 (3-alpha hydroxysteroid dehydrogenase, type II) (AKR1C3), mRNA."
ILMN_1794844	THRSP	-0.91851	0.00039	0.019404	-4.86105	Down	"Homo sapiens thyroid hormone responsive (SPOT14 homolog, rat) (THRSP), mRNA."
ILMN_1814719	RBP4	-1.6217	0.000391	0.019411	-4.86005	Down	"Homo sapiens retinol binding protein 4, plasma (RBP4), mRNA."
ILMN_1685275	MCAM	-1.59123	0.000397	0.019598	-4.84988	Down	"Homo sapiens melanoma cell adhesion molecule (MCAM), mRNA."
ILMN_1792748	CPS1	-0.96717	0.000398	0.019598	-4.8492	Down	"Homo sapiens carbamoyl-phosphate synthetase 1, mitochondrial (CPS1), mRNA."
ILMN_1780799	ENPP2	-0.99926	0.0004	0.019665	-4.84638	Down	"Homo sapiens ectonucleotide pyrophosphatase/phosphodiesterase 2 (ENPP2), transcript variant 2, mRNA."
ILMN_1653719	ITGBL1	-0.94615	0.000401	0.019709	-4.84385	Down	"Homo sapiens integrin, beta-like 1 (with EGF-like repeat domains) (ITGBL1), mRNA."
ILMN_1781536	FAH	-1.0043	0.000407	0.019926	-4.83558	Down	"Homo sapiens fumarylacetoacetate hydrolase (fumarylacetoacetase) (FAH), mRNA."
ILMN_1659316	HEPACAM	-0.84999	0.000409	0.019986	-4.83283	Down	"Homo sapiens hepatocyte cell adhesion molecule (HEPACAM), mRNA."
ILMN_1682147	HOOK2	-1.15742	0.000413	0.020108	-4.82658	Down	"Homo sapiens hook homolog 2 (Drosophila) (HOOK2), transcript variant 1, mRNA."
ILMN_1704446	SLC6A10P	-1.07365	0.000414	0.020108	-4.82595	Down	"Homo sapiens solute carrier family 6 (neurotransmitter transporter, creatine), member 10 (pseudogene) (SLC6A10P) on chromosome 16."
ILMN_1654112	PARD6A	-0.97484	0.000433	0.020802	-4.79943	Down	"Homo sapiens par-6 partitioning defective 6 homolog alpha (C. elegans) (PARD6A), transcript variant 2, mRNA."
ILMN_1672091	HOXD4	-0.73402	0.000436	0.020845	-4.79582	Down	"Homo sapiens homeobox D4 (HOXD4), mRNA."
ILMN_3301740	LOC729887	-0.967	0.000439	0.020956	-4.79192	Down	"PREDICTED: Homo sapiens misc_RNA (LOC729887), miscRNA."
ILMN_2196328	POSTN	-0.89339	0.000446	0.021234	-4.78143	Down	"Homo sapiens periostin, osteoblast specific factor (POSTN), mRNA."
ILMN_1772976	BTNL9	-1.36079	0.00045	0.02132	-4.77715	Down	"Homo sapiens butyrophilin-like 9 (BTNL9), mRNA."
ILMN_1690939	ECGF1	-1.31116	0.000452	0.021382	-4.77386	Down	"Homo sapiens endothelial cell growth factor 1 (platelet-derived) (ECGF1), mRNA."
ILMN_3220934	NCRNA00152	-1.02419	0.000453	0.021382	-4.77266	Down	"Homo sapiens non-protein coding RNA 152 (NCRNA00152), transcript variant 1, non-coding RNA."
ILMN_1696846	LOC541471	-0.91292	0.000461	0.021657	-4.76284	Down	"PREDICTED: Homo sapiens hypothetical LOC541471 (LOC541471), misc RNA."
ILMN_1781636	VWA1	-0.98048	0.000475	0.022187	-4.74522	Down	"Homo sapiens von Willebrand factor A domain containing 1 (VWA1), transcript variant 1, mRNA."
ILMN_1695947	SCN4B	-1.05576	0.000477	0.022247	-4.74306	Down	"Homo sapiens sodium channel, voltage-gated, type IV, beta (SCN4B), mRNA."
ILMN_1702226	C21ORF34	-0.7939	0.000479	0.022285	-4.74042	Down	"Homo sapiens chromosome 21 open reading frame 34 (C21orf34), transcript variant 1, mRNA."
ILMN_2412192	CFH	-0.99092	0.000486	0.02254	-4.73196	Down	"Homo sapiens complement factor H (CFH), transcript variant 2, mRNA."
ILMN_1745256	CXXC5	-0.86372	0.000487	0.022557	-4.73064	Down	"Homo sapiens CXXC finger 5 (CXXC5), mRNA."
ILMN_1688886	GPC5	-0.80483	0.000494	0.022767	-4.72212	Down	"Homo sapiens glypican 5 (GPC5), mRNA."

ILMN_2317580	SHANK3	-0.83001	0.000499	0.022974	-4.71627	Down	"Homo sapiens SH3 and multiple ankyrin repeat domains 3 (SHANK3), mRNA."
ILMN_1669747	UNC13B	-0.87052	0.000508	0.023183	-4.70645	Down	"Homo sapiens unc-13 homolog B (C. elegans) (UNC13B), mRNA."
ILMN_2372698	RGN	-0.92834	0.00051	0.023229	-4.70418	Down	"Homo sapiens regucalcin (senescence marker protein-30) (RGN), transcript variant 2, mRNA."
ILMN_1764826	TFE3	-0.75326	0.000512	0.023263	-4.70165	Down	"Homo sapiens transcription factor binding to IGHM enhancer 3 (TFE3), mRNA."
ILMN_1688625	AIM1	-0.73456	0.000513	0.023296	-4.70002	Down	"Homo sapiens absent in melanoma 1 (AIM1), mRNA."
ILMN_1767351	AR	-1.40849	0.000514	0.023296	-4.69859	Down	"Homo sapiens androgen receptor (AR), transcript variant 2, mRNA."
ILMN_1779841	PPP2R1B	-1.16873	0.000526	0.023604	-4.68538	Down	"Homo sapiens protein phosphatase 2 (formerly 2A), regulatory subunit A, beta isoform (PPP2R1B), transcript variant 2, mRNA."
ILMN_1678052	C19ORF24	-0.81649	0.000536	0.023891	-4.67479	Down	"Homo sapiens chromosome 19 open reading frame 24 (C19orf24), mRNA."
ILMN_1805098	PDE1B	-1.12645	0.000543	0.02412	-4.66673	Down	"Homo sapiens phosphodiesterase 1B, calmodulin-dependent (PDE1B), mRNA."
ILMN_1694075	GADD45A	-0.96312	0.000552	0.024326	-4.65804	Down	"Homo sapiens growth arrest and DNA-damage-inducible, alpha (GADD45A), mRNA."
ILMN_1773175	LOC642399	-1.18112	0.000555	0.024397	-4.65468	Down	"PREDICTED: Homo sapiens similar to CG5435-PA (LOC642399), mRNA."
ILMN_1656560	PARM1	-0.87526	0.00057	0.024903	-4.63851	Down	"Homo sapiens prostate androgen-regulated mucin-like protein 1 (PARM1), mRNA."
ILMN_1729161	NOTCH1	-0.81087	0.000578	0.025158	-4.63101	Down	"Homo sapiens Notch homolog 1, translocation-associated (Drosophila) (NOTCH1), mRNA."
ILMN_2395652	PTGFR	-0.93025	0.000583	0.025264	-4.62591	Down	"Homo sapiens prostaglandin F receptor (FP) (PTGFR), transcript variant 1, mRNA."
ILMN_1807503	CPA2	-0.73414	0.000587	0.025405	-4.62161	Down	"Homo sapiens carboxypeptidase A2 (pancreatic) (CPA2), mRNA."
ILMN_1687757	AKR1C4	-0.89335	0.000601	0.025746	-4.60863	Down	"Homo sapiens aldo-keto reductase family 1, member C4 (chlordecone reductase; 3-alpha hydroxysteroid dehydrogenase, type I; dihydrodiol dehydrogenase 4) (AKR1C4), mRNA."
ILMN_1729117	COL5A2	-0.82596	0.000604	0.025812	-4.60558	Down	"Homo sapiens collagen, type V, alpha 2 (COL5A2), mRNA."
ILMN_1675797	EPDR1	-0.75347	0.000604	0.025812	-4.60506	Down	"Homo sapiens ependymin related protein 1 (zebrafish) (EPDR1), mRNA."
ILMN_1772588	C6ORF97	-1.10473	0.000613	0.02604	-4.59684	Down	"Homo sapiens chromosome 6 open reading frame 97 (C6orf97), mRNA."
ILMN_1677963	TMCC1	-0.88984	0.000623	0.026324	-4.58778	Down	"Homo sapiens transmembrane and coiled-coil domain family 1 (TMCC1), transcript variant 1, mRNA."
ILMN_1734184	P76	-1.0586	0.000626	0.026415	-4.58435	Down	"Homo sapiens mannose-6-phosphate protein p76 (P76), mRNA."
ILMN_2049672	TMEM16C	-1.72406	0.000629	0.02642	-4.58227	Down	"Homo sapiens transmembrane protein 16C (TMEM16C), mRNA."
ILMN_1696699	FAM184A	-0.98344	0.000631	0.026429	-4.58047	Down	"Homo sapiens family with sequence similarity 184, member A (FAM184A), transcript variant 1, mRNA."
ILMN_2126038	STMN2	-3.16202	0.000633	0.026488	-4.57824	Down	"Homo sapiens stathmin-like 2 (STMN2), mRNA."
ILMN_1690839	PPAPDC3	-0.72872	0.000641	0.026688	-4.57136	Down	"Homo sapiens phosphatidic acid phosphatase type 2 domain containing 3 (PPAPDC3), mRNA."
ILMN_1716639	LOC646625	-2.15807	0.000646	0.026846	-4.56692	Down	"PREDICTED: Homo sapiens hypothetical LOC646625 (LOC646625), mRNA."
ILMN_1778360	PYGB	-0.86849	0.000651	0.027015	-4.56229	Down	"Homo sapiens phosphorylase, glycogen; brain (PYGB), mRNA."
ILMN_2352097	GPR56	-0.81665	0.000669	0.027489	-4.54624	Down	"Homo sapiens G protein-coupled receptor 56 (GPR56), transcript variant 2, mRNA."
ILMN_1734929	BBOX1	-1.58322	0.000671	0.027529	-4.54489	Down	"Homo sapiens butyrobetaine (gamma), 2-oxoglutarate dioxygenase (gamma-butyrobetaine hydroxylase) 1 (BBOX1), mRNA."
ILMN_1750674	SDSL	-0.77006	0.000677	0.027678	-4.53929	Down	"Homo sapiens serine dehydratase-like (SDSL), mRNA."
ILMN_1789535	DHDDS	-1.18872	0.000687	0.027948	-4.53085	Down	"Homo sapiens dehydrodolichyl diphosphate synthase (DHDDS), transcript variant 2, mRNA."
ILMN_2352953	CPM	-0.79132	0.0007	0.028299	-4.5201	Down	"Homo sapiens carboxypeptidase M (CPM), transcript variant 2, mRNA."
ILMN_1738589	MGLL	-0.77679	0.000713	0.028751	-4.51002	Down	"Homo sapiens monoglyceride lipase (MGLL), transcript variant 1, mRNA."

ILMN_1704376	GLDN	-1.57831	0.000718	0.028861	-4.50533	Down	"Homo sapiens gliomedin (GLDN), mRNA."
ILMN_1667948	SPATA18	-0.9041	0.00072	0.028861	-4.50394	Down	"Homo sapiens spermatogenesis associated 18 homolog (rat) (SPATA18), mRNA."
ILMN_2405185	AOC2	-0.85731	0.000723	0.028923	-4.50172	Down	"Homo sapiens amine oxidase, copper containing 2 (retina-specific) (AOC2), transcript variant 1, mRNA."
ILMN_1651285	BCL6B	-1.09804	0.000729	0.029132	-4.49661	Down	"Homo sapiens B-cell CLL/lymphoma 6, member B (zinc finger protein) (BCL6B), mRNA."
ILMN_1802894	VKORC1L1	-1.09118	0.000733	0.029191	-4.49401	Down	"Homo sapiens vitamin K epoxide reductase complex, subunit 1-like 1 (VKORC1L1), mRNA."
ILMN_2277419	SLC38A10	-0.76426	0.000735	0.029191	-4.49259	Down	"Homo sapiens solute carrier family 38, member 10 (SLC38A10), transcript variant 1, mRNA."
ILMN_1688103	CTNNBIP1	-0.89725	0.000735	0.029191	-4.49257	Down	"Homo sapiens catenin, beta interacting protein 1 (CTNNBIP1), transcript variant 1, mRNA."
ILMN_2396148	HIP1R	-1.1213	0.000735	0.029191	-4.49257	Down	"Homo sapiens huntingtin interacting protein 1 related (HIP1R), mRNA."
ILMN_1655563	KIAA0427	-0.84024	0.000738	0.029246	-4.49014	Down	"Homo sapiens KIAA0427 (KIAA0427), mRNA."
ILMN_1696099	ALDH4A1	-1.21972	0.000745	0.029444	-4.48424	Down	"Homo sapiens aldehyde dehydrogenase 4 family, member A1 (ALDH4A1), nuclear gene encoding mitochondrial protein, transcript variant P5CDhL, mRNA."
ILMN_1733157	THSD1	-0.75584	0.000751	0.029547	-4.48024	Down	"Homo sapiens thrombospondin, type I, domain containing 1 (THSD1), transcript variant 1, mRNA."
ILMN_2405642	DHDDS	-1.17956	0.000755	0.029599	-4.47695	Down	"Homo sapiens dehydrodolichyl diphosphate synthase (DHDDS), transcript variant 1, mRNA."
ILMN_3310351	RNU6-15	-1.29429	0.00077	0.030004	-4.46585	Down	"Homo sapiens RNA, U6 small nuclear 15 (RNU6-15), small nuclear RNA."
ILMN_2098643	FADS3	-1.38206	0.000771	0.030016	-4.4649	Down	"Homo sapiens fatty acid desaturase 3 (FADS3), mRNA."
ILMN_1711157	NOTCH4	-0.86421	0.000775	0.030125	-4.46174	Down	"Homo sapiens Notch homolog 4 (Drosophila) (NOTCH4), mRNA."
ILMN_1707592	RGN	-0.8979	0.000779	0.030182	-4.45916	Down	"Homo sapiens regucalcin (senescence marker protein-30) (RGN), transcript variant 2, mRNA."
ILMN_2399523	JAG2	-0.88448	0.000784	0.030357	-4.45539	Down	"Homo sapiens jagged 2 (JAG2), transcript variant 2, mRNA."
ILMN_1805251	LOC641983	-1.00699	0.000799	0.030755	-4.4442	Down	"PREDICTED: Homo sapiens similar to CDC14 homolog B isoform 2 (LOC641983), mRNA."
ILMN_1750497	GPR109A	-1.45786	0.000803	0.03085	-4.44195	Down	"Homo sapiens G protein-coupled receptor 109A (GPR109A), mRNA."
ILMN_1692123	ELOVL3	-0.82441	0.000811	0.030997	-4.43614	Down	"Homo sapiens elongation of very long chain fatty acids (FEN1/Elo2, SUR4/Elo3, yeast)-like 3 (ELOVL3), mRNA."
ILMN_1859127	HS.190748	-1.11391	0.000811	0.030997	-4.43566	Down	"PREDICTED: Homo sapiens hypothetical LOC388214 (LOC388214), mRNA"
ILMN_1757387	UCHL1	-1.11173	0.000822	0.031305	-4.42842	Down	"Homo sapiens ubiquitin carboxyl-terminal esterase L1 (ubiquitin thiolesterase) (UCHL1), mRNA."
ILMN_2168520	C11ORF70	-0.75458	0.000822	0.031305	-4.42806	Down	"Homo sapiens chromosome 11 open reading frame 70 (C11orf70), mRNA."
ILMN_1861270	HS.374023	-0.78611	0.000834	0.031623	-4.42026	Down	Homo sapiens mRNA; cDNA DKFZp686N1644 (from clone DKFZp686N1644)
ILMN_1768282	SNX21	-0.9381	0.000839	0.031725	-4.41635	Down	"Homo sapiens sorting nexin family member 21 (SNX21), transcript variant 3, mRNA."
ILMN_1657803	CFH	-0.98162	0.000842	0.031812	-4.41432	Down	"Homo sapiens complement factor H (CFH), transcript variant 2, mRNA."
ILMN_1814917	TLE2	-0.84144	0.00085	0.032	-4.40914	Down	"Homo sapiens transducin-like enhancer of split 2 (E(sp1) homolog, Drosophila) (TLE2), mRNA."
ILMN_1714489	CIB2	-0.92858	0.000856	0.032087	-4.4052	Down	"Homo sapiens calcium and integrin binding family member 2 (CIB2), mRNA."
ILMN_2354953	NQO1	-1.01064	0.000872	0.032409	-4.3947	Down	"Homo sapiens NAD(P)H dehydrogenase, quinone 1 (NQO1), transcript variant 1, mRNA."
ILMN_2310814	MAPT	-1.59319	0.000891	0.032774	-4.3821	Down	"Homo sapiens microtubule-associated protein tau (MAPT), transcript variant 4, mRNA."
ILMN_1691574	LOC646064	-0.83773	0.000897	0.032863	-4.37878	Down	"PREDICTED: Homo sapiens hypothetical protein LOC646064 (LOC646064), mRNA."
ILMN_1730670	FSTL3	-1.15379	0.000899	0.032936	-4.37708	Down	"Homo sapiens follistatin-like 3 (secreted glycoprotein) (FSTL3), mRNA."
ILMN_1666594	IRF8	-0.98248	0.000906	0.033119	-4.3726	Down	"Homo sapiens interferon regulatory factor 8 (IRF8), mRNA."
ILMN_1883780	HS.580330	-0.75185	0.00092	0.033332	-4.36415	Down	"DA104205 BRACE3 Homo sapiens cDNA clone BRACE3017487 5, mRNA sequence"

ILMN_1772537	SCTR	-0.89597	0.000936	0.033621	-4.35436	Down	"Homo sapiens secretin receptor (SCTR), mRNA."
ILMN_1810910	CFH	-1.01888	0.000949	0.033847	-4.34656	Down	"Homo sapiens complement factor H (CFH), transcript variant 2, mRNA."
ILMN_1705629	STEAP1	-0.7441	0.000949	0.033847	-4.34635	Down	"PREDICTED: Homo sapiens six transmembrane epithelial antigen of the prostate 1 (STEAP1), mRNA."
ILMN_3204908	LOC100131447	-0.95267	0.00095	0.033847	-4.34588	Down	"PREDICTED: Homo sapiens misc_RNA (LOC100131447), miscRNA."
ILMN_1659649	SGCG	-1.02947	0.000951	0.033847	-4.34536	Down	"Homo sapiens sarcoglycan, gamma (35kDa dystrophin-associated glycoprotein) (SGCG), mRNA."
ILMN_1768556	CASKIN2	-0.72873	0.000956	0.033902	-4.34226	Down	"Homo sapiens CASK interacting protein 2 (CASKIN2), mRNA."
ILMN_1676036	LOC649679	-0.84654	0.000959	0.03394	-4.34051	Down	"PREDICTED: Homo sapiens similar to Tubulin beta-4q chain, transcript variant 2 (LOC649679), mRNA."
ILMN_1724540	CART1	-0.89329	0.00096	0.03394	-4.34013	Down	"Homo sapiens cartilage paired-class homeoprotein 1 (CART1), mRNA."
ILMN_2383383	PIR	-0.82948	0.000967	0.034097	-4.3355	Down	"Homo sapiens pirin (iron-binding nuclear protein) (PIR), transcript variant 2, mRNA."
ILMN_1738116	TMEM119	-0.9688	0.000968	0.034097	-4.3354	Down	"Homo sapiens transmembrane protein 119 (TMEM119), mRNA."
ILMN_2386444	ANGPTL4	-1.42262	0.000984	0.034425	-4.32618	Down	"Homo sapiens angiopoietin-like 4 (ANGPTL4), transcript variant 3, mRNA."
ILMN_1740407	CHSY3	-0.86011	0.000989	0.034549	-4.32288	Down	"Homo sapiens chondroitin sulfate synthase 3 (CHSY3), mRNA."
ILMN_1721968	NOVA1	-0.93741	0.000998	0.034711	-4.31813	Down	"Homo sapiens neuro-oncological ventral antigen 1 (NOVA1), transcript variant 1, mRNA."
ILMN_1813685	RAB7L1	-0.73809	0.001012	0.035005	-4.31003	Down	"Homo sapiens RAB7, member RAS oncogene family-like 1 (RAB7L1), mRNA."
ILMN_1761425	OLFML2A	-0.89208	0.00102	0.035204	-4.30559	Down	"Homo sapiens olfactomedin-like 2A (OLFML2A), mRNA."
ILMN_2244841	ALDH4A1	-1.26122	0.001024	0.035287	-4.30318	Down	"Homo sapiens aldehyde dehydrogenase 4 family, member A1 (ALDH4A1), nuclear gene encoding mitochondrial protein, transcript variant P5CDhL, mRNA."
ILMN_1663032	FNDC4	-0.9097	0.001025	0.035287	-4.30259	Down	"Homo sapiens fibronectin type III domain containing 4 (FNDC4), mRNA."
ILMN_1734445	LOC91461	-0.95298	0.001033	0.03547	-4.29819	Down	"Homo sapiens hypothetical protein BC007901 (LOC91461), mRNA."
ILMN_1765419	GPLD1	-1.30462	0.001043	0.035567	-4.29279	Down	"Homo sapiens glycosylphosphatidylinositol specific phospholipase D1 (GPLD1), transcript variant 1, mRNA."
ILMN_1789702	GBE1	-0.82447	0.001049	0.035722	-4.28993	Down	"Homo sapiens glucan (1,4-alpha-), branching enzyme 1 (glycogen branching enzyme, Andersen disease, glycogen storage disease type IV) (GBE1), mRNA."
ILMN_1784602	CDKN1A	-1.17611	0.001053	0.035783	-4.28775	Down	"Homo sapiens cyclin-dependent kinase inhibitor 1A (p21, Cip1) (CDKN1A), transcript variant 1, mRNA."
ILMN_1748881	MRAS	-1.05431	0.001059	0.035825	-4.28441	Down	"Homo sapiens muscle RAS oncogene homolog (MRAS), transcript variant 1, mRNA."
ILMN_1851865	LOC158376	-0.7981	0.001062	0.035883	-4.28293	Down	"PREDICTED: Homo sapiens hypothetical protein LOC158376 (LOC158376), mRNA."
ILMN_1671489	PC	-1.42882	0.001069	0.035985	-4.27884	Down	"Homo sapiens pyruvate carboxylase (PC), nuclear gene encoding mitochondrial protein, transcript variant A, mRNA."
ILMN_2091846	FTHL2	-0.91046	0.001074	0.036033	-4.27629	Down	"Homo sapiens ferritin, heavy polypeptide-like 2 (FTHL2) on chromosome 1."
ILMN_1796755	ITGB5	-0.85966	0.001076	0.036033	-4.27538	Down	"Homo sapiens integrin, beta 5 (ITGB5), mRNA. XM_944688 XM_944693"
ILMN_1750100	TUBB4Q	-1.0731	0.001077	0.036033	-4.27455	Down	"Homo sapiens tubulin, beta polypeptide 4, member Q (TUBB4Q), mRNA."
ILMN_2358626	ADK	-0.8126	0.001102	0.036531	-4.26163	Down	"Homo sapiens adenosine kinase (ADK), transcript variant ADK-short, mRNA."
ILMN_1860665	HS.582097	-1.49446	0.001105	0.036581	-4.26007	Down	"RST41218 Athersys RAGE Library Homo sapiens cDNA, mRNA sequence"
ILMN_2410938	SMOC1	-0.8833	0.001111	0.036698	-4.25733	Down	"Homo sapiens SPARC related modular calcium binding 1 (SMOC1), transcript variant 1, mRNA."
ILMN_2402172	SEPT4	-0.78629	0.001112	0.036698	-4.25669	Down	"Homo sapiens septin 4 (SEPT4), transcript variant 3, mRNA."
ILMN_2395451	ASS1	-0.86187	0.001114	0.036725	-4.25588	Down	"Homo sapiens argininosuccinate synthetase 1 (ASS1), transcript variant 2, mRNA."

ILMN_2102670	GATA2	-0.75875	0.001133	0.037116	-4.24599	Down	"Homo sapiens GATA binding protein 2 (GATA2), mRNA."
ILMN_1745806	PEMT	-1.43413	0.001149	0.037422	-4.23817	Down	"Homo sapiens phosphatidylethanolamine N-methyltransferase (PEMT), nuclear gene encoding mitochondrial protein, transcript variant 1, mRNA."
ILMN_1808777	EHD2	-0.91484	0.001153	0.037422	-4.23652	Down	"Homo sapiens EH-domain containing 2 (EHD2), mRNA."
ILMN_1770663	KRT24	-1.10509	0.001154	0.037436	-4.23563	Down	"Homo sapiens keratin 24 (KRT24), mRNA."
ILMN_1812559	SLC7A6	-0.7428	0.001156	0.037436	-4.23477	Down	"Homo sapiens solute carrier family 7 (cationic amino acid transporter, y+ system), member 6 (SLC7A6), transcript variant 2, mRNA."
ILMN_1846306	HS.363526	-1.03949	0.00116	0.037503	-4.23277	Down	Homo sapiens cDNA clone IMAGE:4837650
ILMN_2312194	CYB5A	-0.85969	0.001168	0.03771	-4.22884	Down	"Homo sapiens cytochrome b5 type A (microsomal) (CYB5A), transcript variant 2, mRNA."
ILMN_1789648	SCGN	-0.99906	0.001198	0.038365	-4.21473	Down	"Homo sapiens secretagogin, EF-hand calcium binding protein (SCGN), mRNA."
ILMN_1691483	CLU	-0.91345	0.001203	0.038365	-4.21251	Down	"Homo sapiens clusterin (CLU), transcript variant 2, mRNA."
ILMN_1769556	C5ORF23	-1.79809	0.001203	0.038365	-4.21234	Down	"Homo sapiens chromosome 5 open reading frame 23 (C5orf23), mRNA."
ILMN_2369403	PALM2	-0.89108	0.001208	0.038502	-4.20995	Down	"Homo sapiens paralemmin 2 (PALM2), transcript variant 2, mRNA."
ILMN_1752294	PCDH9	-1.2459	0.001223	0.038746	-4.20299	Down	"Homo sapiens protocadherin 9 (PCDH9), transcript variant 1, mRNA."
ILMN_1696066	CARS	-0.80971	0.001228	0.038851	-4.20073	Down	"Homo sapiens cysteinyl-tRNA synthetase (CARS), transcript variant 4, mRNA."
ILMN_1787843	HSDL2	-0.72982	0.001233	0.038953	-4.19843	Down	"Homo sapiens hydroxysteroid dehydrogenase like 2 (HSDL2), mRNA."
ILMN_1673522	MOCOS	-0.85942	0.001237	0.038995	-4.19676	Down	"Homo sapiens molybdenum cofactor sulfurase (MOCOS), mRNA."
ILMN_1803094	PDGFD	-1.09821	0.001243	0.039123	-4.19418	Down	"Homo sapiens platelet derived growth factor D (PDGFD), transcript variant 2, mRNA."
ILMN_1671777	FGF13	-0.95577	0.001247	0.039251	-4.19196	Down	"Homo sapiens fibroblast growth factor 13 (FGF13), transcript variant 1A, mRNA."
ILMN_1714170	SPSB1	-0.76765	0.001252	0.039307	-4.19005	Down	"Homo sapiens splA/ryanodine receptor domain and SOCS box containing 1 (SPSB1), mRNA."
ILMN_1795963	OKL38	-0.90163	0.001254	0.039331	-4.18895	Down	"Homo sapiens pregnancy-induced growth inhibitor (OKL38), transcript variant 1, mRNA."
ILMN_1797201	KRTAP10-2	-1.2139	0.00126	0.039431	-4.18642	Down	"Homo sapiens keratin associated protein 10-2 (KRTAP10-2), mRNA."
ILMN_1807169	TINAGL1	-0.82684	0.001273	0.039588	-4.18062	Down	"Homo sapiens tubulointerstitial nephritis antigen-like 1 (TINAGL1), mRNA."
ILMN_1707077	SORT1	-1.14345	0.001274	0.039588	-4.18029	Down	"Homo sapiens sortilin 1 (SORT1), mRNA."
ILMN_2111255	VIT	-1.25067	0.001275	0.039588	-4.17975	Down	"Homo sapiens vitrin (VIT), mRNA."
ILMN_1743784	SHMT1	-0.86891	0.001287	0.039662	-4.17464	Down	"Homo sapiens serine hydroxymethyltransferase 1 (soluble) (SHMT1), transcript variant 1, mRNA."
ILMN_2290118	MEGF9	-0.84591	0.001305	0.039923	-4.16657	Down	"Homo sapiens multiple EGF-like-domains 9 (MEGF9), mRNA."
ILMN_1812721	LOC728014	-0.8285	0.001306	0.039923	-4.16624	Down	"PREDICTED: Homo sapiens similar to huntingtin interacting protein 1 related (LOC728014), mRNA."
ILMN_1662795	CA2	-0.76388	0.001312	0.040067	-4.1635	Down	"Homo sapiens carbonic anhydrase II (CA2), mRNA."
ILMN_1704656	PPP2R1B	-1.02024	0.001347	0.040661	-4.14876	Down	"Homo sapiens protein phosphatase 2 (formerly 2A), regulatory subunit A, beta isoform (PPP2R1B), transcript variant 2, mRNA."
ILMN_1763837	ANPEP	-1.20973	0.001352	0.040668	-4.14689	Down	"Homo sapiens alanyl (membrane) aminopeptidase (aminopeptidase N, aminopeptidase M, microsomal aminopeptidase, CD13, p150) (ANPEP), mRNA."
ILMN_1792495	AHNAK	-1.41447	0.001365	0.040905	-4.14149	Down	"Homo sapiens AHNAK nucleoprotein (AHNAK), transcript variant 1, mRNA."
ILMN_1656638	BHMT	-0.85305	0.001371	0.041012	-4.13897	Down	"Homo sapiens betaine-homocysteine methyltransferase (BHMT), mRNA."
ILMN_2413898	MCM10	-0.81846	0.001437	0.042265	-4.11266	Down	"Homo sapiens minichromosome maintenance complex component 10 (MCM10), transcript variant 2, mRNA."
ILMN_1698533	IDH3A	-0.8011	0.001458	0.042631	-4.10437	Down	"Homo sapiens isocitrate dehydrogenase 3 (NAD+) alpha(IDH3A), nuclear gene encoding mitochondrial protein, mRNA."

ILMN_3244070	ABCC6P2	-0.72823	0.001461	0.042684	-4.10333	Down	"Homo sapiens ATP-binding cassette, sub-family C, member 6 pseudogene 2 (ABCC6P2), non-coding RNA."
ILMN_1768598	LAIR1	-0.74324	0.001468	0.042795	-4.10085	Down	"Homo sapiens leukocyte-associated immunoglobulin-like receptor 1 (LAIR1), transcript variant b, mRNA."
ILMN_2170080	CIDECP	-1.02061	0.001477	0.042996	-4.0972	Down	"Homo sapiens cell death-inducing DFFA-like effector c pseudogene (CIDECP), non-coding RNA."
ILMN_1776363	ANK2	-0.75797	0.00148	0.043033	-4.0961	Down	"Homo sapiens ankyrin 2, neuronal (ANK2), transcript variant 2, mRNA."
ILMN_1794017	SERTAD1	-0.75632	0.001489	0.043113	-4.09294	Down	"Homo sapiens SERTA domain containing 1 (SERTAD1), mRNA."
ILMN_1701558	MAP1A	-1.40779	0.001495	0.043271	-4.09056	Down	"Homo sapiens microtubule-associated protein 1A (MAP1A), mRNA."
ILMN_2112256	TNFRSF4	-1.08801	0.001497	0.043294	-4.08991	Down	"Homo sapiens tumor necrosis factor receptor superfamily, member 4 (TNFRSF4), mRNA."
ILMN_1662557	MAP7D3	-0.79584	0.001505	0.043452	-4.08686	Down	"Homo sapiens MAP7 domain containing 3 (MAP7D3), mRNA."
ILMN_1651737	LOC644689	-1.18878	0.001522	0.043689	-4.08059	Down	"PREDICTED: Homo sapiens similar to N-acetylated-alpha-linked acidic dipeptidase II (NAALADase II) (LOC644689), mRNA."
ILMN_1697820	HINT2	-0.73741	0.001524	0.043689	-4.07975	Down	"Homo sapiens histidine triad nucleotide binding protein 2 (HINT2), mRNA."
ILMN_1825249	HS.453381	-1.27632	0.001539	0.043921	-4.07443	Down	"in27e08.x1 Human Fetal Pancreas 1B Homo sapiens cDNA clone IMAGE: 3, mRNA sequence"
ILMN_1775235	AFF3	-0.85233	0.001542	0.043954	-4.07334	Down	"Homo sapiens AF4/FMR2 family, member 3 (AFF3), transcript variant 2, mRNA."
ILMN_1775268	HECW2	-0.97799	0.001551	0.044165	-4.07	Down	"Homo sapiens HECT, C2 and WW domain containing E3 ubiquitin protein ligase 2 (HECW2), mRNA."
ILMN_1749907	LOC441241	-1.06899	0.001581	0.044687	-4.05943	Down	"PREDICTED: Homo sapiens chaperonin containing TCP1, subunit 6A (zeta 1)-like (LOC441241), mRNA."
ILMN_2304495	PPP1R1B	-0.85043	0.001587	0.04474	-4.05711	Down	"Homo sapiens protein phosphatase 1, regulatory (inhibitor) subunit 1B (dopamine and cAMP regulated phosphoprotein, DARPP-32) (PPP1R1B), transcript variant 2, mRNA."
ILMN_1708778	ASS1	-0.90496	0.001608	0.045241	-4.04991	Down	"Homo sapiens argininosuccinate synthetase 1 (ASS1), transcript variant 1, mRNA."
ILMN_1773395	RDH5	-1.03496	0.001629	0.045605	-4.04281	Down	"Homo sapiens retinol dehydrogenase 5 (11-cis/9-cis) (RDH5), mRNA."
ILMN_1803652	C9ORF91	-0.78129	0.001639	0.045769	-4.03916	Down	"Homo sapiens chromosome 9 open reading frame 91 (C9orf91), mRNA."
ILMN_2052135	LOH3CR2A	-0.96644	0.001648	0.045955	-4.03624	Down	"Homo sapiens loss of heterozygosity, 3, chromosomal region 2, gene A (LOH3CR2A), mRNA."
ILMN_1739423	RN7SK	-4.66483	0.001664	0.046224	-4.03071	Down	"Homo sapiens RNA, 7SK small nuclear (RN7SK), non-coding RNA."
ILMN_1754538	C10ORF58	-0.7354	0.00167	0.046262	-4.02901	Down	"Homo sapiens chromosome 10 open reading frame 58 (C10orf58), transcript variant 1, mRNA."
ILMN_1707727	ANGPTL4	-1.32658	0.001702	0.046916	-4.01842	Down	"Homo sapiens angiopoietin-like 4 (ANGPTL4), transcript variant 1, mRNA."
ILMN_1723971	SLC29A1	-0.95111	0.001734	0.047458	-4.00808	Down	"Homo sapiens solute carrier family 29 (nucleoside transporters), member 1 (SLC29A1), nuclear gene encoding mitochondrial protein, transcript variant 4, mRNA."
ILMN_2141118	C15ORF59	-0.77492	0.00175	0.047704	-4.00284	Down	"Homo sapiens chromosome 15 open reading frame 59 (C15orf59), mRNA."
ILMN_1795838	C4ORF19	-1.14745	0.001758	0.04776	-4.00032	Down	"Homo sapiens chromosome 4 open reading frame 19 (C4orf19), mRNA."
ILMN_1690682	B3GALT4	-0.82005	0.001761	0.047814	-3.99932	Down	"Homo sapiens UDP-Gal:betaGlcNAc beta 1,3-galactosyltransferase, polypeptide 4 (B3GALT4), mRNA."
ILMN_1767135	SOS1	-1.13327	0.001768	0.047884	-3.99723	Down	"Homo sapiens son of sevenless homolog 1 (Drosophila) (SOS1), mRNA."
ILMN_1807244	STGC3	-0.78053	0.001788	0.048243	-3.99087	Down	"PREDICTED: Homo sapiens hypothetical STGC3 (STGC3), misc RNA."
ILMN_1809947	TMEM155	-0.75043	0.001803	0.048311	-3.98638	Down	"Homo sapiens transmembrane protein 155 (TMEM155), mRNA."
ILMN_2391150	FILIP1L	-0.93088	0.001817	0.048433	-3.9821	Down	"Homo sapiens filamin A interacting protein 1-like (FILIP1L), transcript variant 3, mRNA."
ILMN_1656378	NMT2	-0.83905	0.001821	0.048494	-3.98076	Down	"Homo sapiens N-myristoyltransferase 2 (NMT2), mRNA."
ILMN_1705743	FLJ10357	-0.75163	0.001835	0.048673	-3.97657	Down	"Homo sapiens hypothetical protein FLJ10357 (FLJ10357), mRNA."

ILMN_1804207	MRPL4	-1.00009	0.001856	0.049089	-3.97026	Down	"Homo sapiens mitochondrial ribosomal protein L4 (MRPL4), nuclear gene encoding mitochondrial protein, transcript variant 1, mRNA."
ILMN_1730906	FILIP1L	-0.79699	0.001894	0.049692	-3.95908	Down	"Homo sapiens filamin A interacting protein 1-like (FILIP1L), transcript variant 1, mRNA."
ILMN_1779597	CIDEC	-0.87612	0.001907	0.049885	-3.95518	Down	"Homo sapiens cell death-inducing DFFA-like effector c (CIDEC), mRNA."

Table S2 The enriched pathway terms of the up-regulated differentially expressed genes

Pathway ID	Pathway Name	P-value	Adjusted P-value	Z-score	Combined Score	Gene Count	Genes
			**KEGG**				
hsa04610	Complement and coagulation cascades	2.33393E-06	0.000373429	-1.824138353	23.65534726	10	C3,PROCR,VTN,C7, C1R,CFI,PLAT,C4B PB,CFB,
hsa04514	Cell adhesion molecules (CAMs	0.001540375	0.123230014	-1.701720853	11.01988355	09	NTNG1,CLDN11,C NTNAP2,CDH3,CD H2,CLDN15, SDC4,NRXN2,CLD N1
hsa05150	Staphylococcus aureusinfection	0.004071977	0.217172094	-1.749095362	9.626367946	05	C3,C1R,CFI,CFB,C2
hsa05133	Pertussis	0.01377431	0.459483075	-1.640674923	7.030210025	05	C3,CXCL6,C1R,C4B PB,C2
hsa04340	Hedgehog signalingpathway	0.014602744	0.459483075	-1.636676372	6.91748767	04	WNT2B,WNT5A,GA S1,LRP2
hsa04550	Signaling pathways regulating pluripotency of stem cells	0.018477951	0.459483075	-1.704080823	6.801288352	07	MEIS1,WNT2B,FZD 7,WNT5A,AXIN2,IS L1,FGFR3
hsa05217	Basal cell carcinoma	0.020102385	0.459483075	-1.654853579	6.46537531	04	WNT2B,FZD7,WNT 5A,AXIN2
hsa04390	Hippo signalingpathway	0.026493449	0.494590892	-1.568092351	5.693520346	07	DLG2,WNT2B,FZD 7,WWC1,WNT5A,A XIN2,PRKCZ
hsa05205	Proteoglycans in cancer	0.039599395	0.494590892	-1.728622226	5.581619934	08	VTN,WNT2B,SDC4, ERBB3,FZD7,WNT5 A,HPSE, EZR
hsa00350	Tyrosine metabolism	0.028051356	0.494590892	-1.479412463	5.287003392	03	ALDH1A3,ADH1C, AOX1
			**WikiPathways 2016**				
WP545	Complement Activation	4.74484E-05	0.00489392	-2.040134457	20.3113088	05	
